# Supplementary figures and images for: Effects of canagliflozin on growth and metabolic reprograming in hepatocellular carcinoma cells: Multi-omics analysis of metabolomics and absolute quantification proteomics (iMPAQT)
Source: PLoS One. 2020 Apr 28;15(4):e0232283. doi: 10.1371/journal.pone.0232283 (PMC7188283; doi:10.1371/journal.pone.0232283)

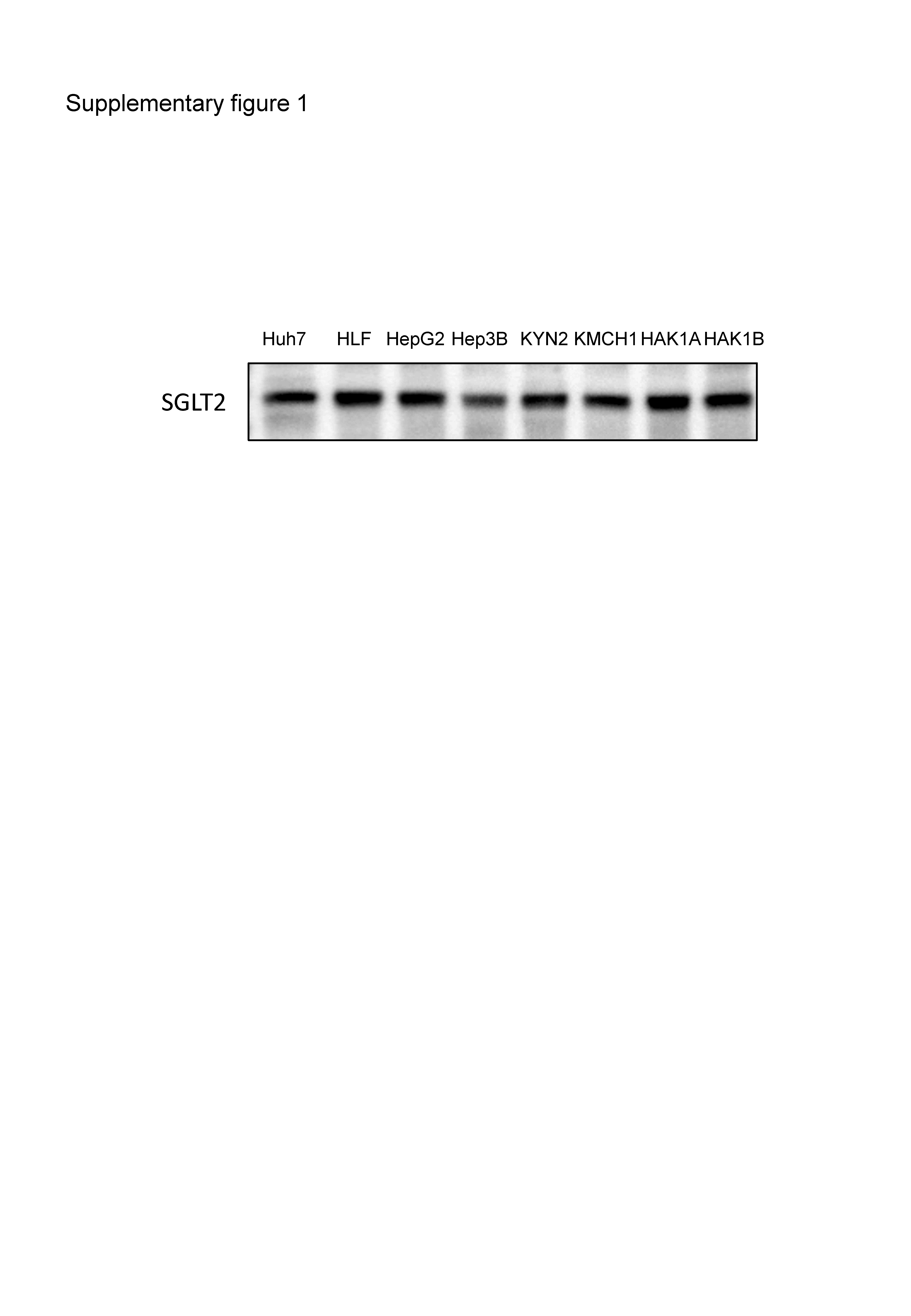

Supplement: S1 Fig — Abbreviations: CANA, canagliflozin; SGLT1, sodium-glucose cotransporter 1; GLUT, glucose transporter. (TIFF) [file pone.0232283.s001.tiff]

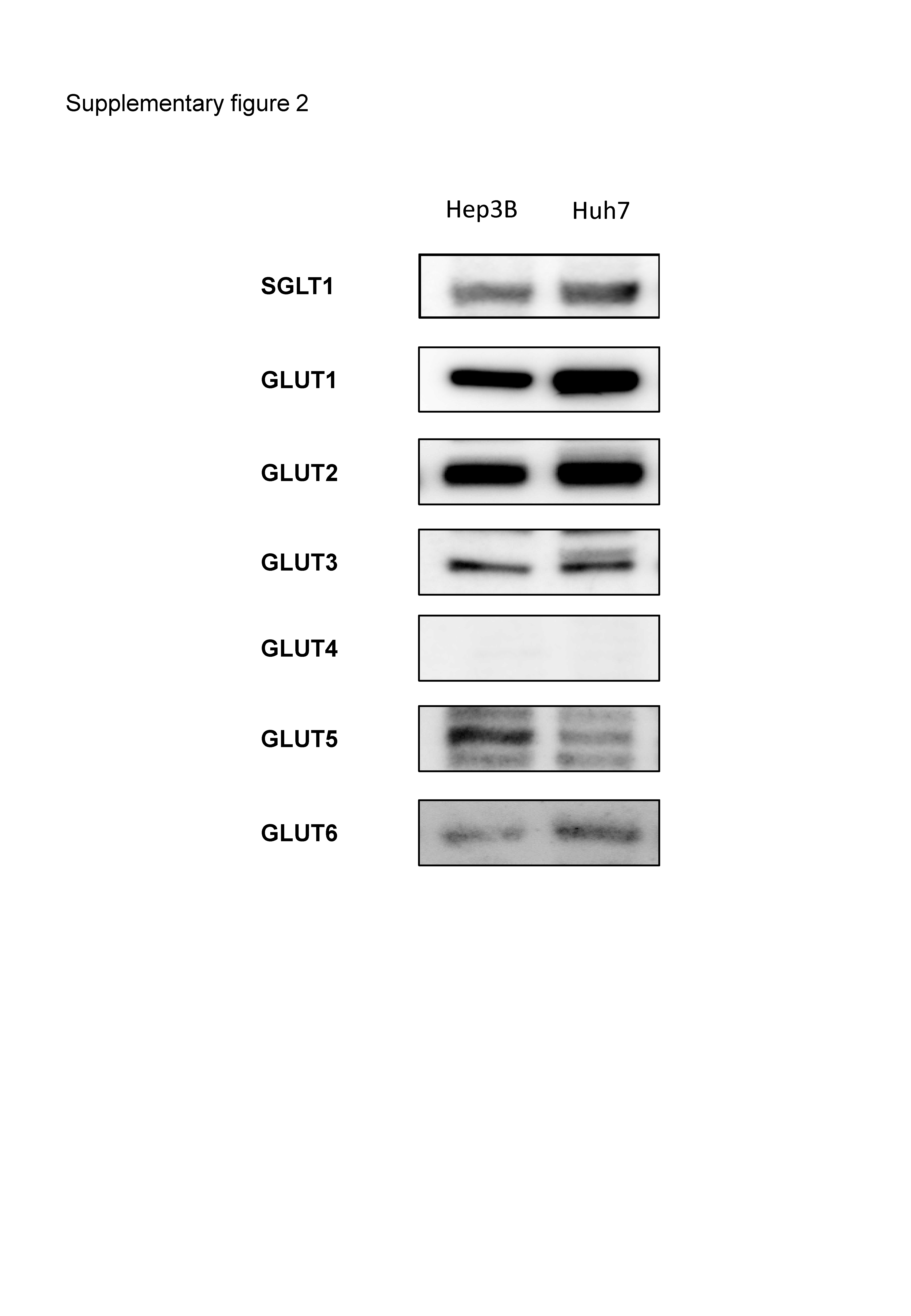

Supplement: S2 Fig — Abbreviations: CANA, canagliflozin; SGLT1, sodium-glucose cotransporter 1; GLUT, glucose transporter. (TIFF) [file pone.0232283.s002.tiff]

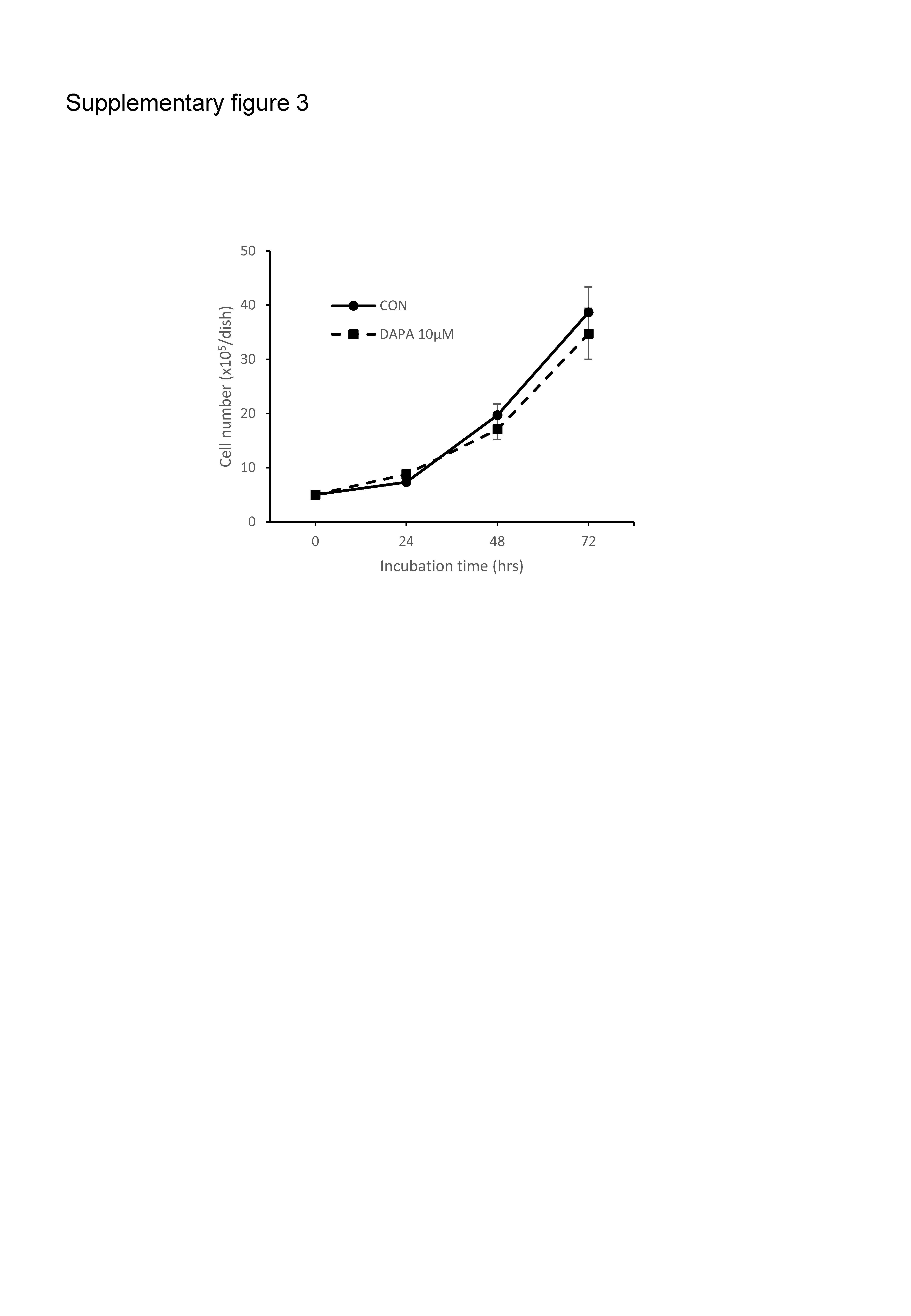

Supplement: S3 Fig — * P<0.01. Abbreviations: CON, control; DAPA, dapagliflozin. (TIFF) [file pone.0232283.s003.tiff]

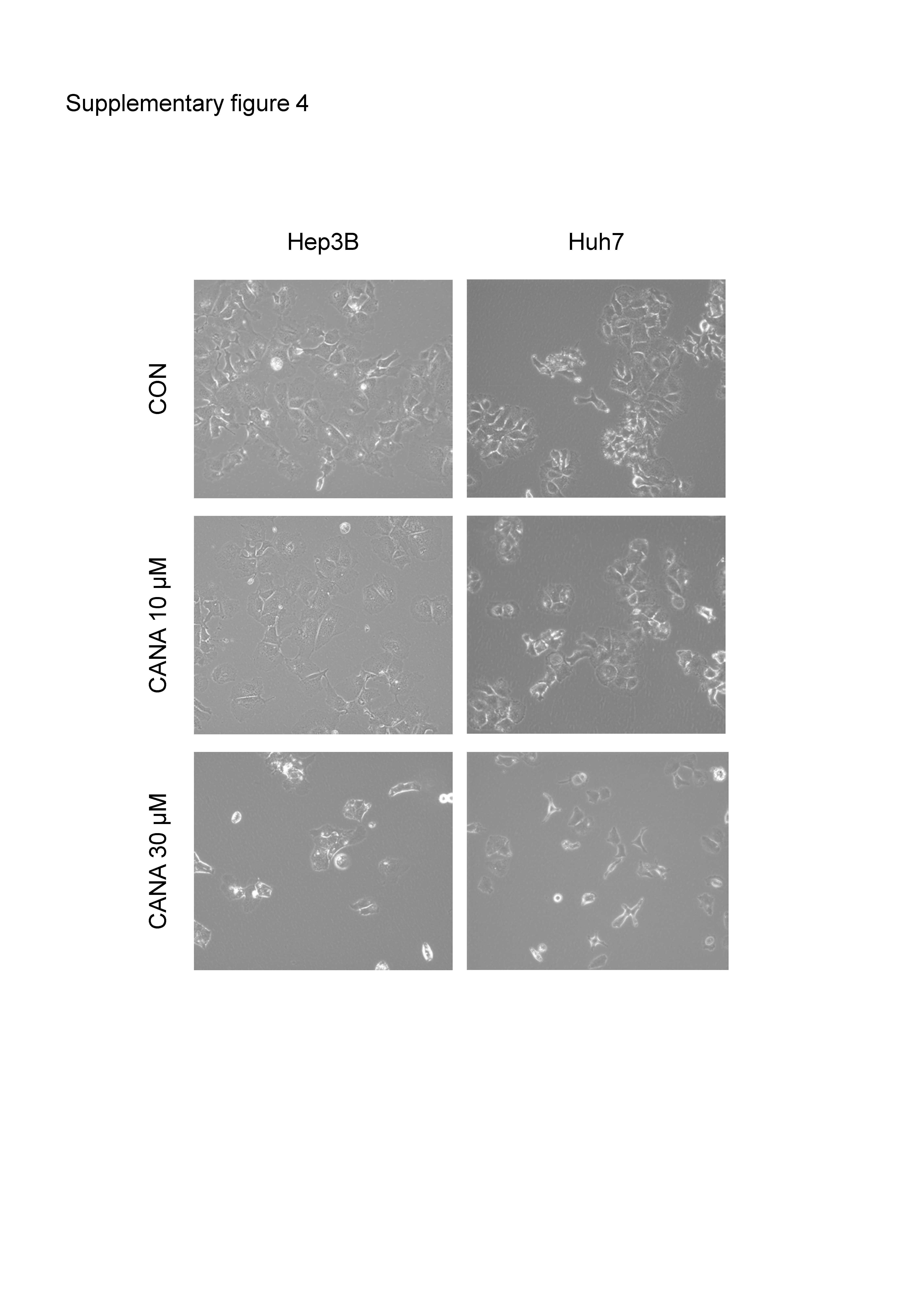

Supplement: S4 Fig — Scale bar = 50 μm. Abbreviations: CON, control; CANA, canagliflozin. (TIFF) [file pone.0232283.s004.tiff]

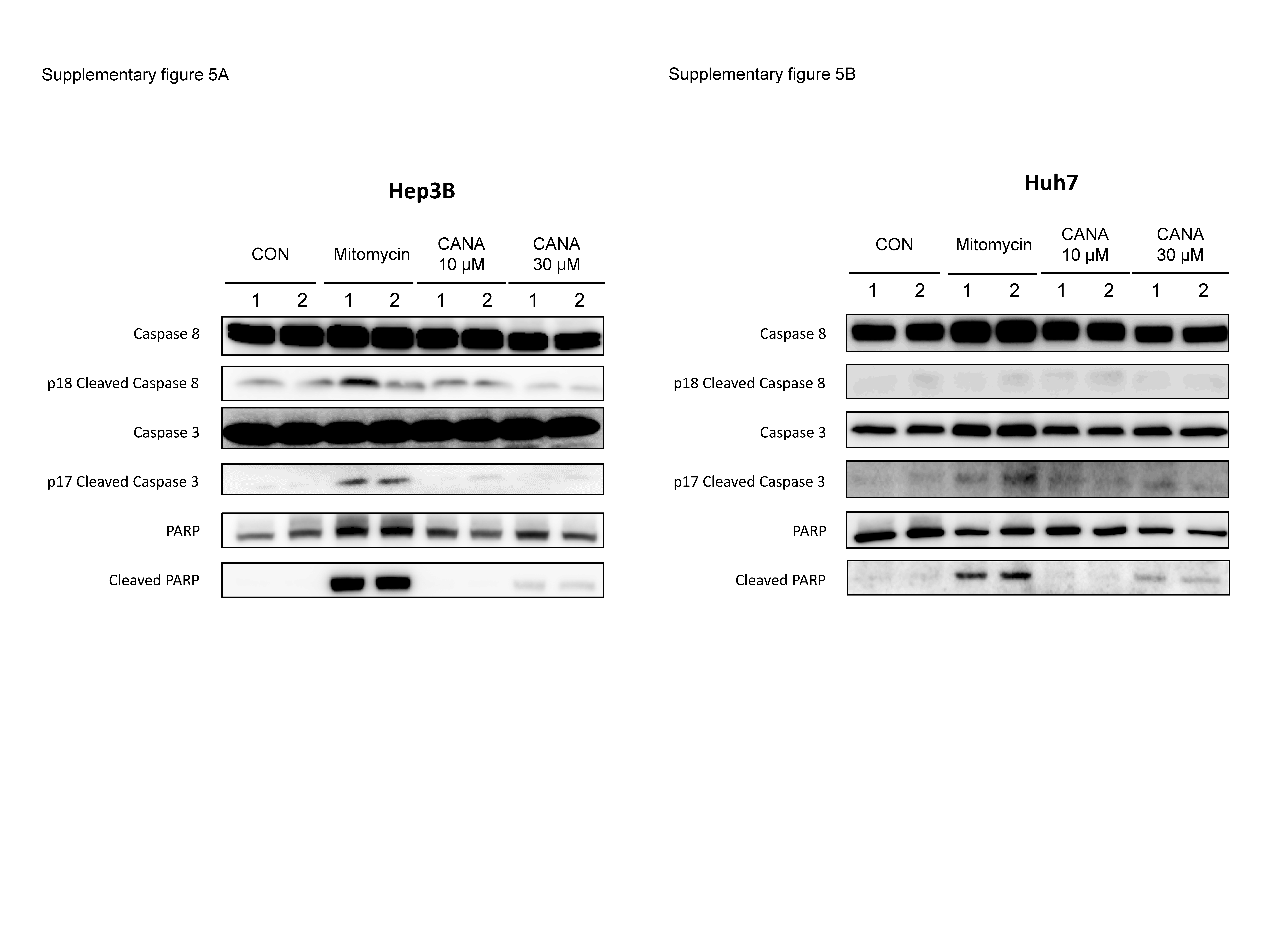

Supplement: S5 Fig — Abbreviations: CON, control; CANA, canagliflozin; PARP, poly adenosine diphosphate-ribose polymerase. (TIFF) [file pone.0232283.s005.tiff]

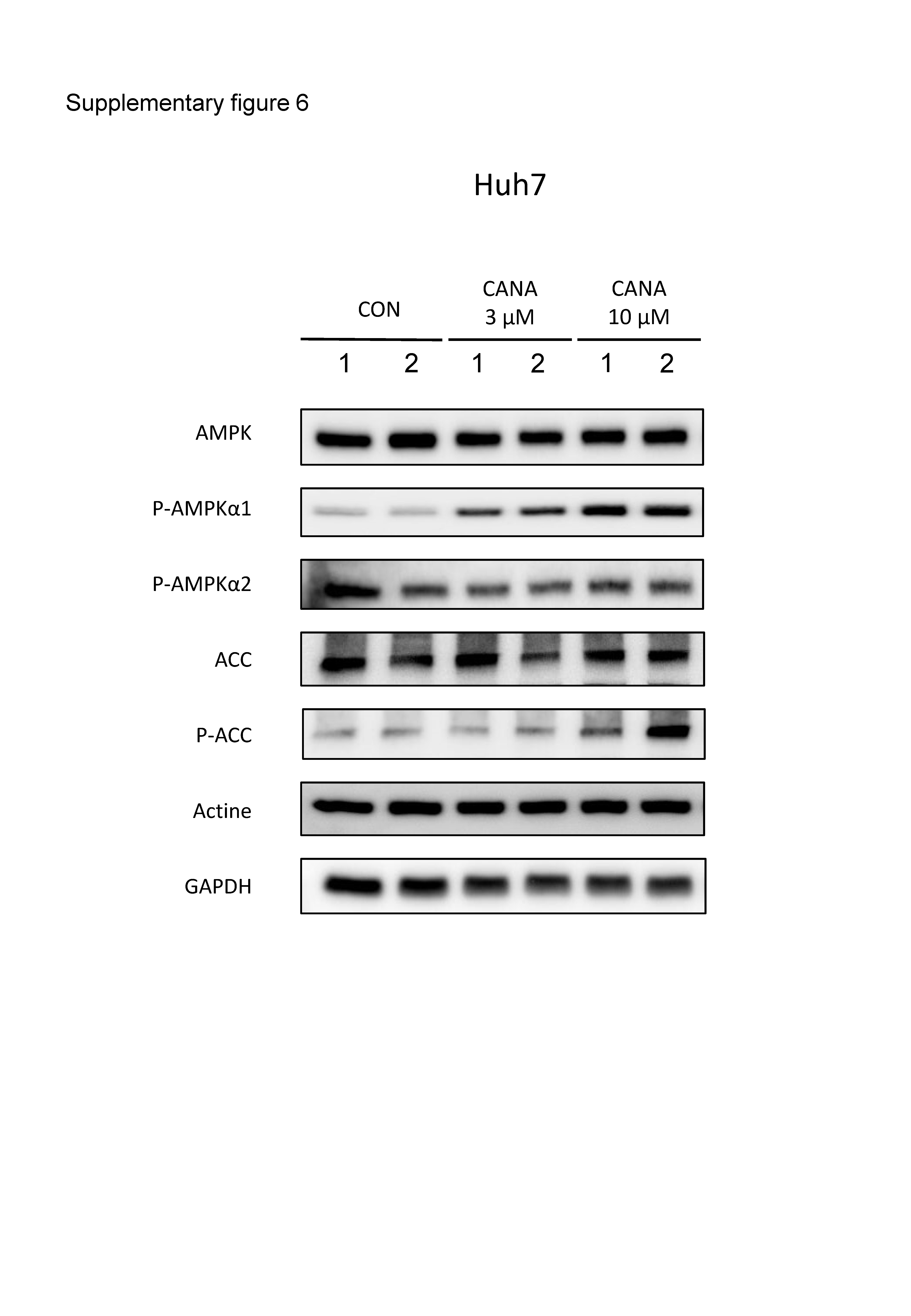

Supplement: S6 Fig — Abbreviations: CON, control; CANA, canagliflozin; AMPK, AMP-activated protein kinase; ACC, acetyl-CoA carboxylase. (TIFF) [file pone.0232283.s006.tiff]

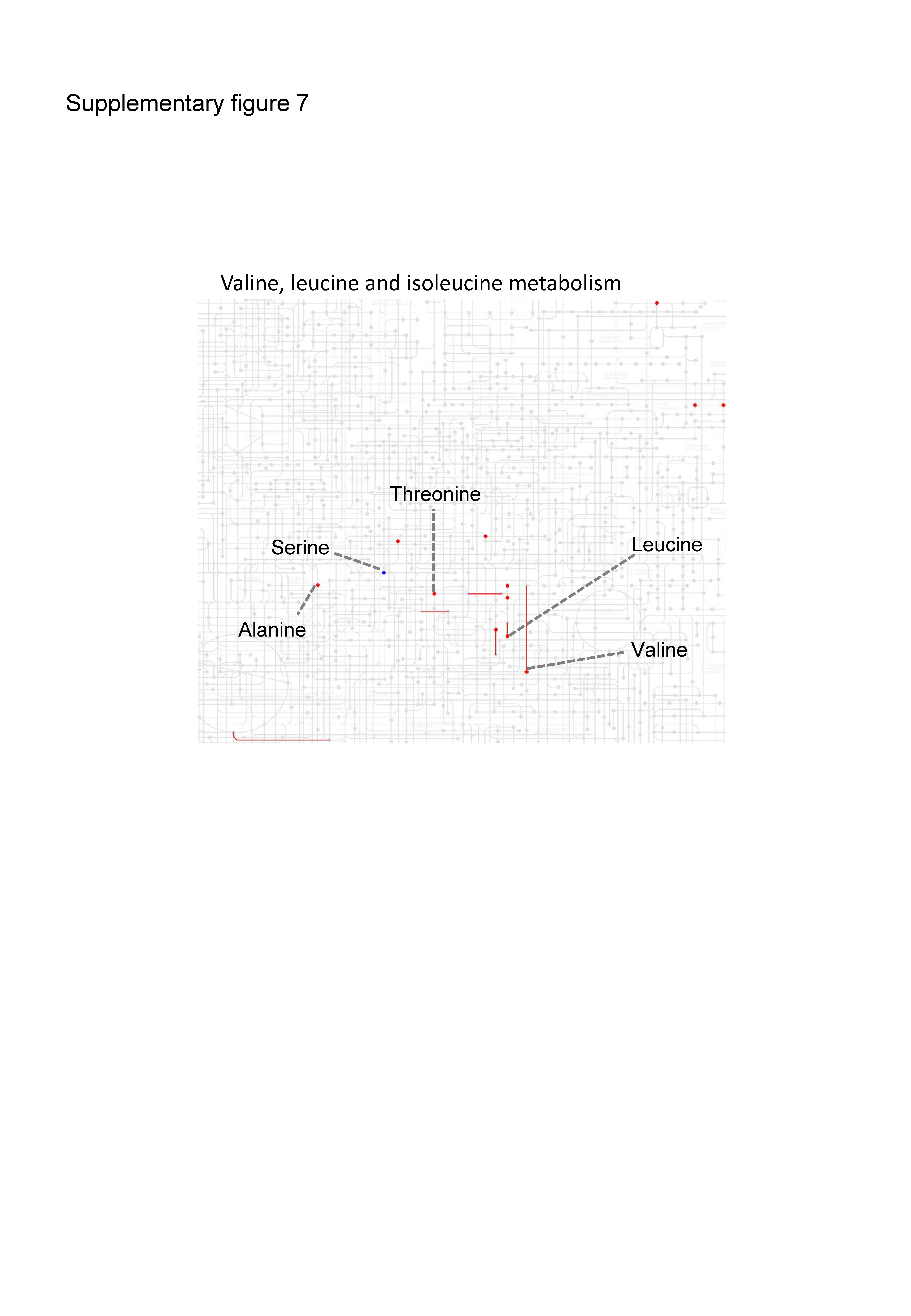

Supplement: S7 Fig — Red line indicates an up-regulated pathway. Red circle indicates an up-regulated metabolite. Blue circle indicates a down-regulated metabolite. (TIFF) [file pone.0232283.s007.tiff]

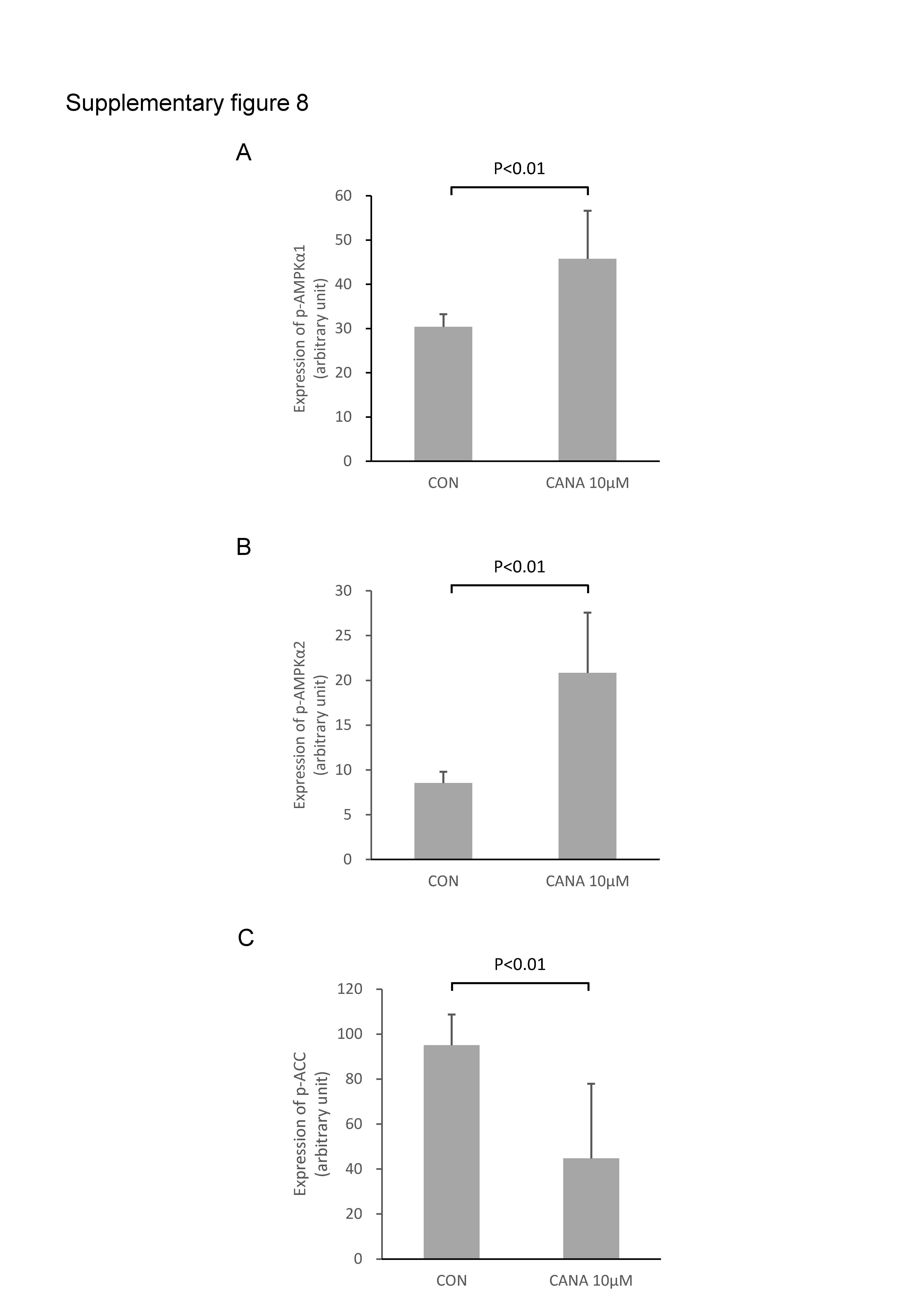

Supplement: S8 Fig — Abbreviations: CON, control; CANA, canagliflozin; AMPK, AMP-activated protein kinase; ACC, acetyl-CoA carboxylase. (TIFF) [file pone.0232283.s008.tiff]
