# Supplementary material for: Effects of canagliflozin on growth and metabolic reprograming in hepatocellular carcinoma cells: Multi-omics analysis of metabolomics and absolute quantification proteomics (iMPAQT)
Source: PLoS One. 2020 Apr 28;15(4):e0232283. doi: 10.1371/journal.pone.0232283 (PMC7188283; doi:10.1371/journal.pone.0232283)

Figure 1

A

Antibody-a

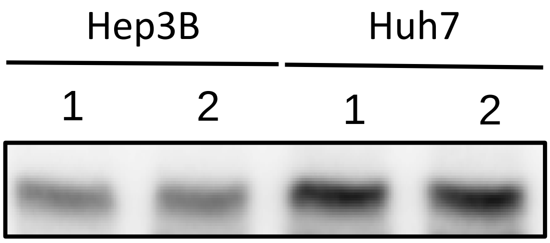

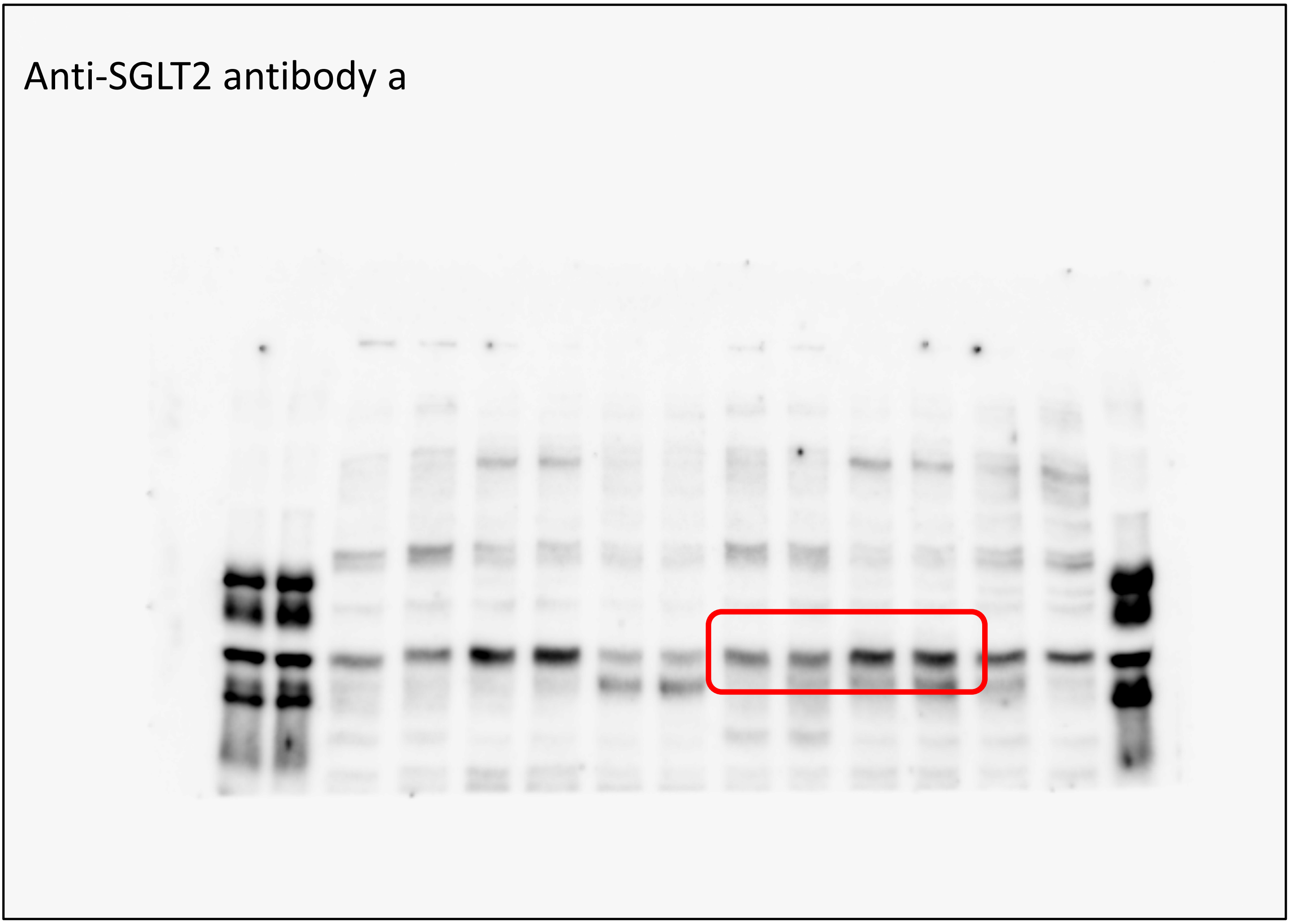

Figure 1

A

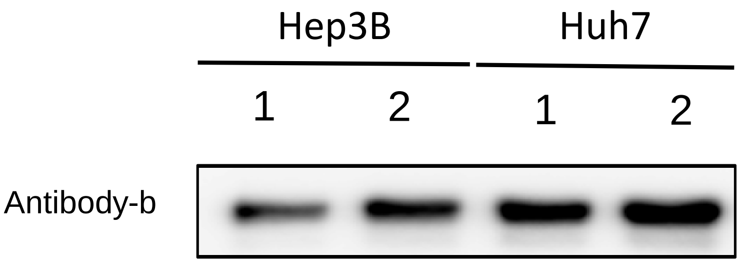

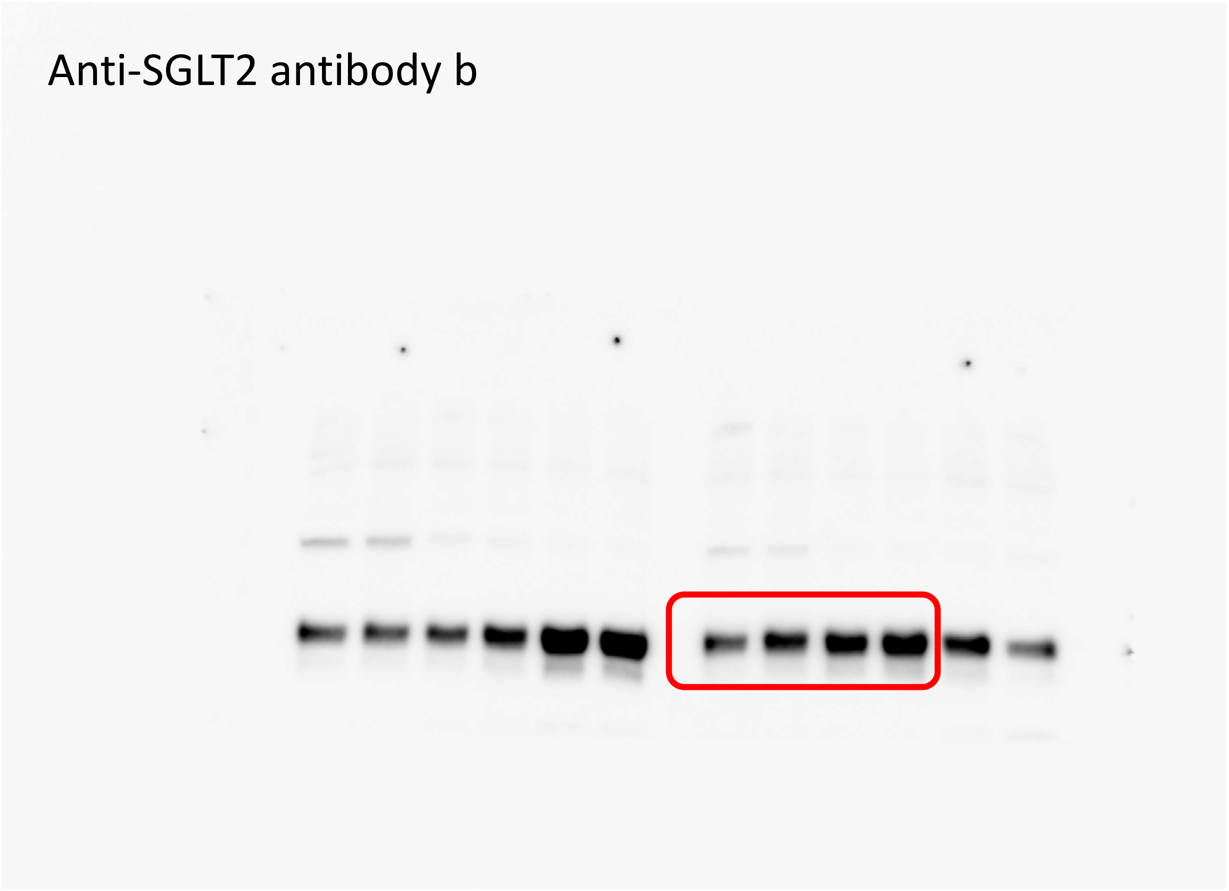

Figure 1

A

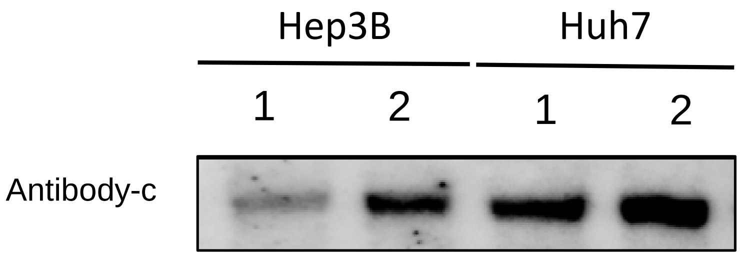

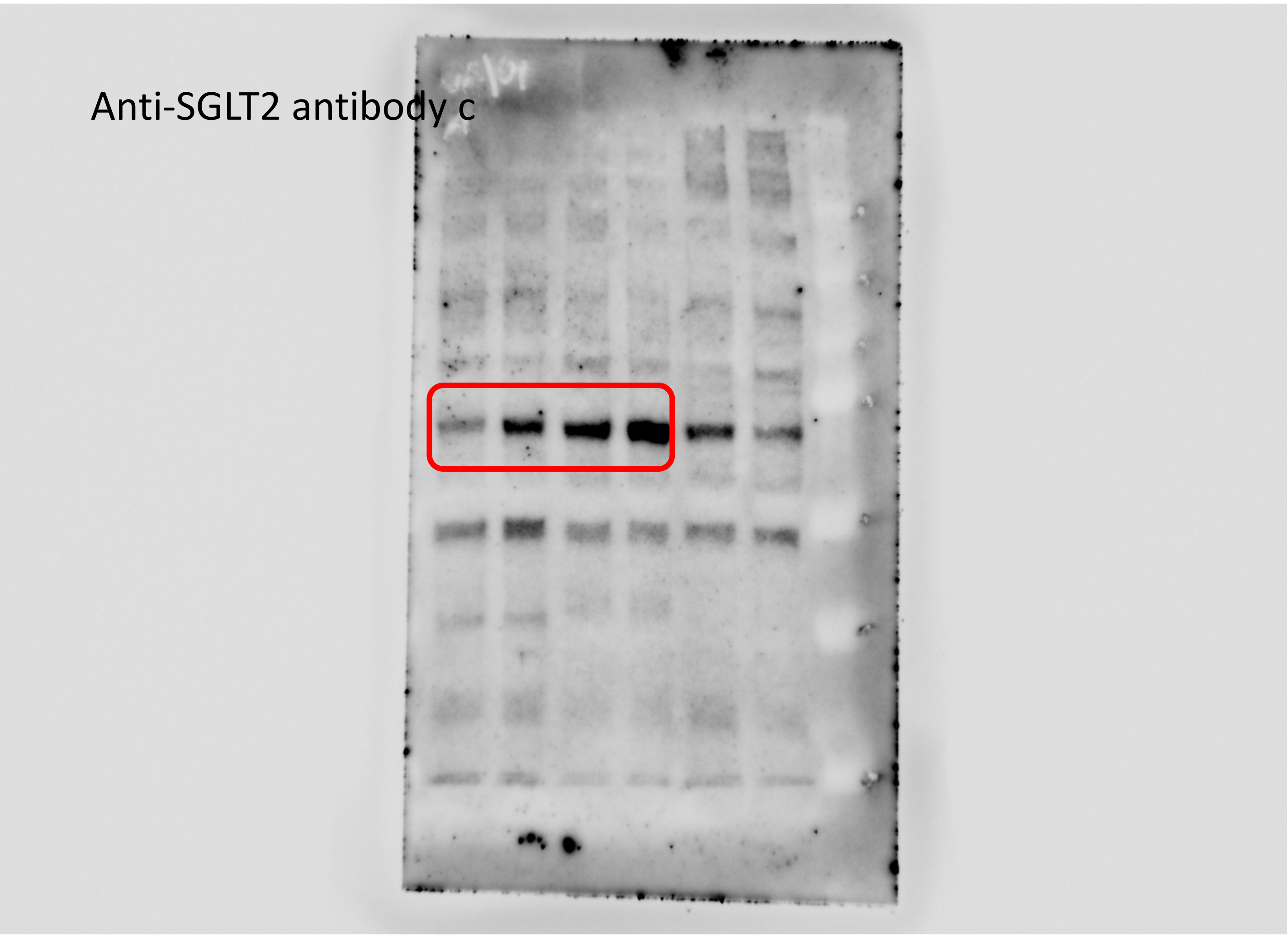

Figure 1

A

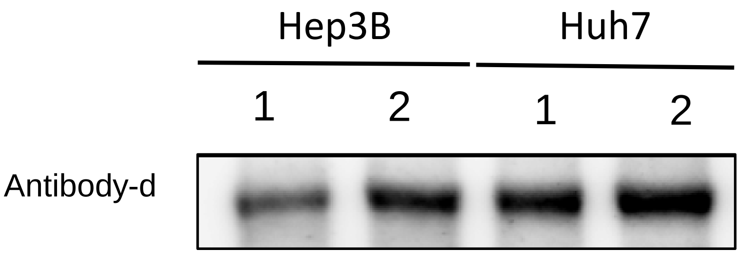

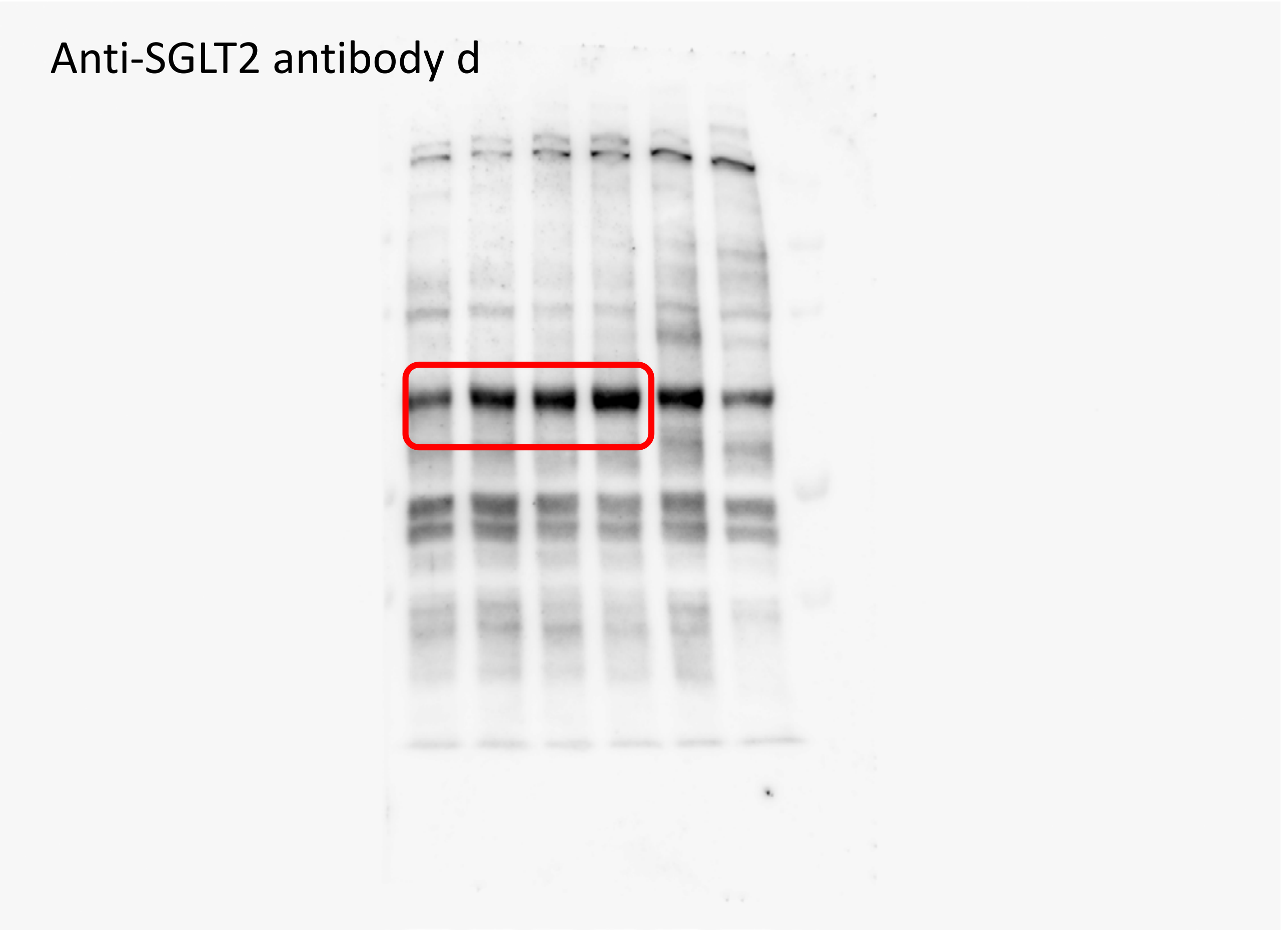

Figure 1

A

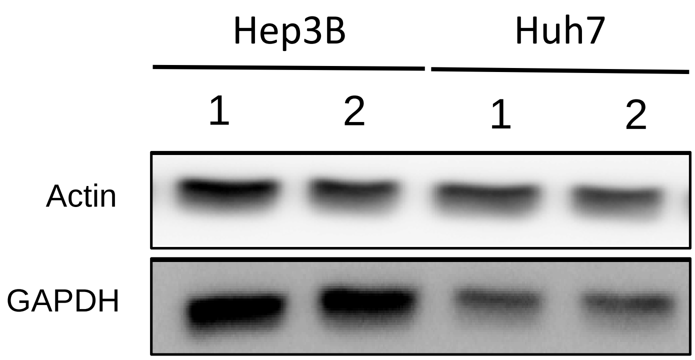
Hep3B
Huh7
1
2
1
2
Actin
GAPDH

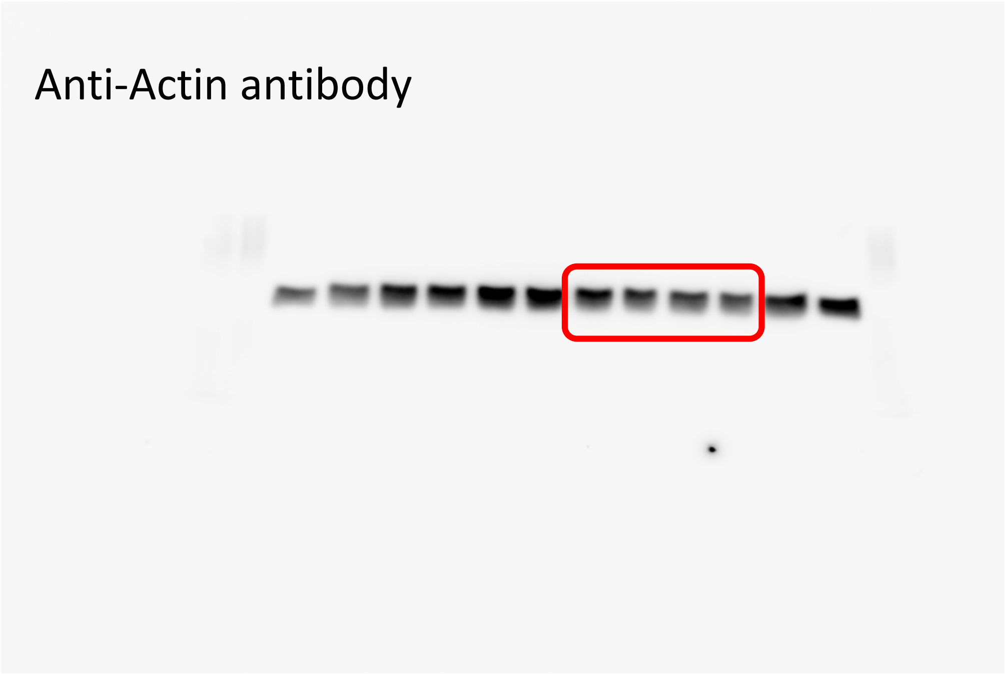
Anti-Actin antibody

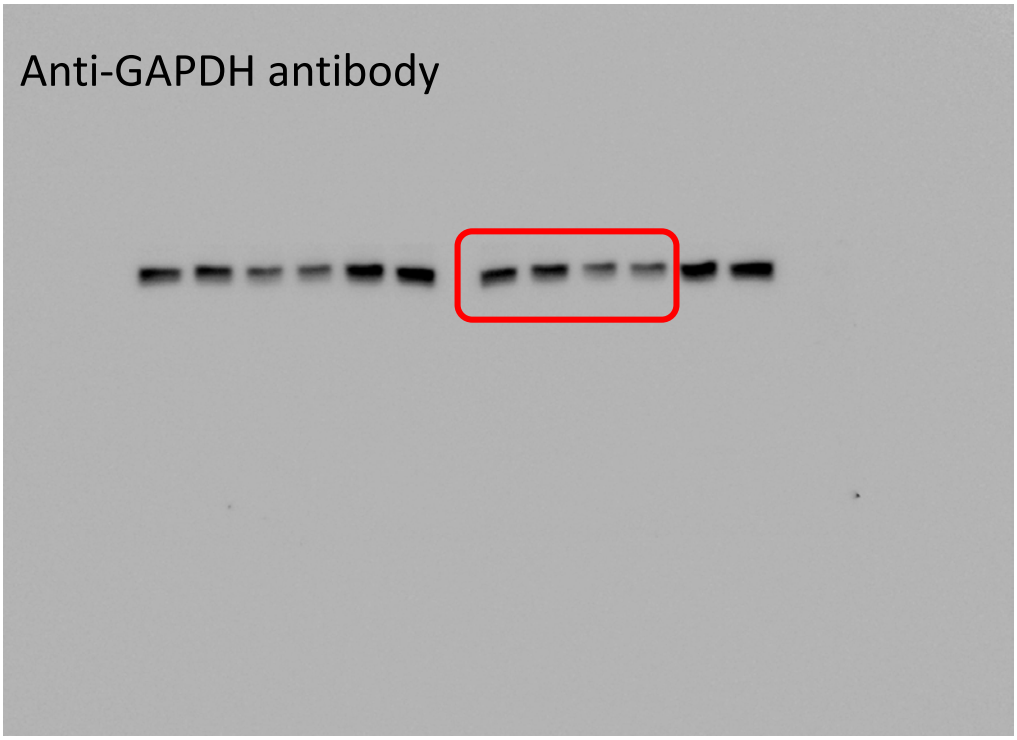
Anti-GAPDH antibody

Figure 1

B

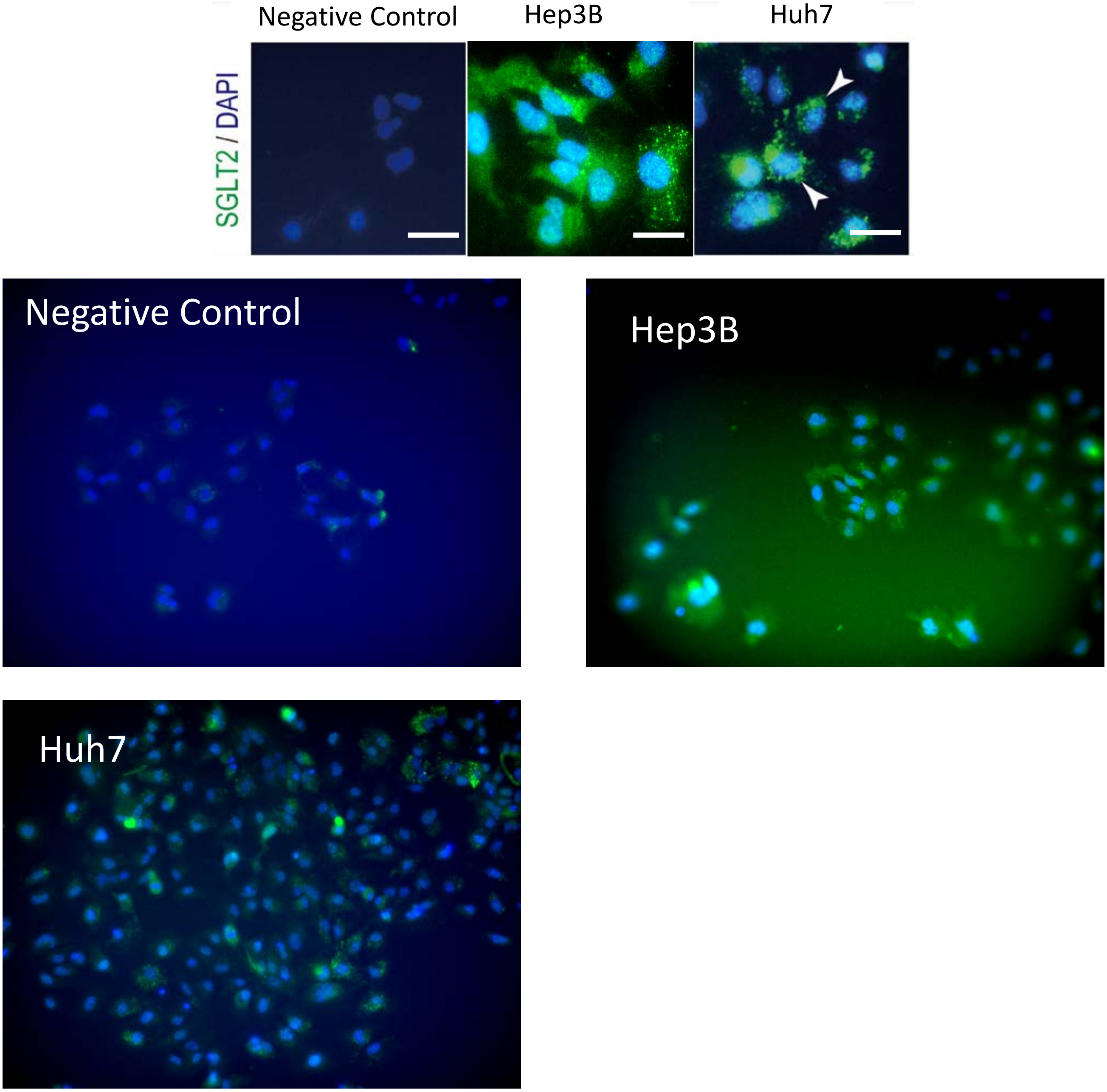
Negative Control
Hep3B
Huh7
SGLT2 / DAPI
Negative Control
Hep3B
Huh7

Figure 1

C

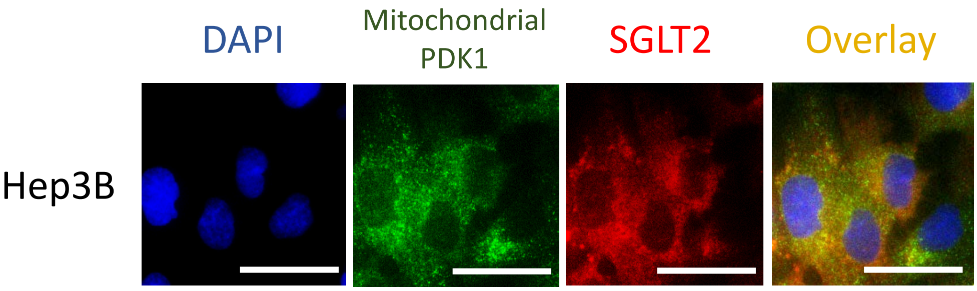
DAPI
Mitochondrial PDK1
SGLT2
Overlay
Hep3B

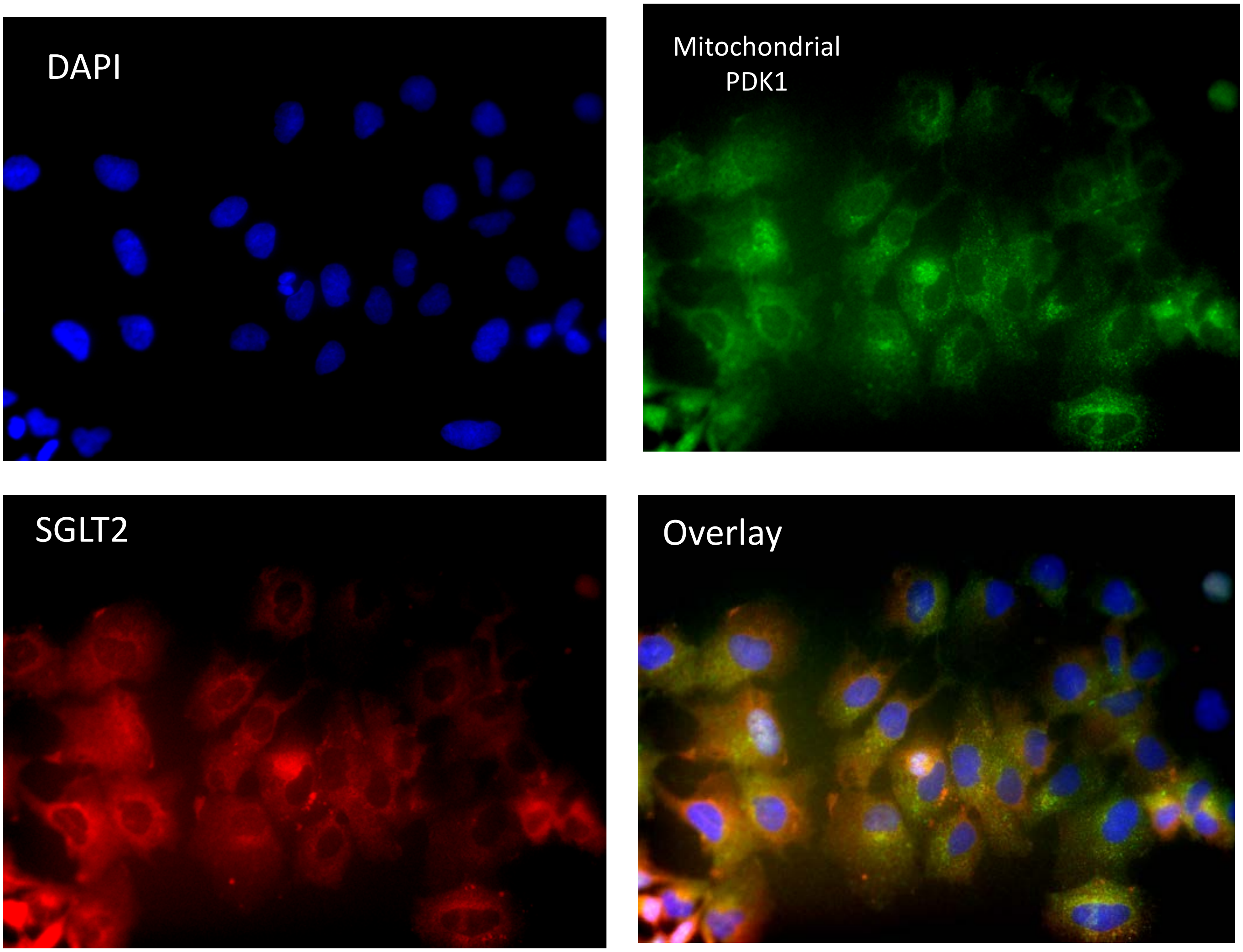
DAPI
Mitochondrial PDK1
SGLT2
Overlay

Figure 1

C

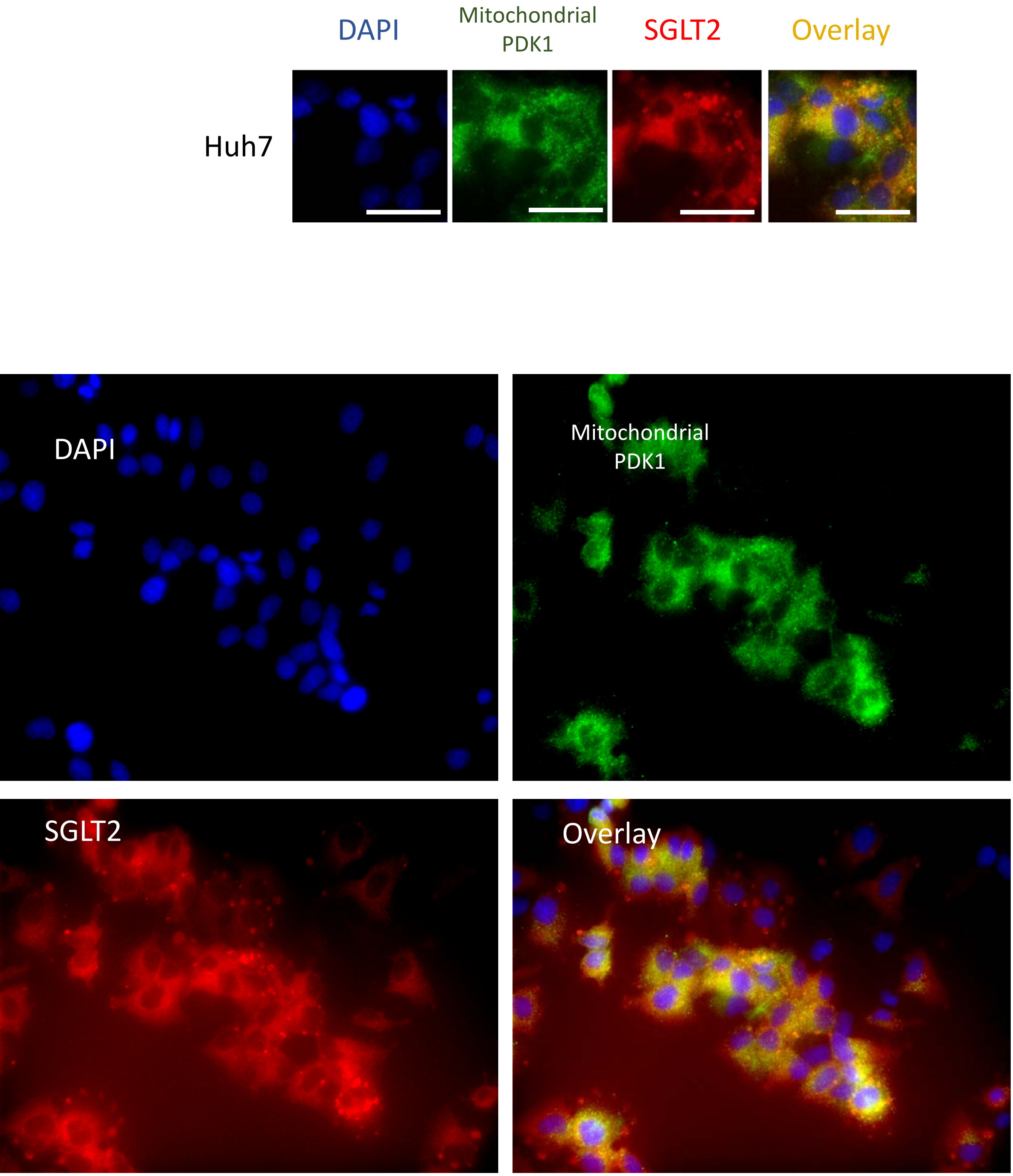
DAPI
Mitochondrial PDK1
SGLT2
Overlay
Huh7
DAPI
Mitochondrial PDK1
SGLT2
Overlay

Figure 3

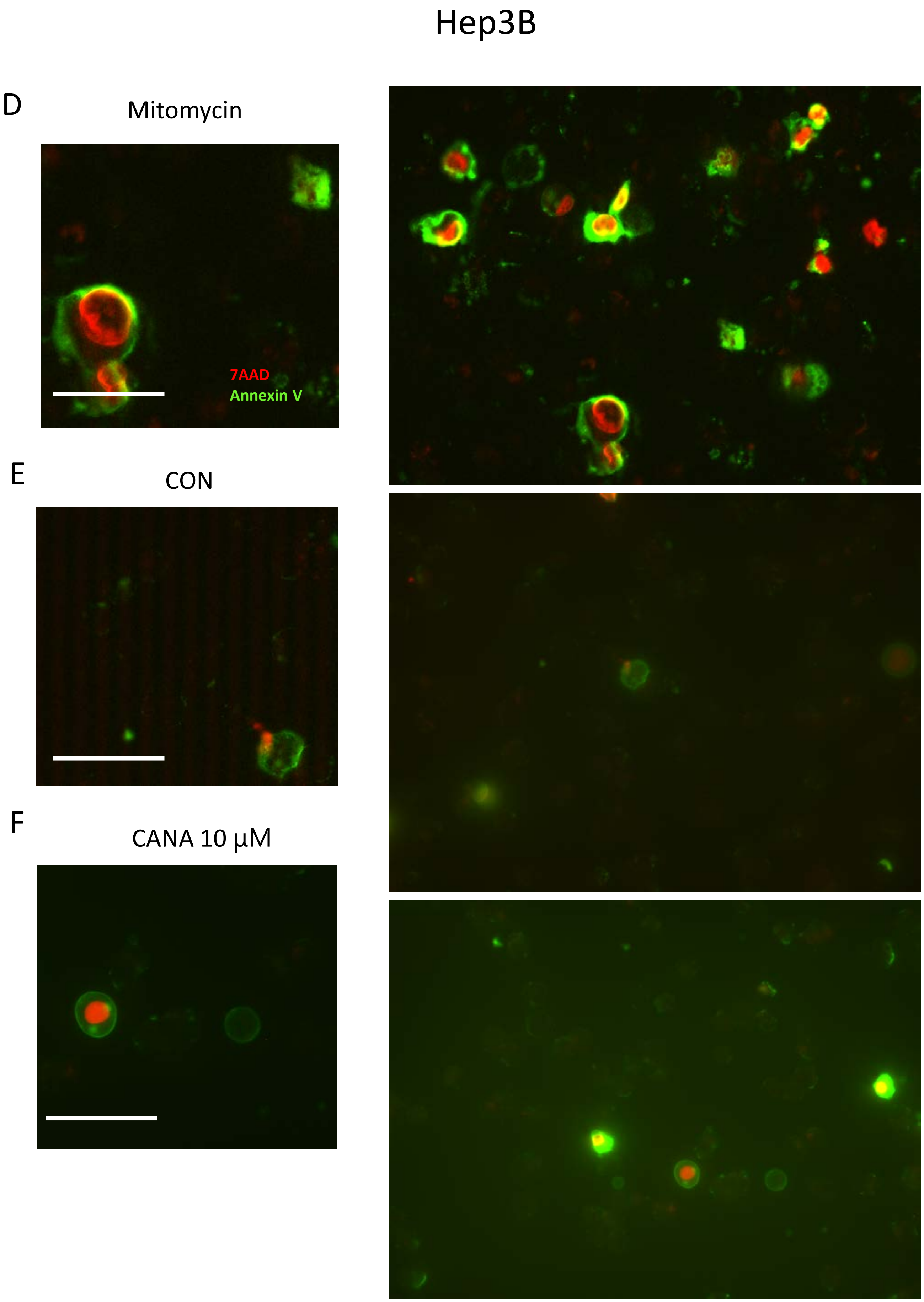

Figure 6

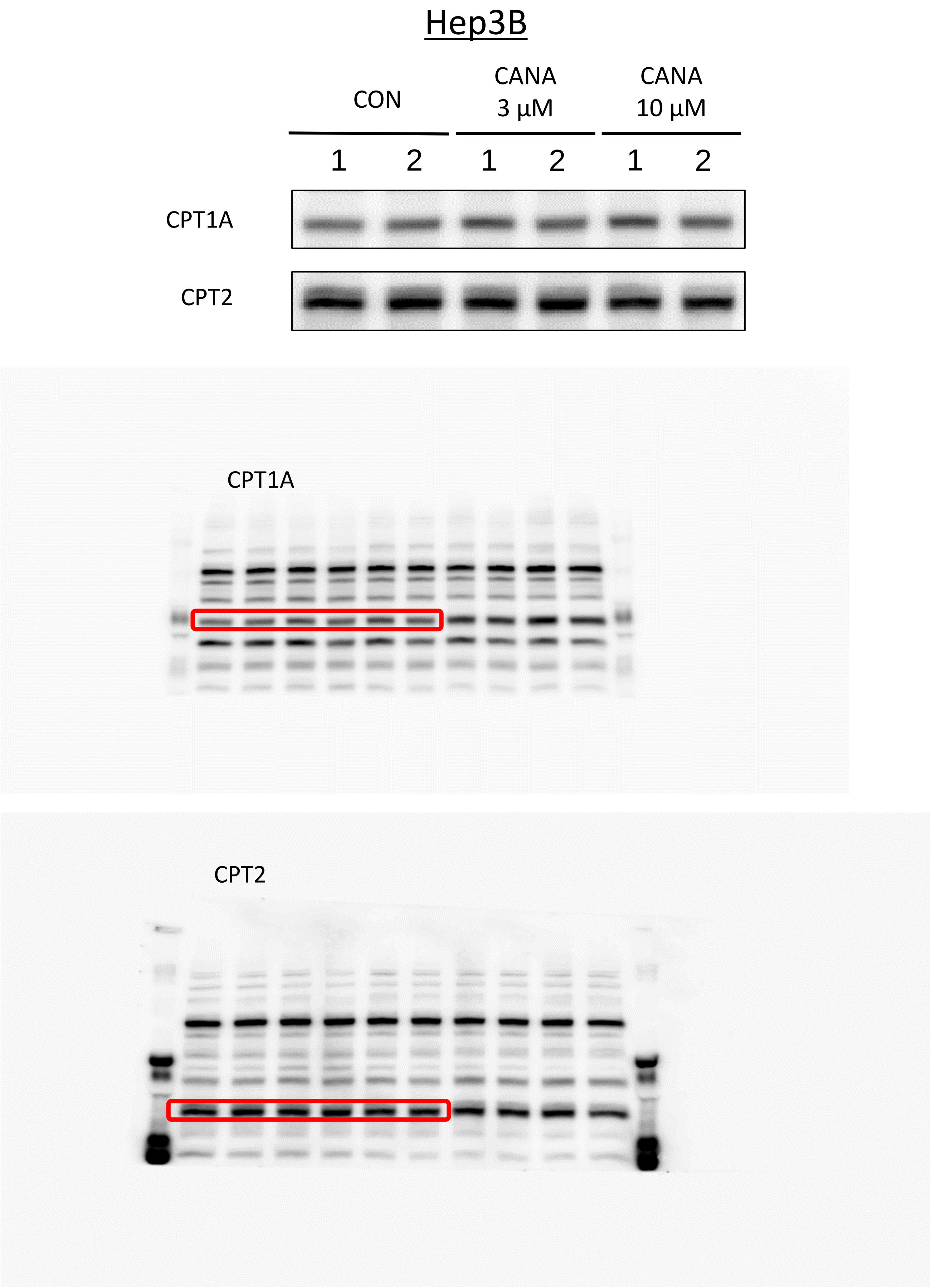

Figure 6

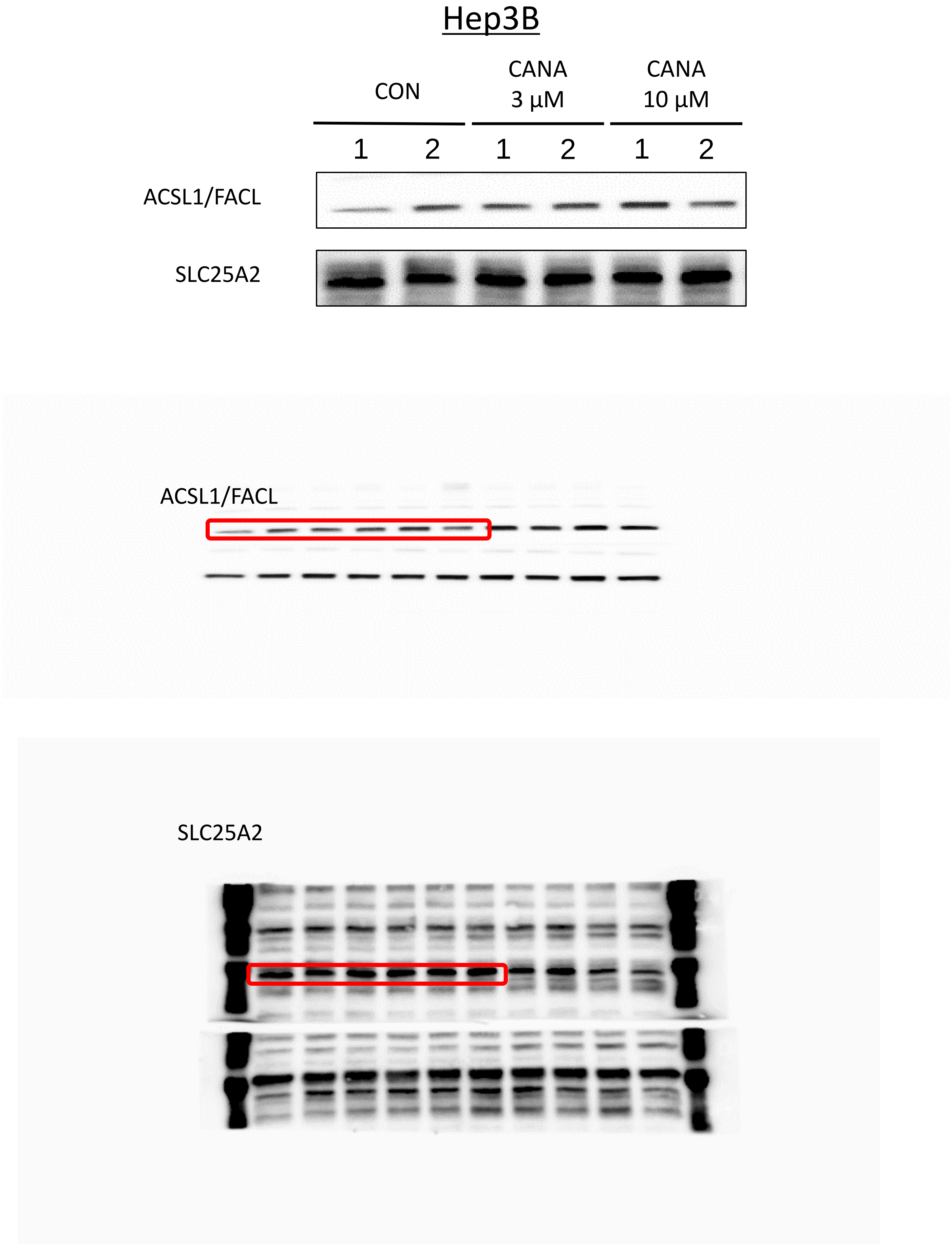

Hep3B
CON
CANA 3 μM
CANA 10 μM
1 2 1 2 1 2
ACSL1/FACL
SLC25A2
ACSL1/FACL
SLC25A2

Figure 6

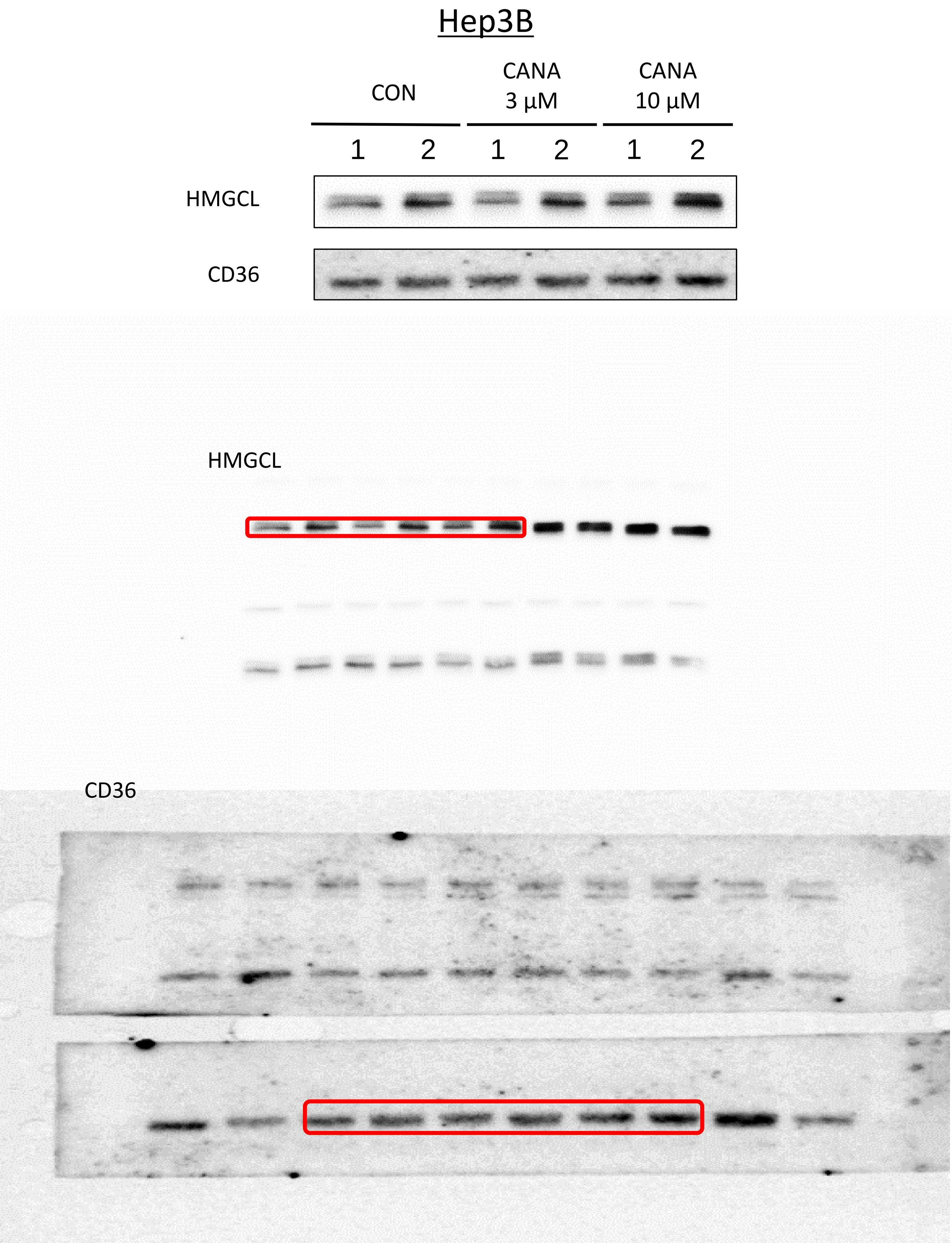

Figure 6

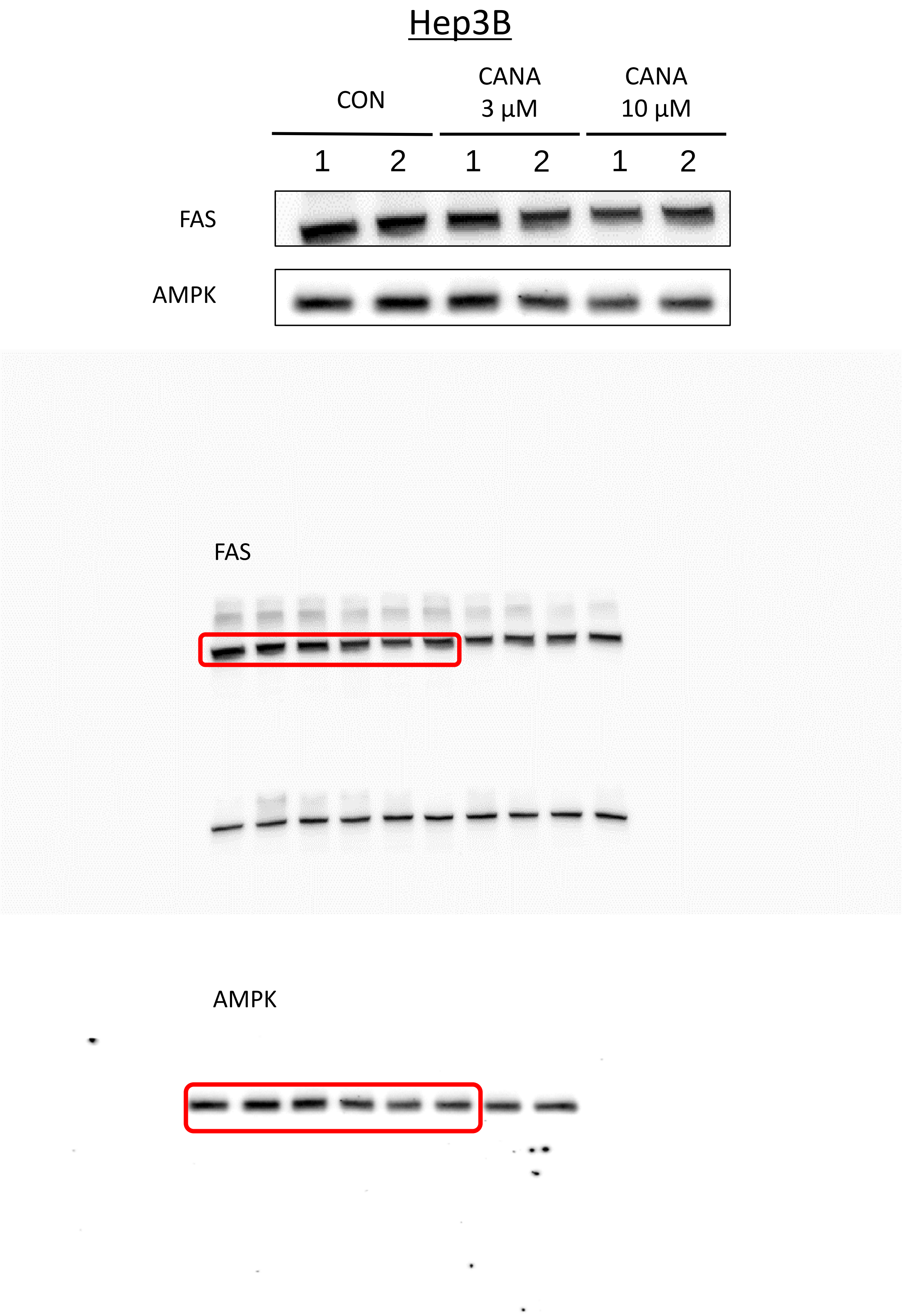

Figure 6

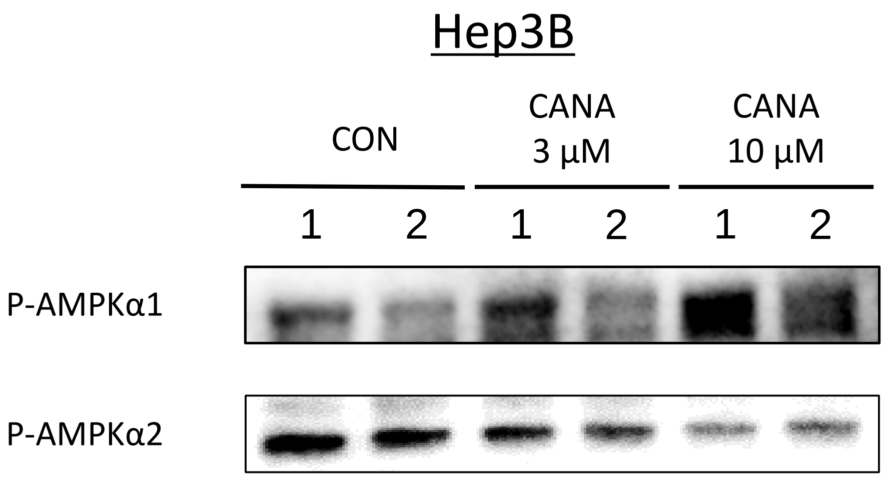
Hep3B
CON
CANA
3 µM
CANA
10 µM
1
2
1
2
1
2
P-AMPKα1
P-AMPKα2

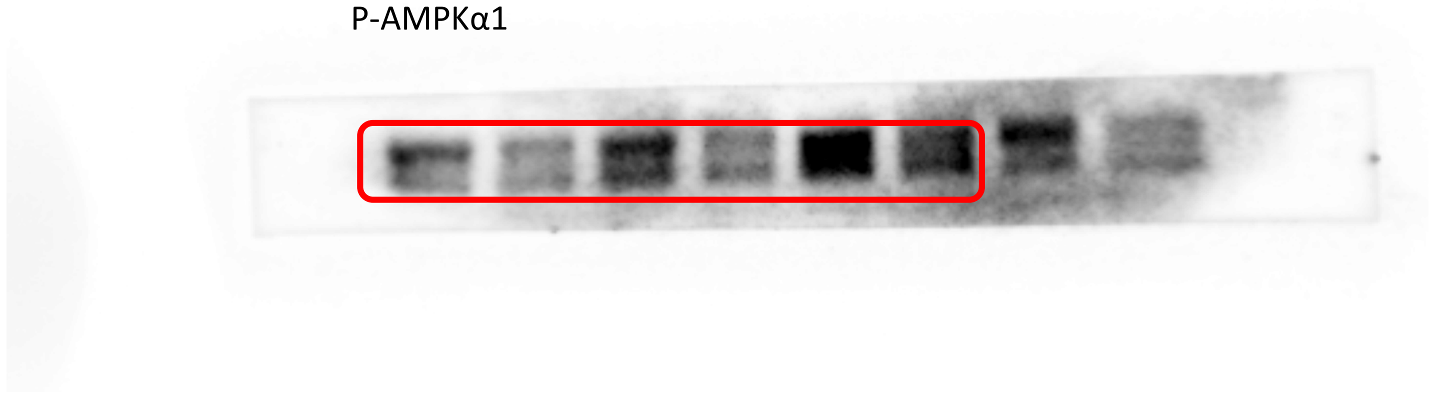
P-AMPKα1

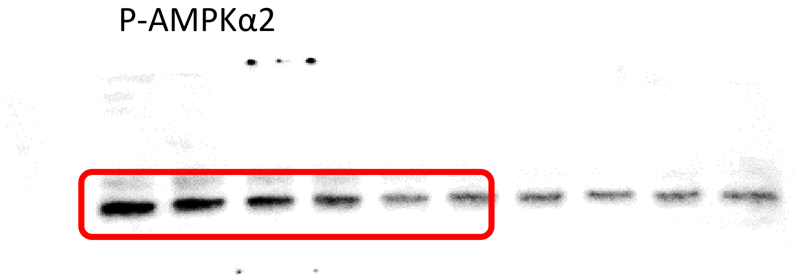
P-AMPKα2

Supplementary figure 1

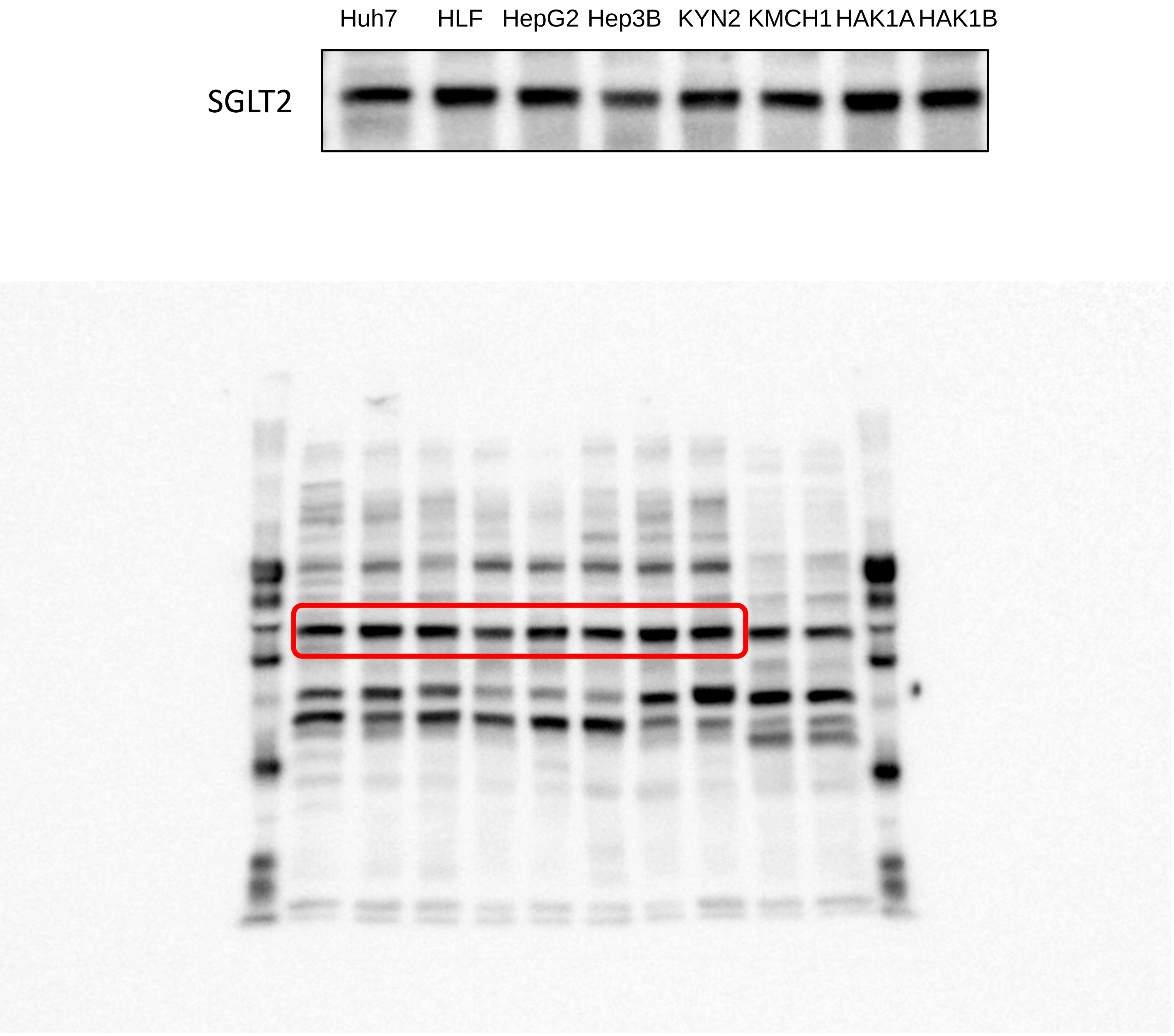

Supplementary figure 2

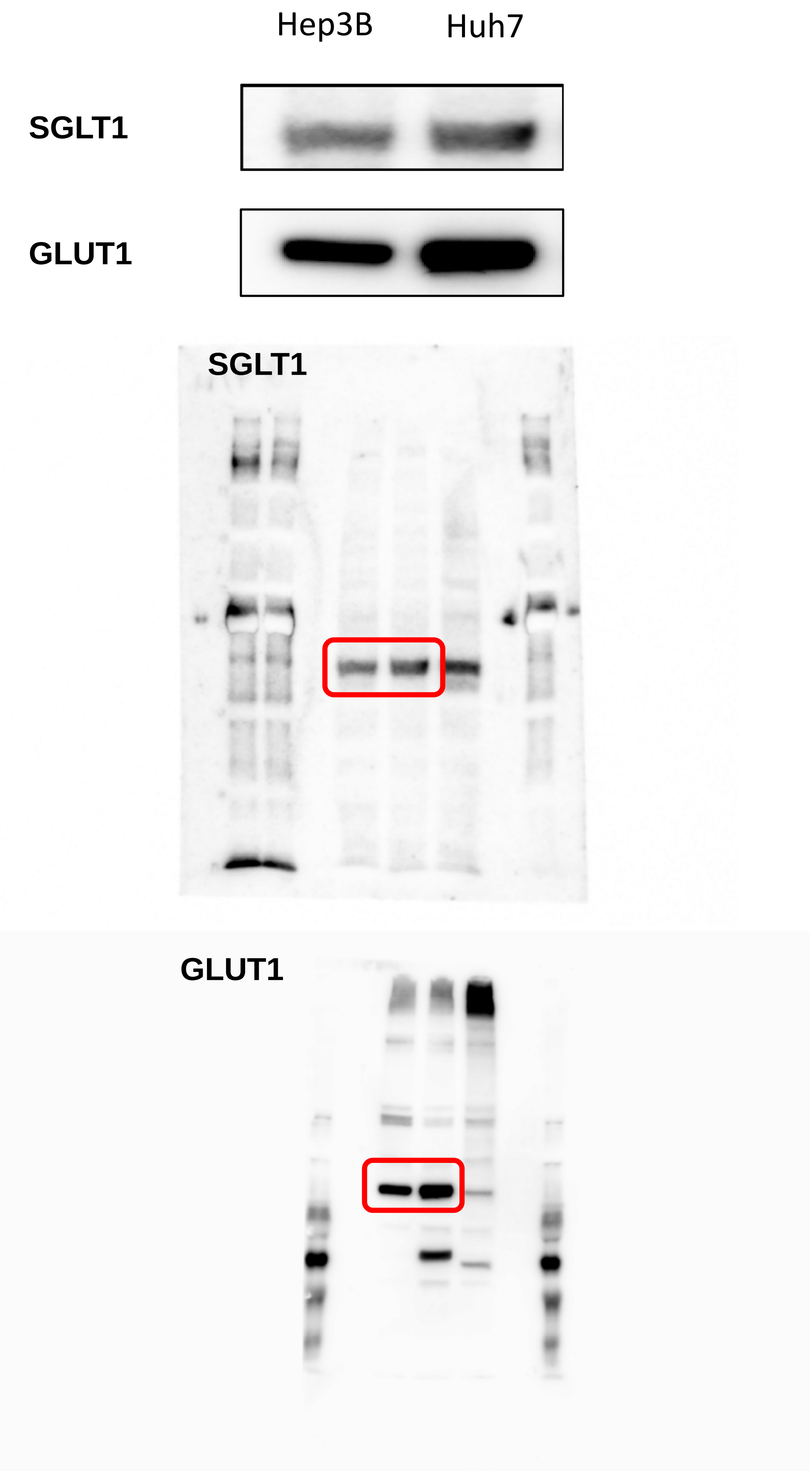

Supplementary figure 2

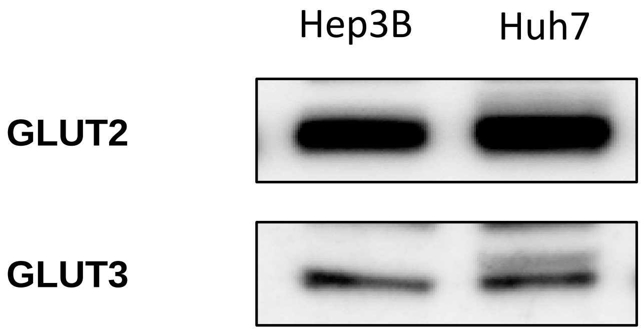

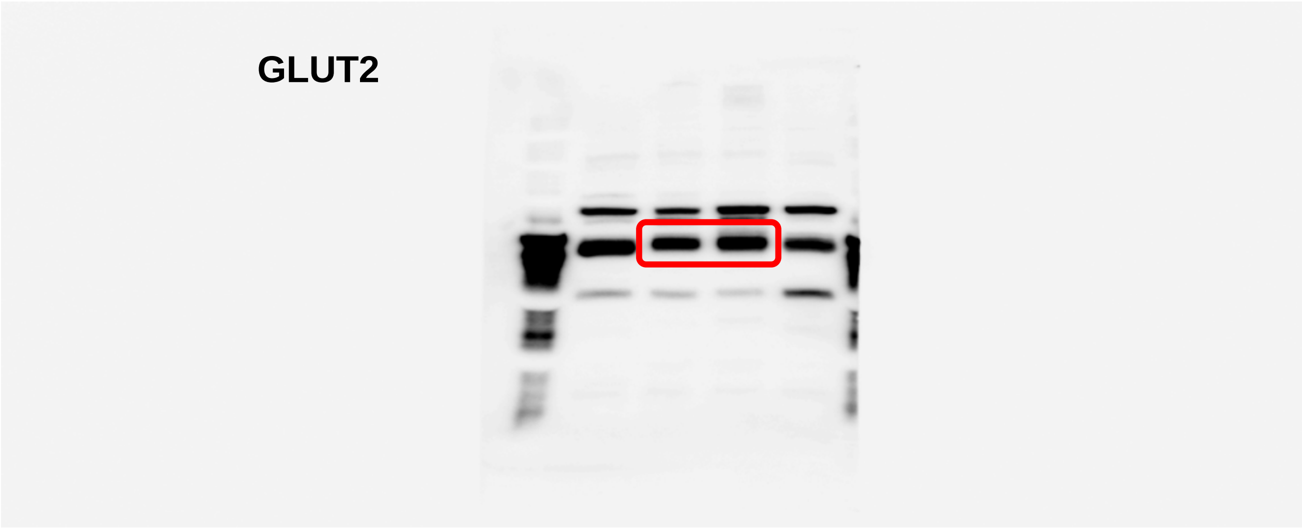

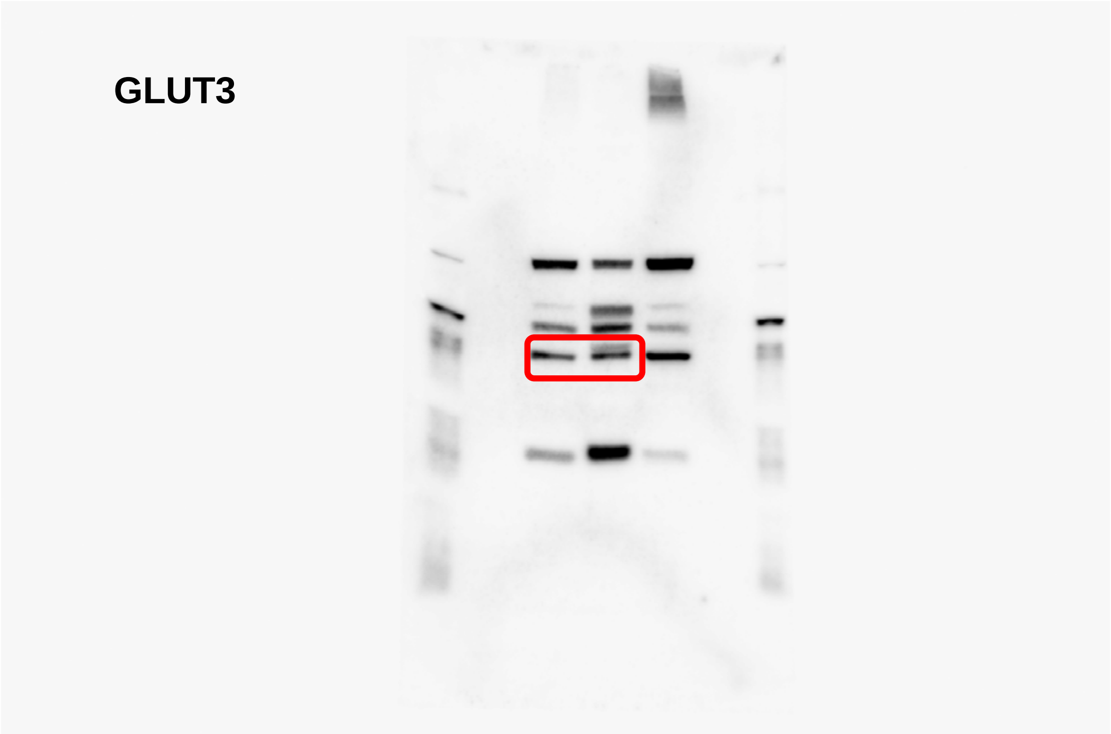

Supplementary figure 2

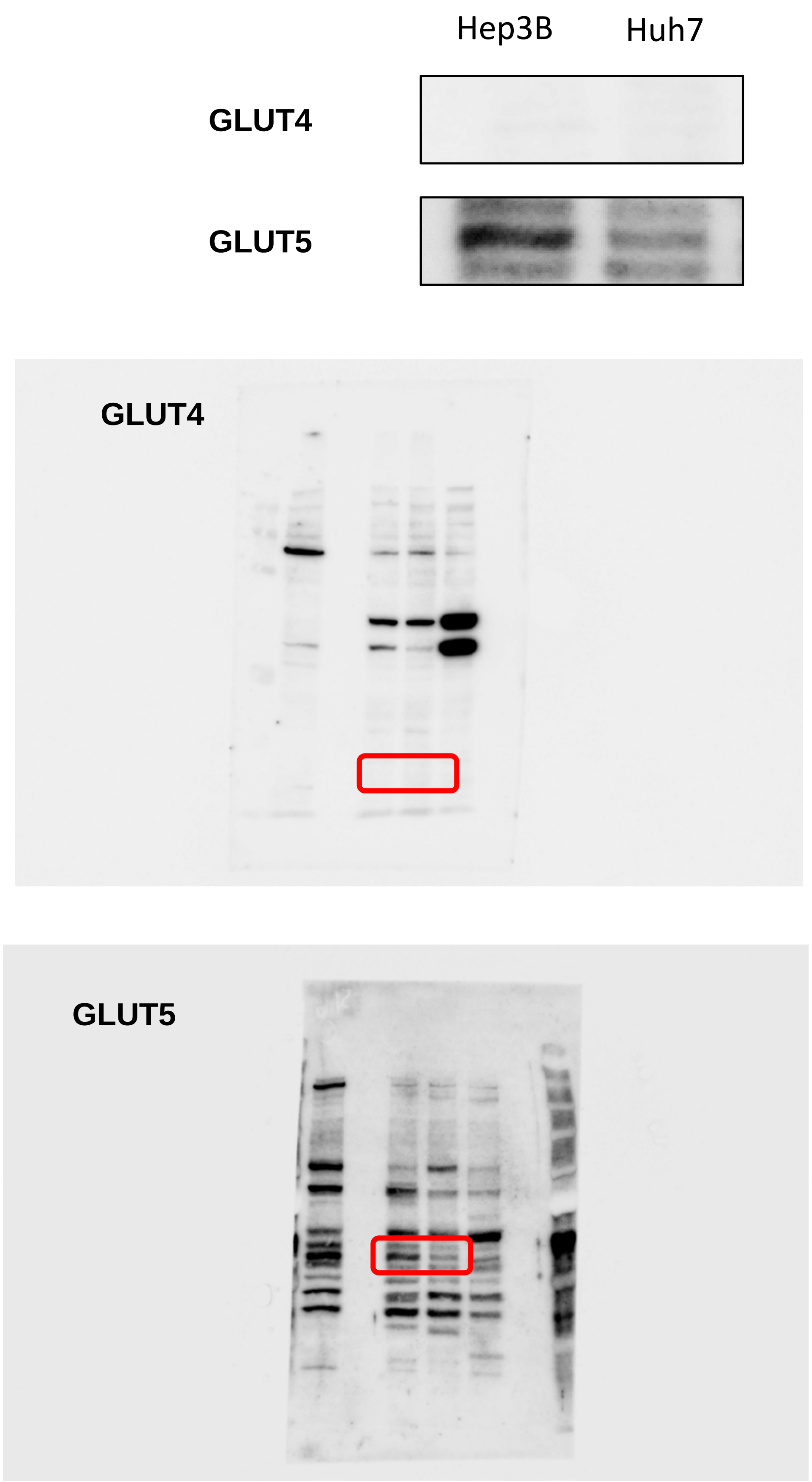

Supplementary figure 2

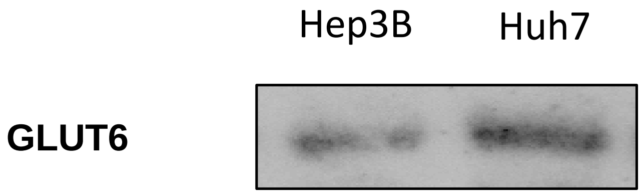

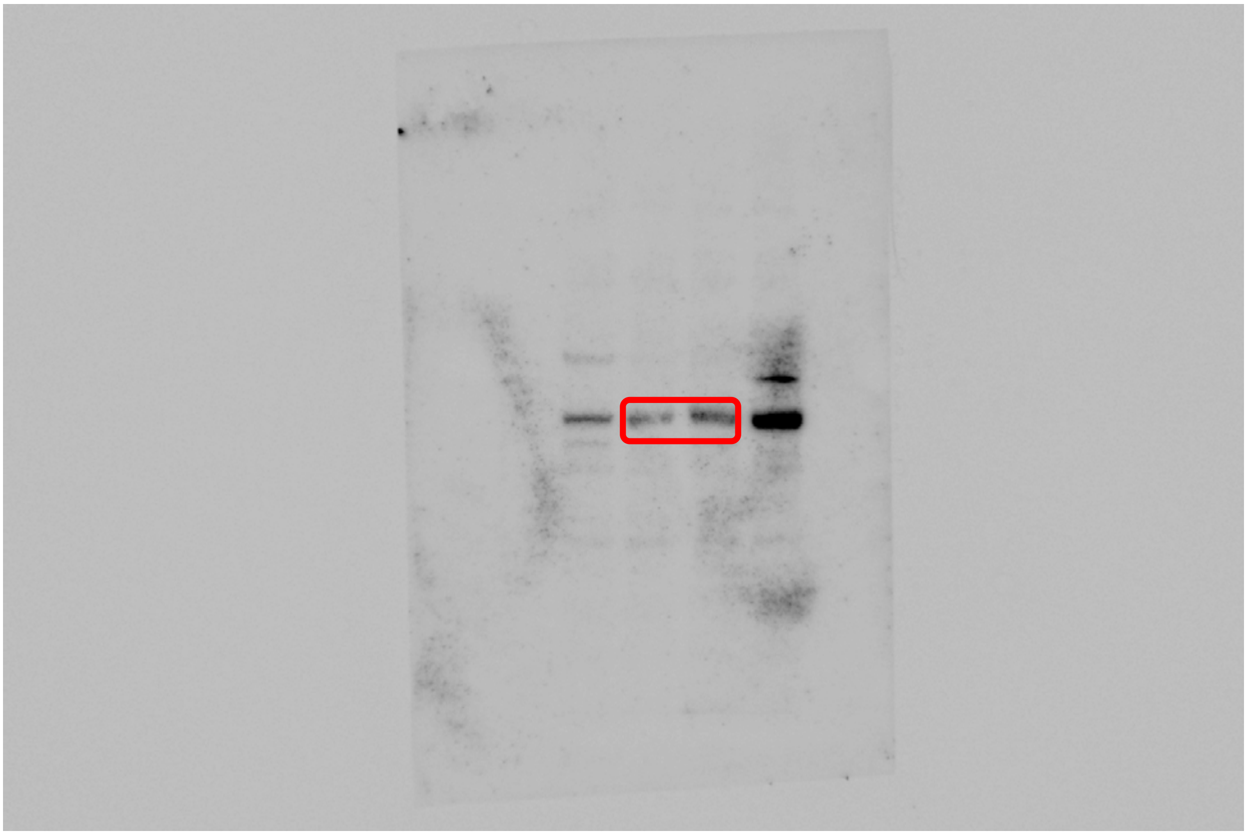

Supplementary figure 3

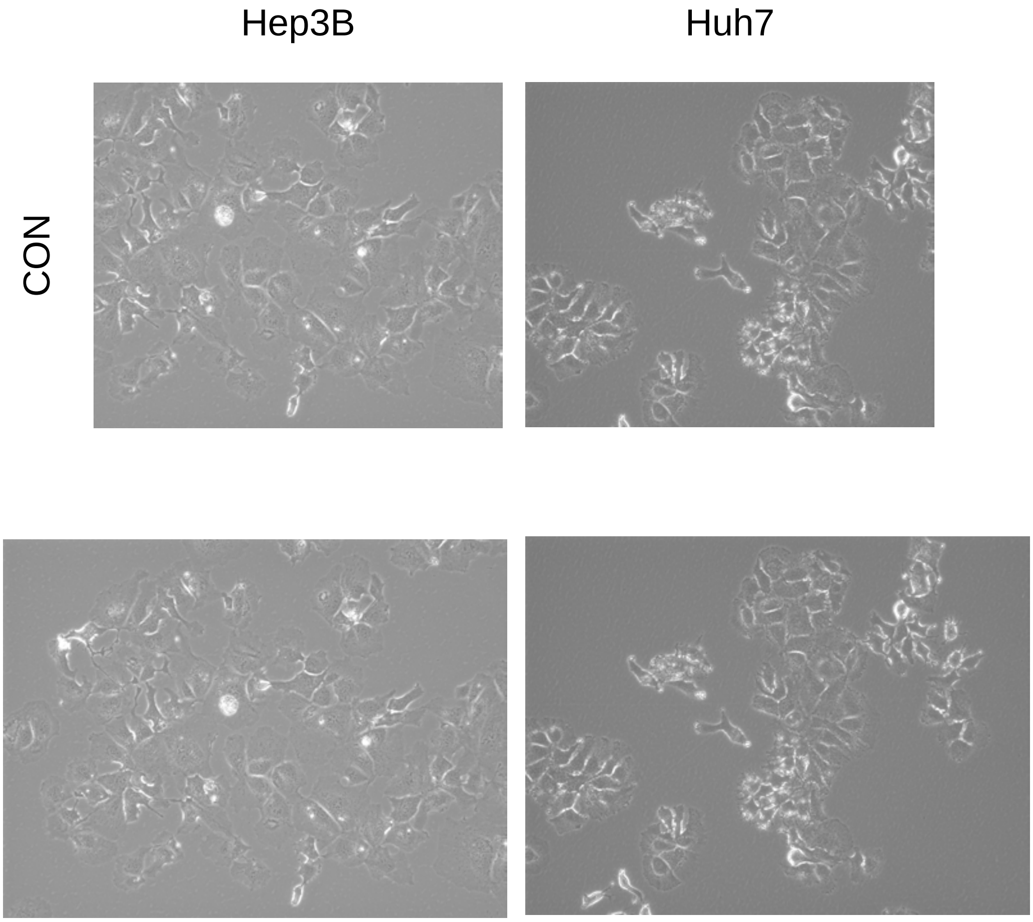

Supplementary figure 3

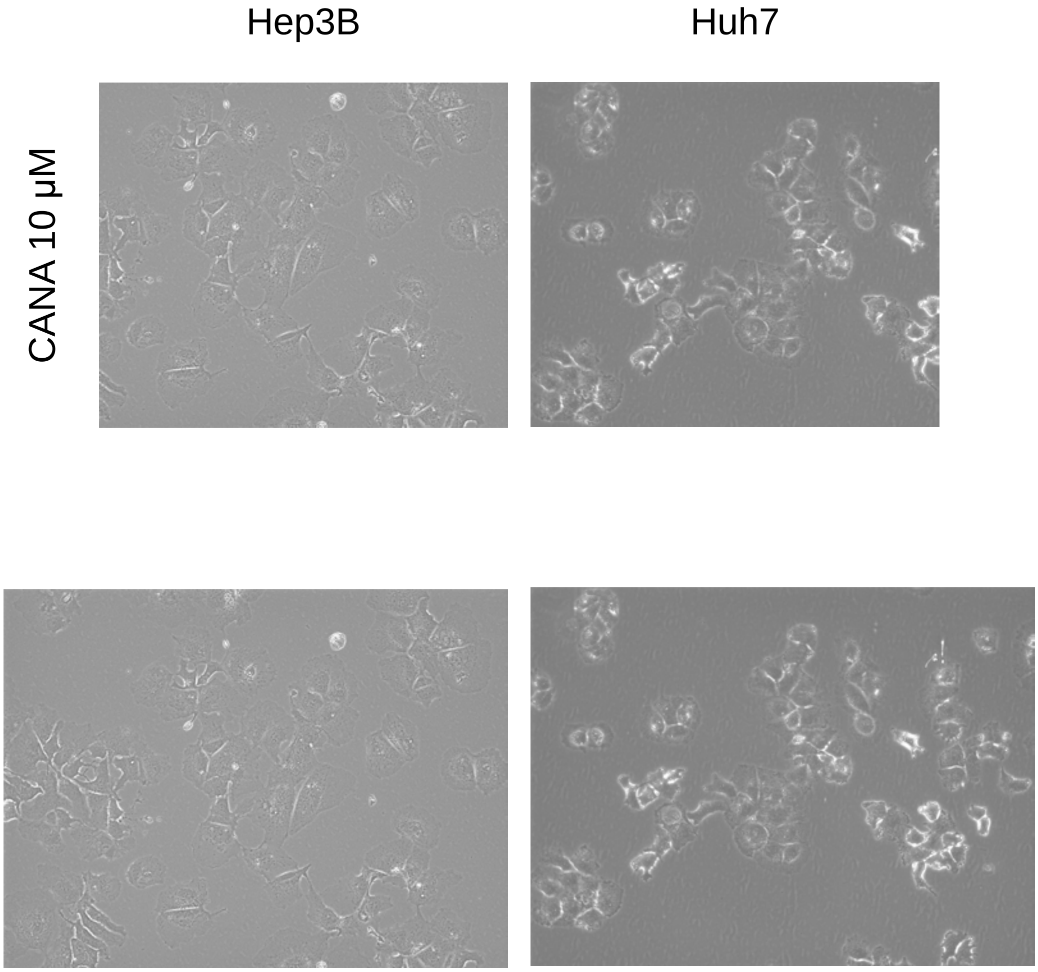

Supplementary figure 3

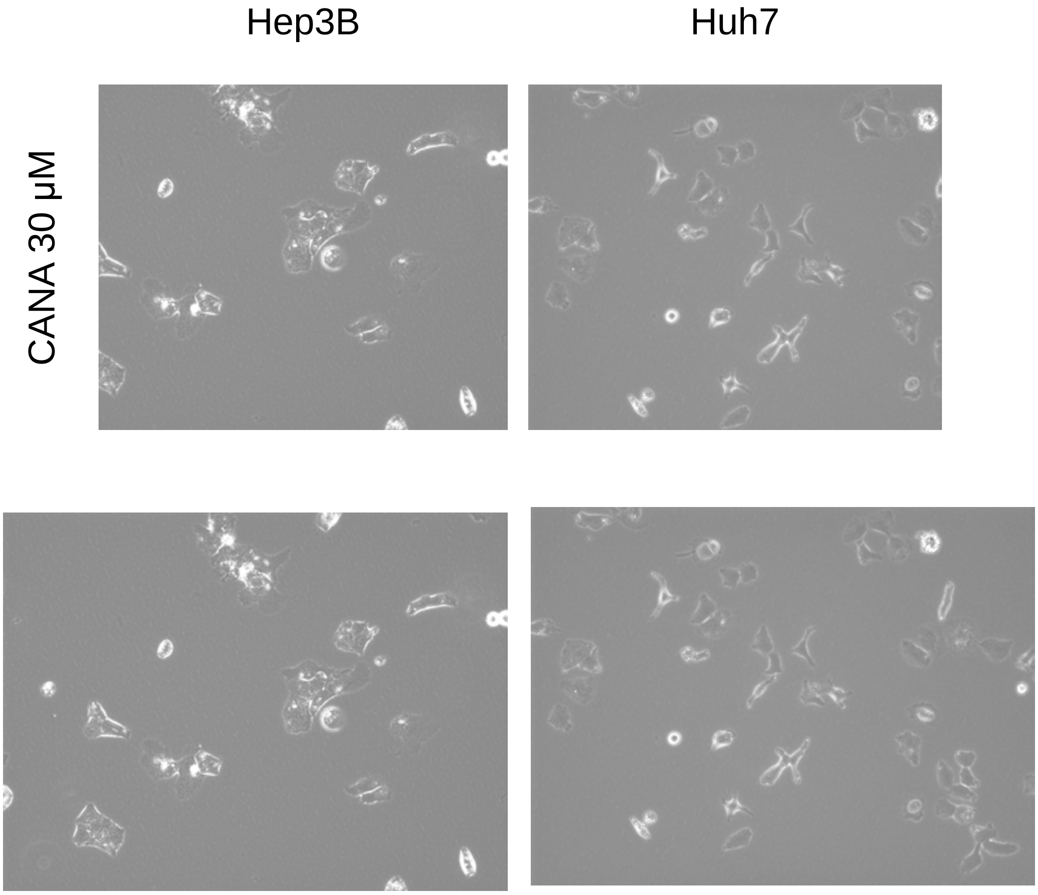

Supplementary figure 4A

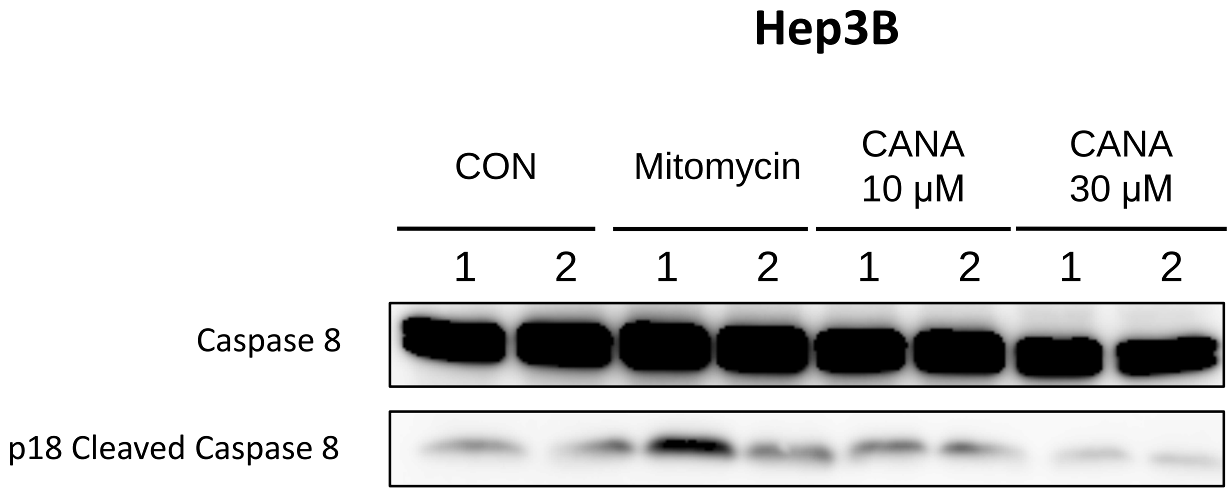

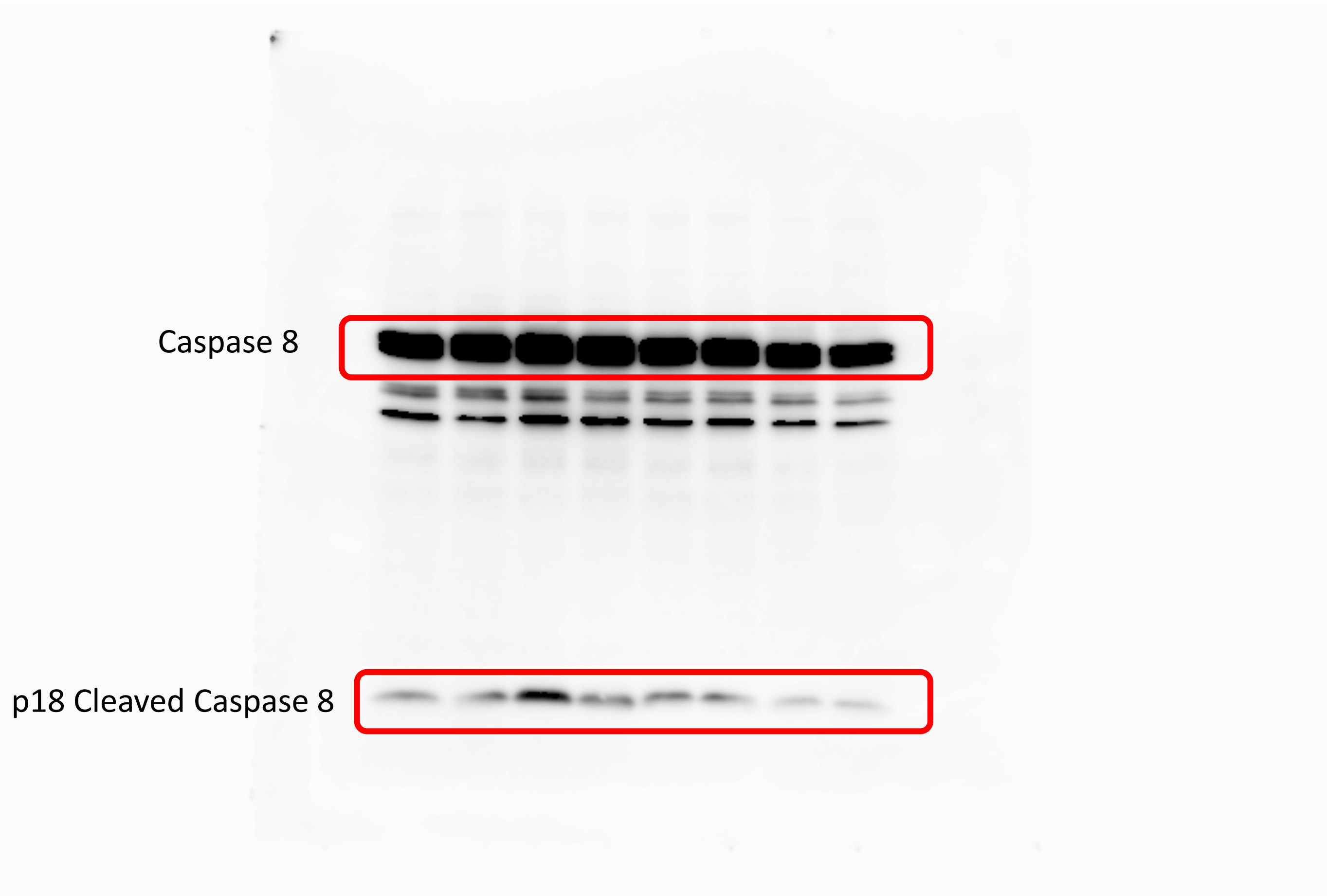

Supplementary figure 4A

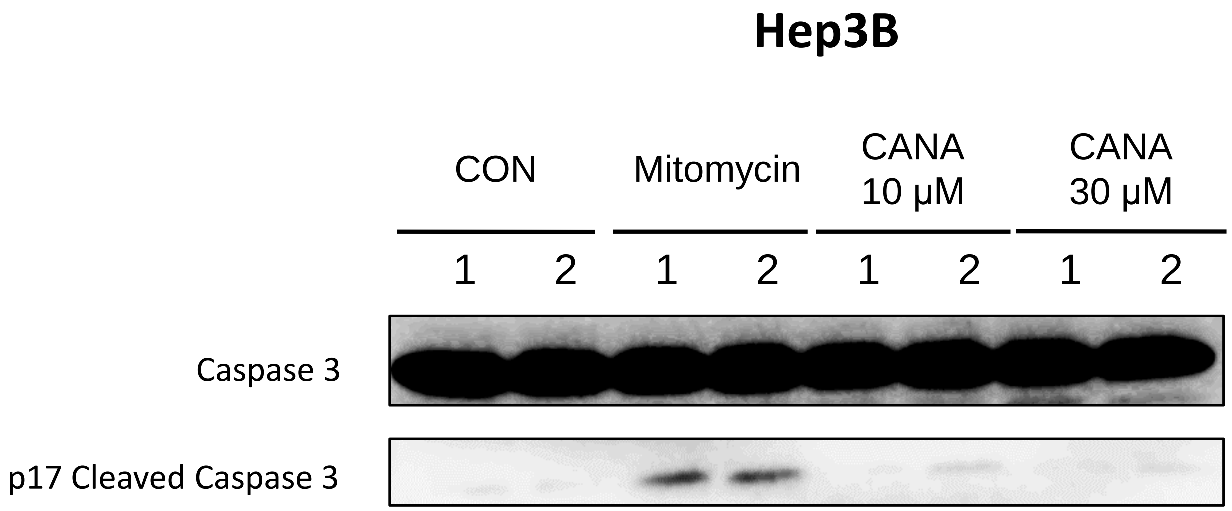

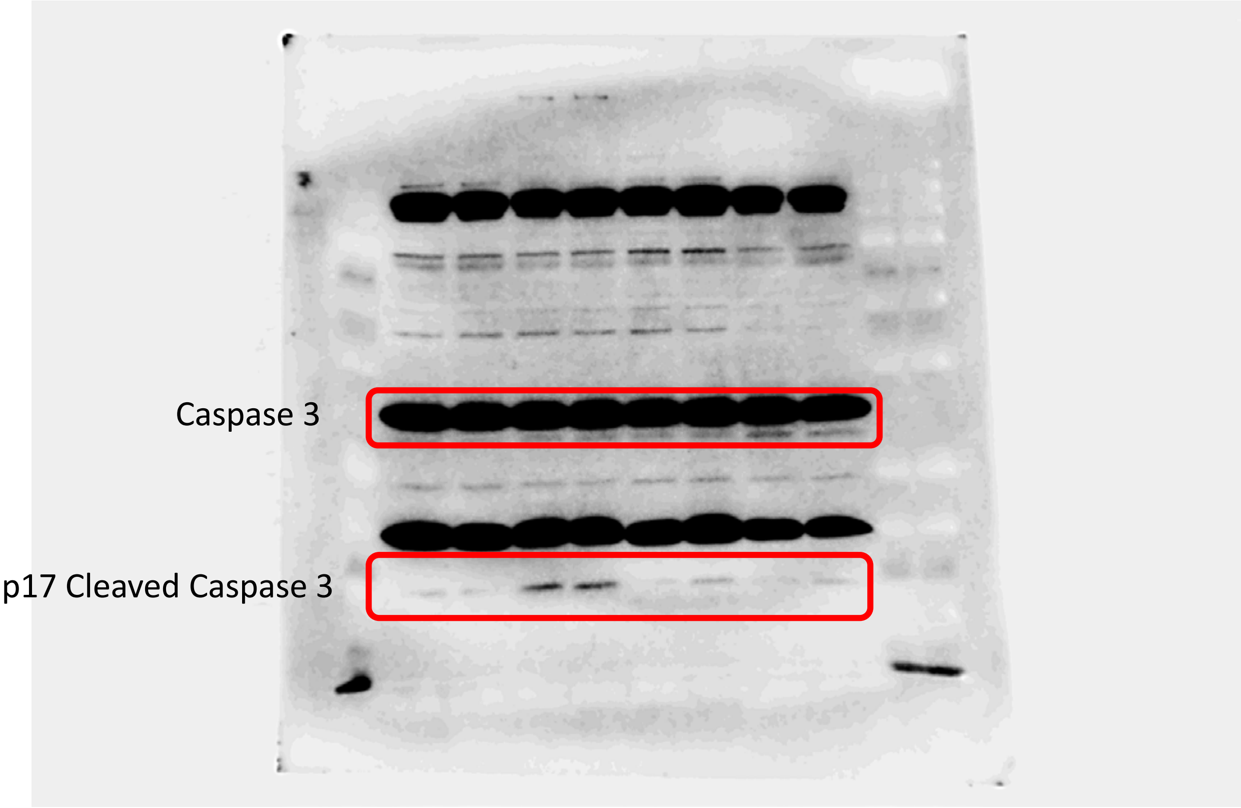

Supplementary figure 4A

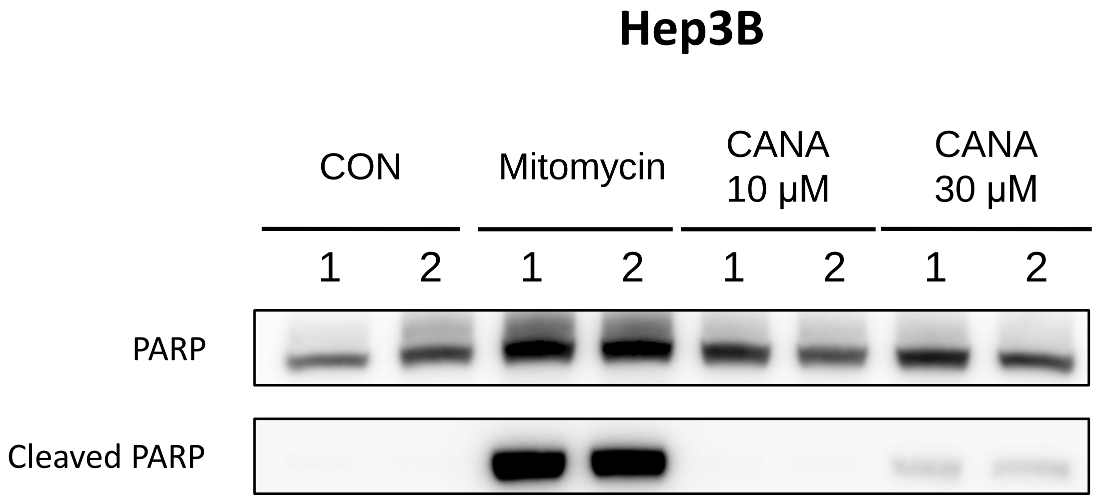

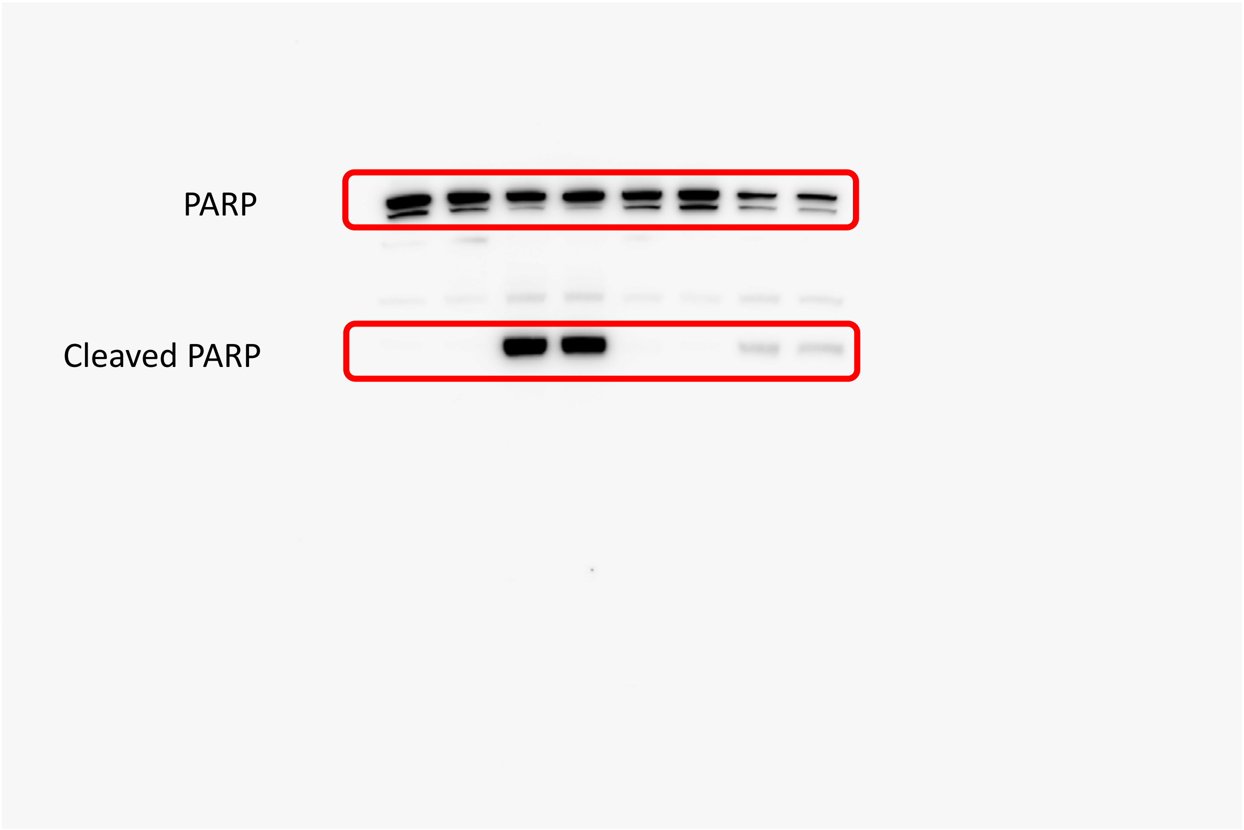

Supplementary figure 4B

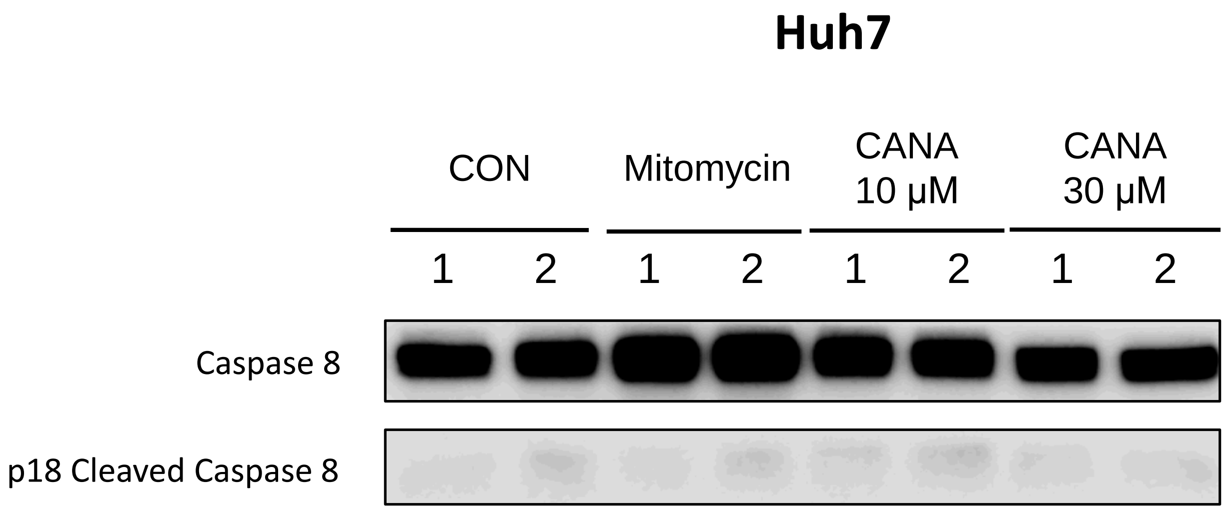

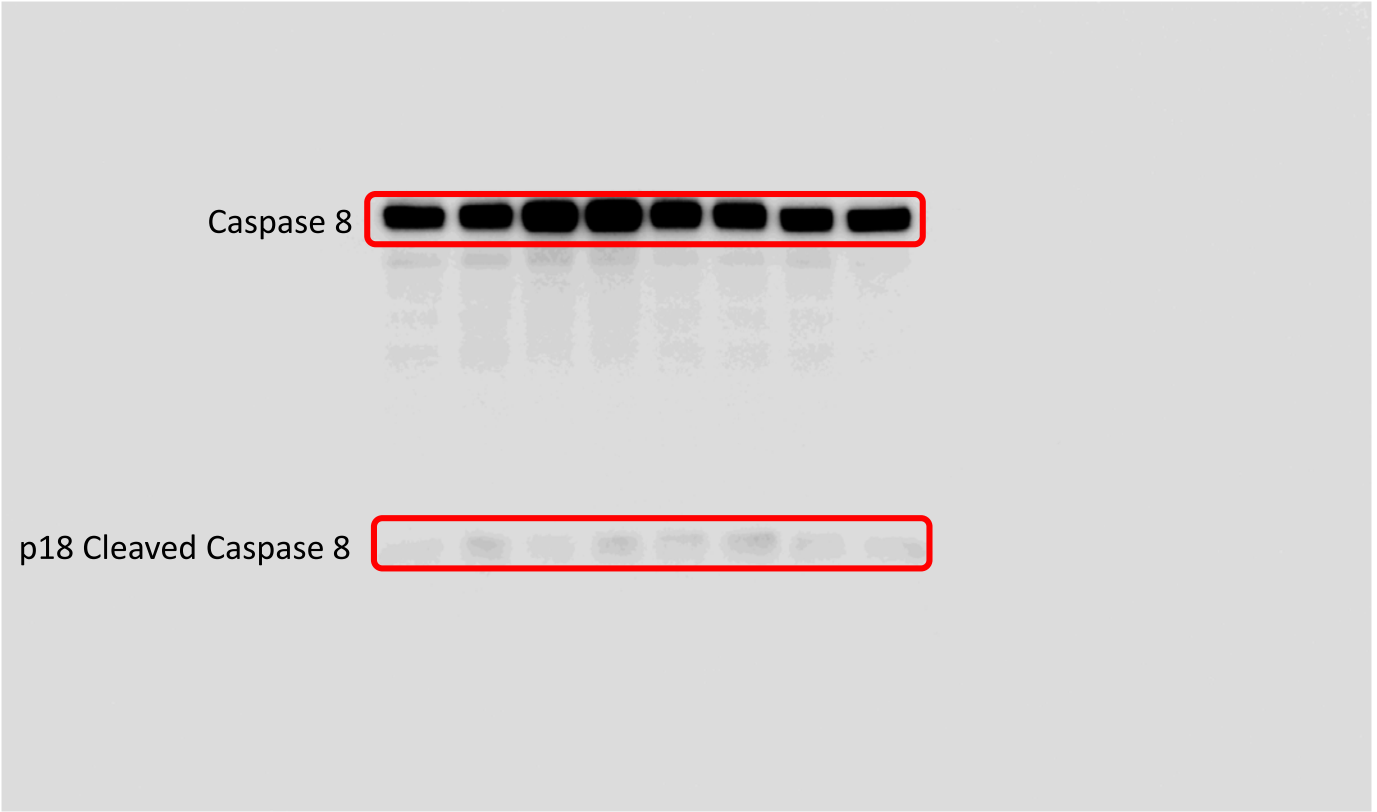

Supplementary figure 4B

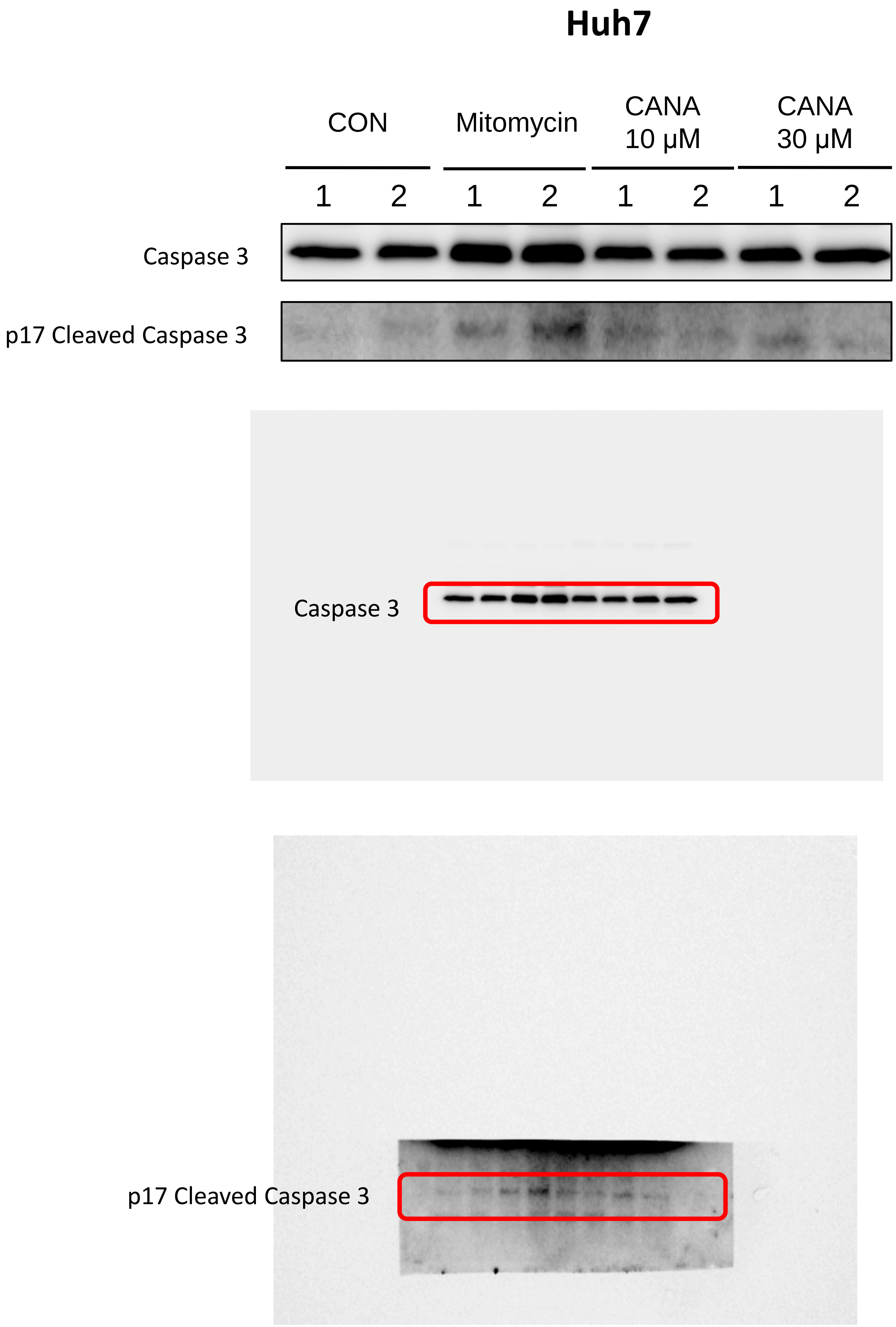

Supplementary figure 4B

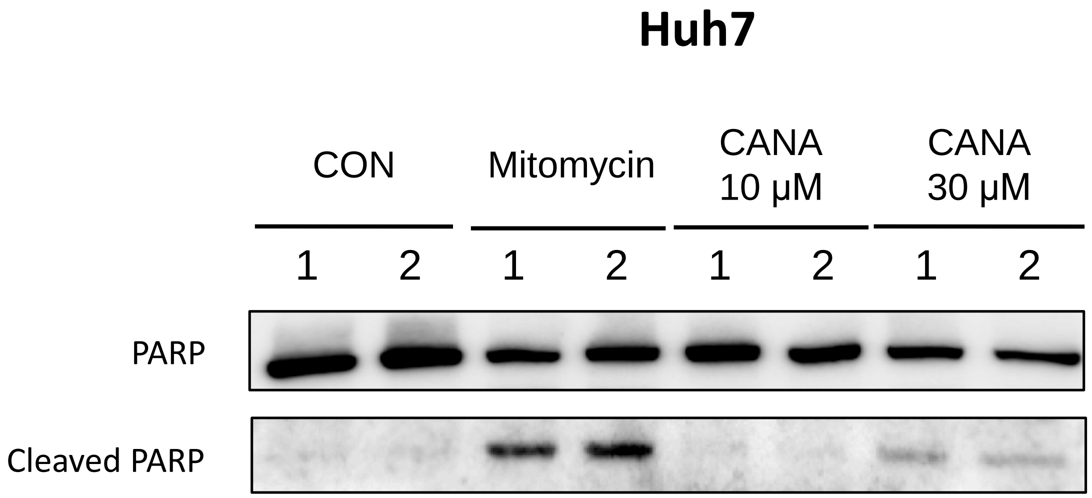

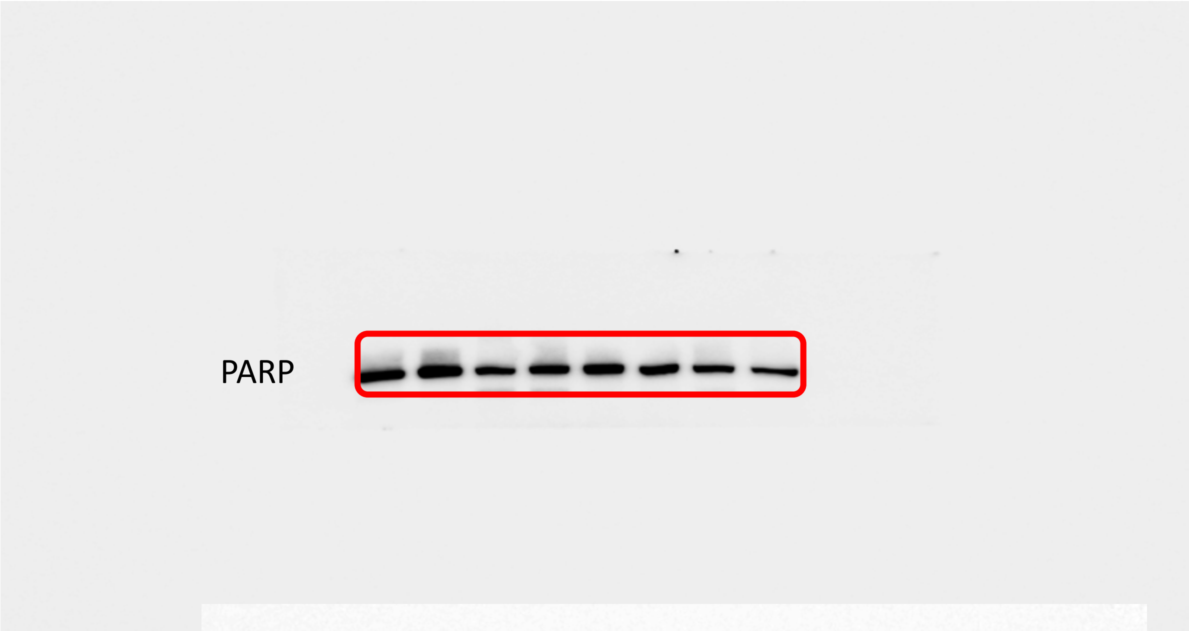

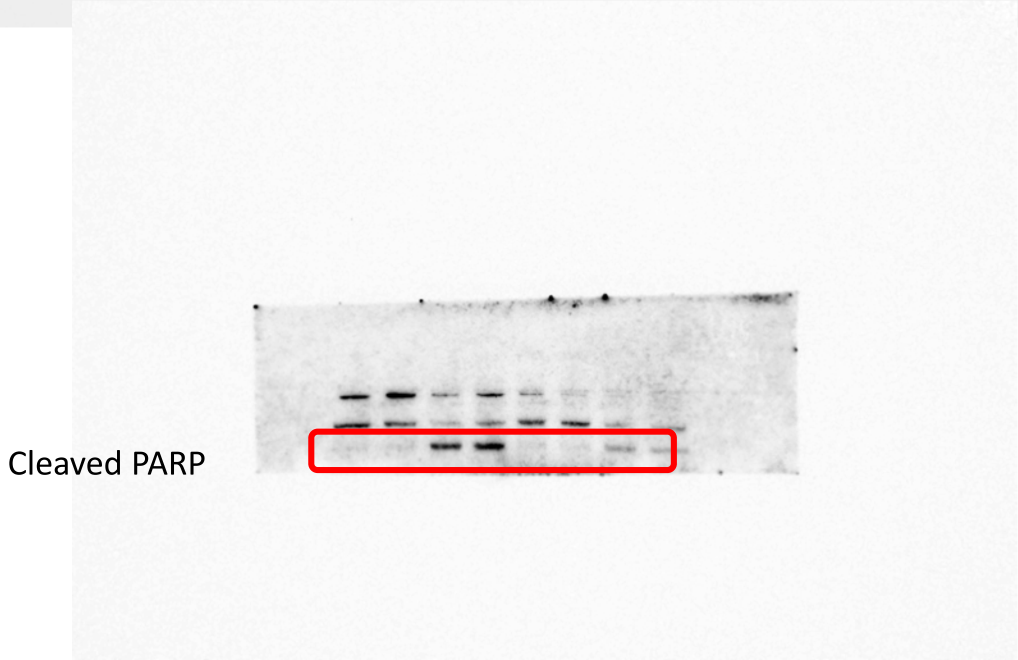

Supplementary figure 5

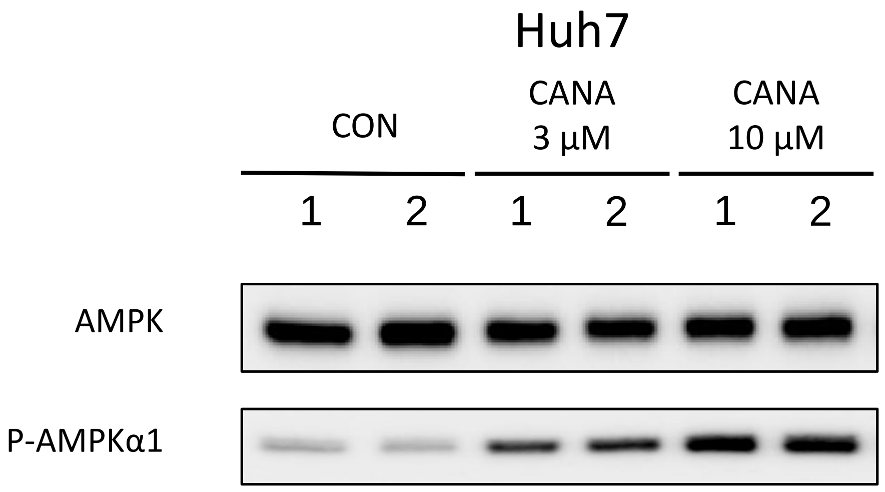

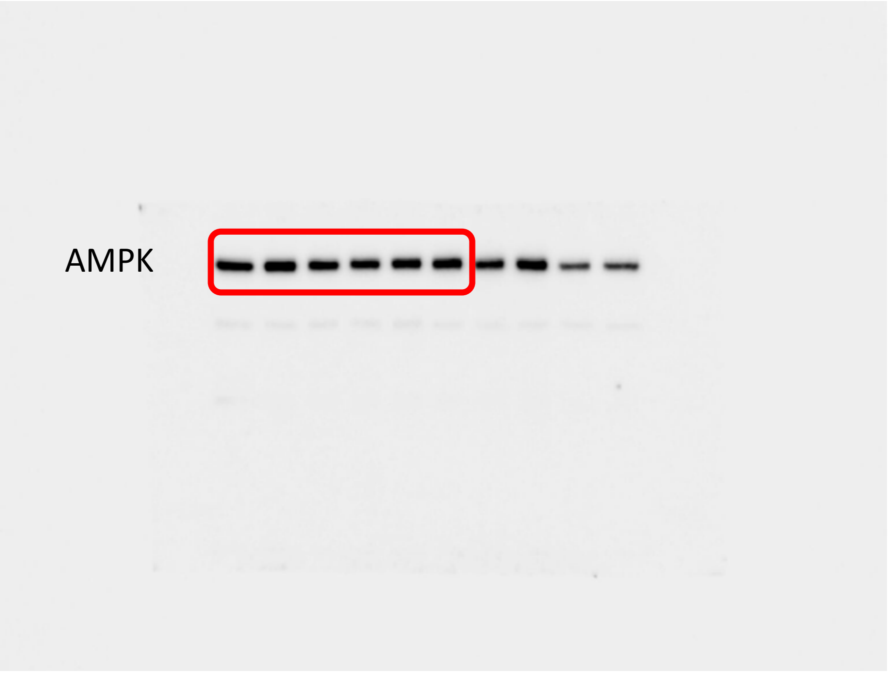

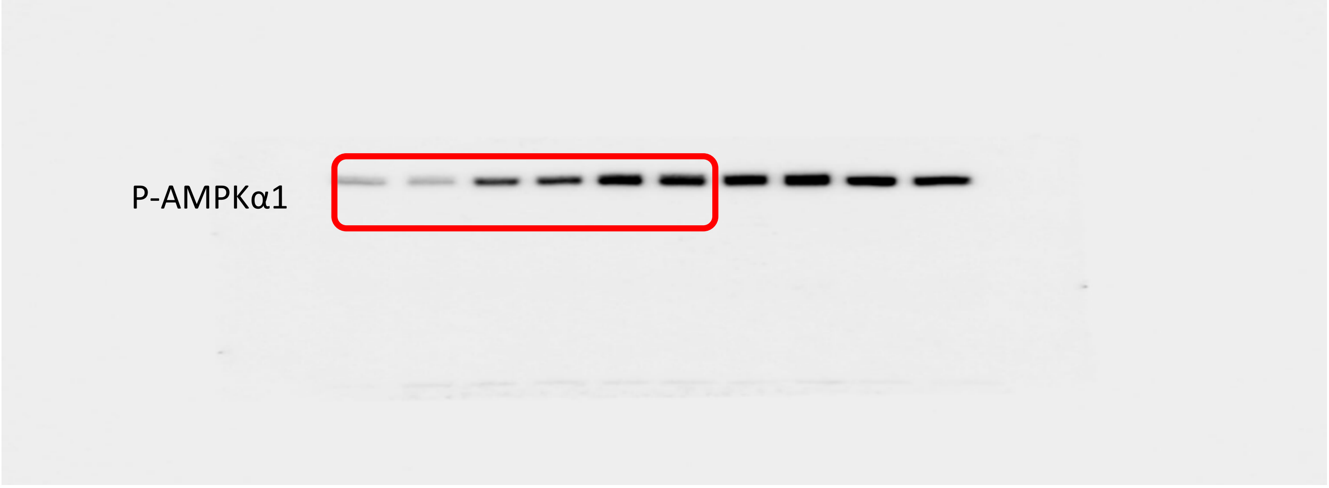

Supplementary figure 5

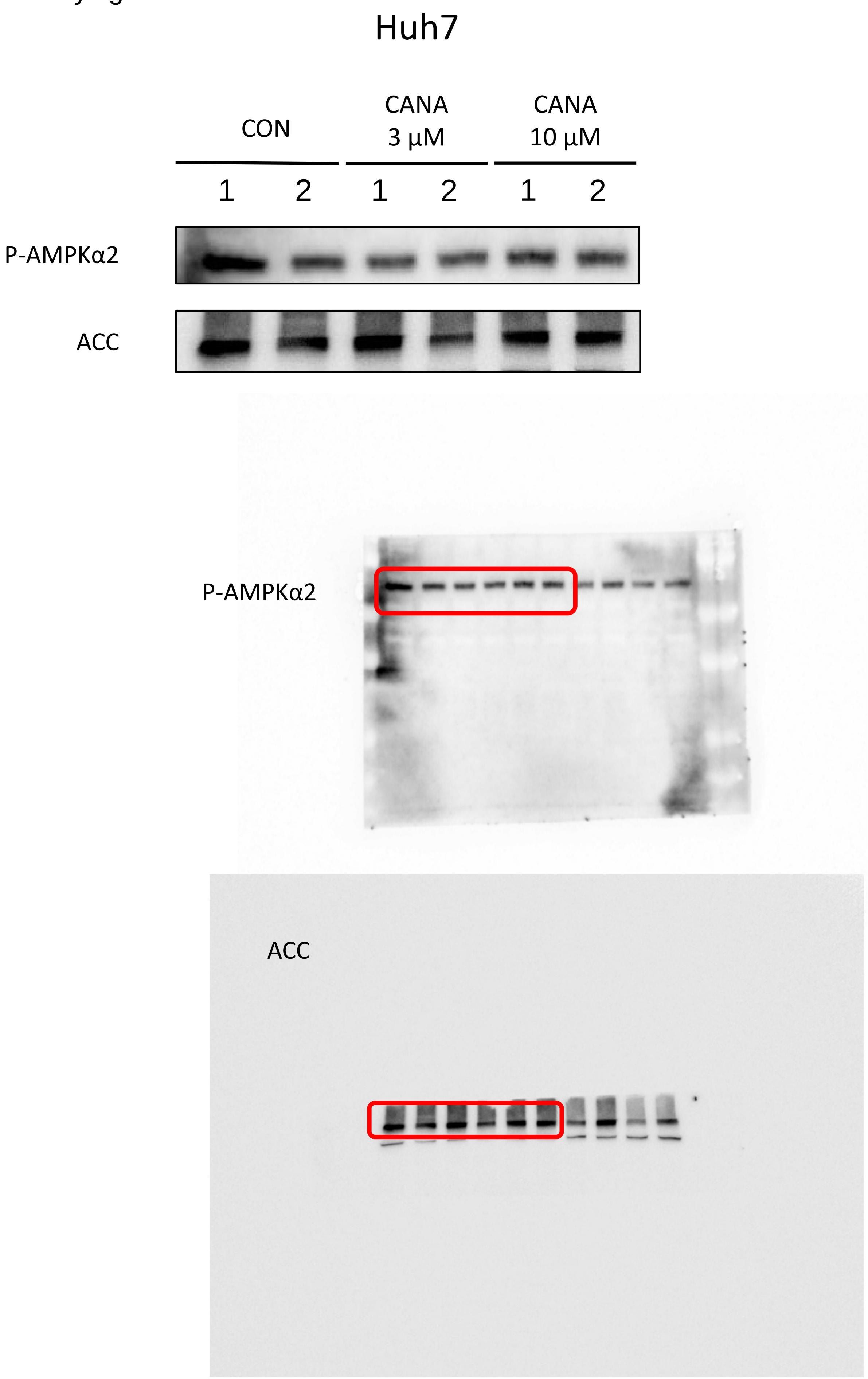

Supplementary figure 5

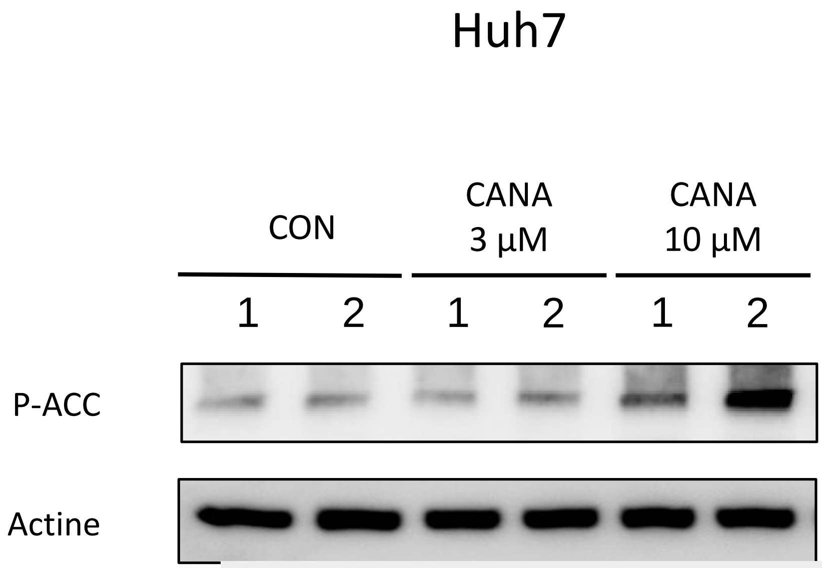

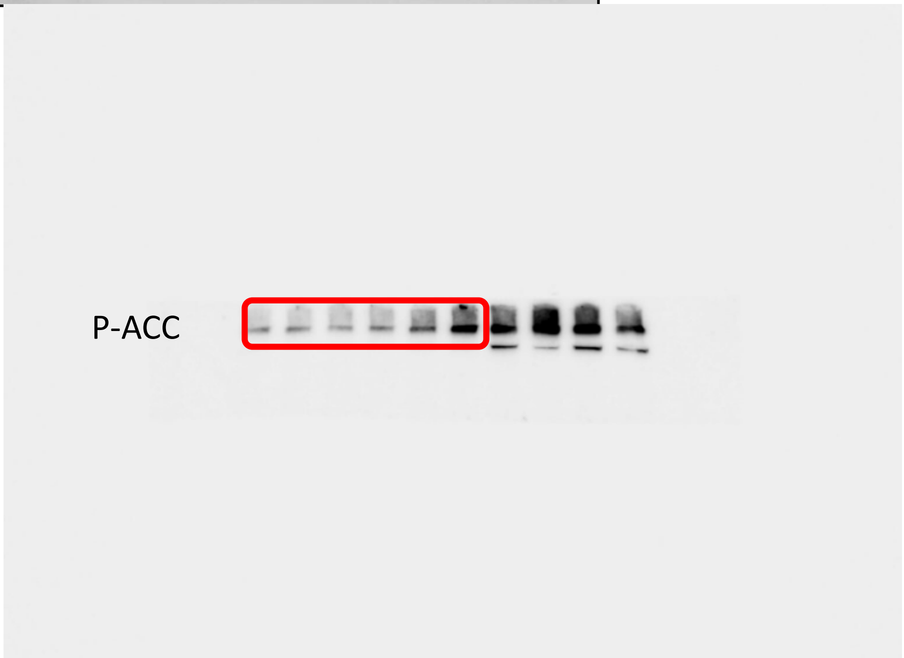

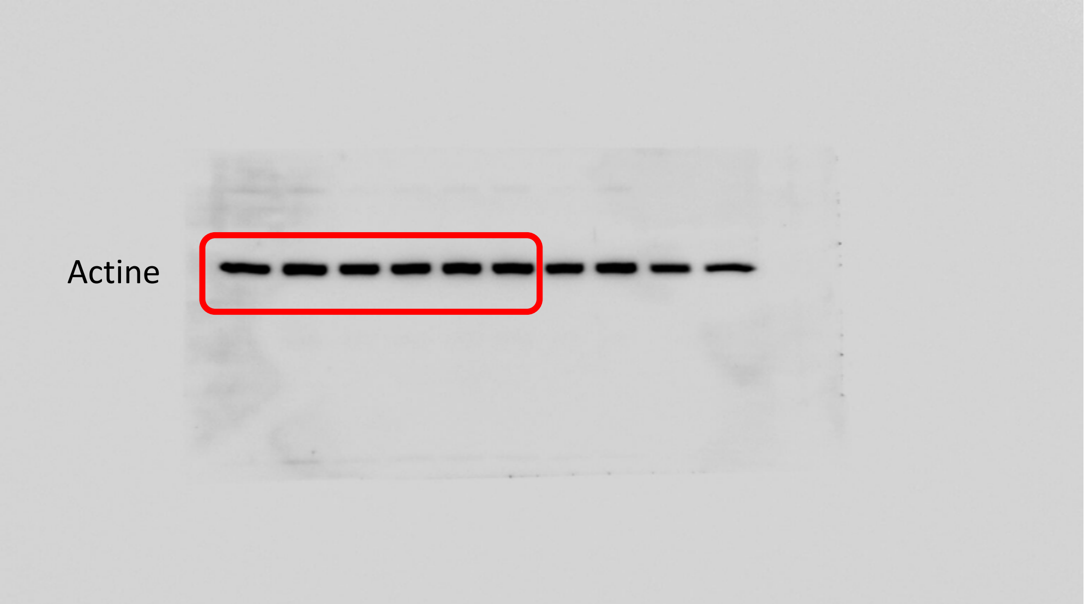

Supplementary figure 5

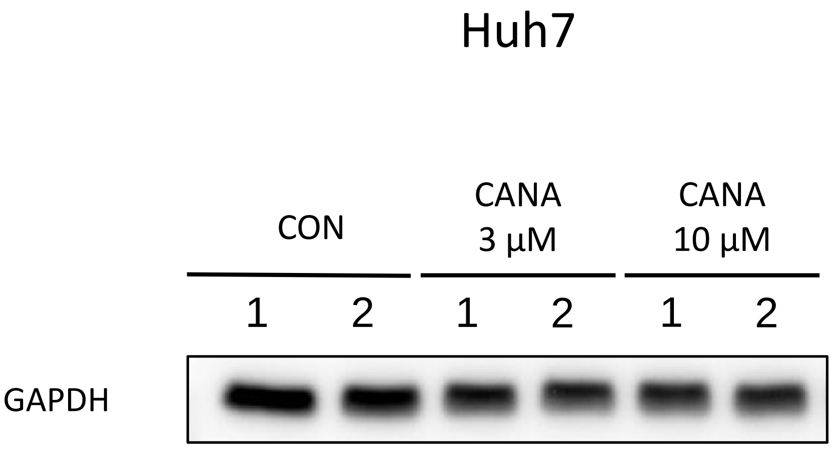

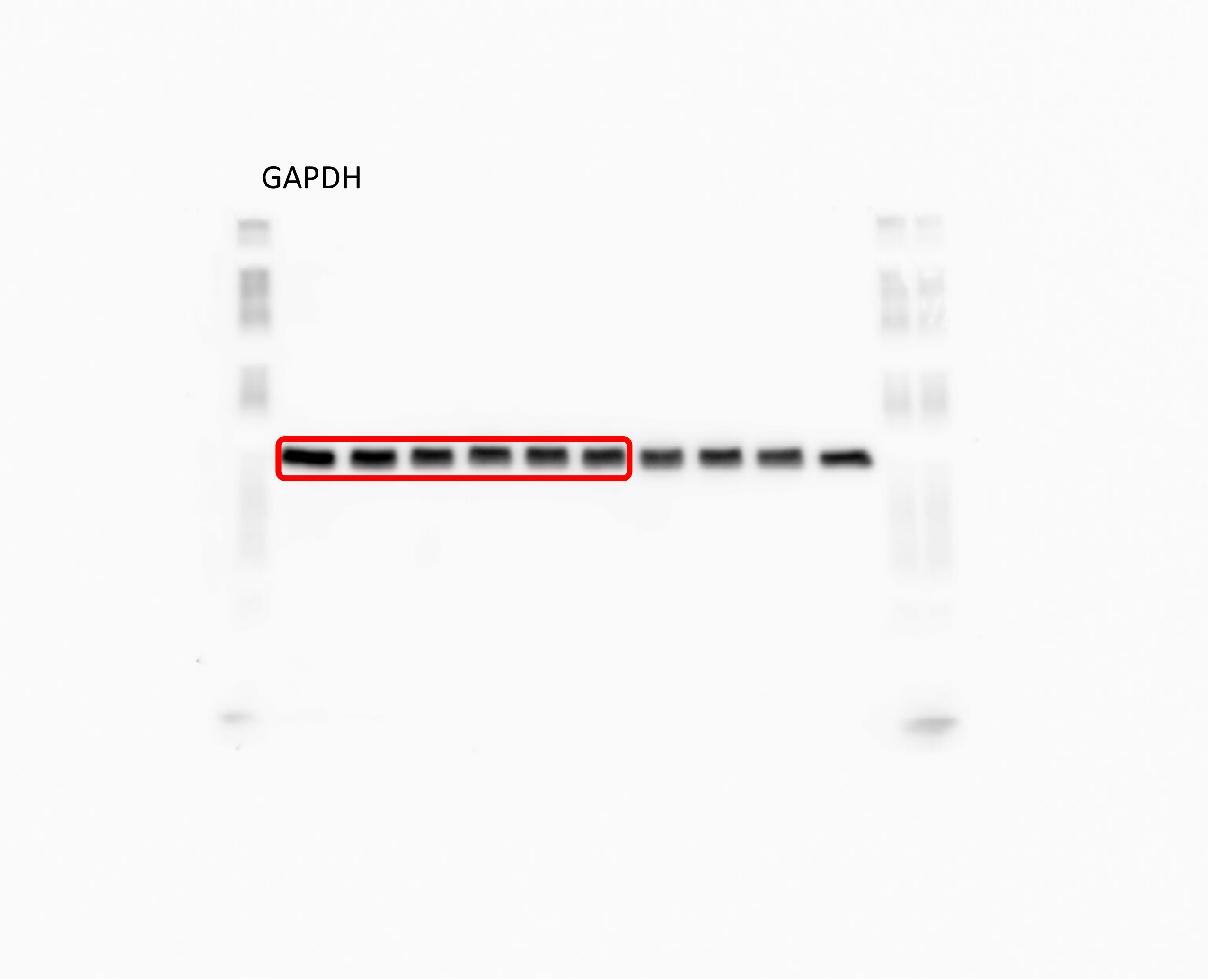

Supplement: S1 Raw image — (PDF) [file pone.0232283.s009.pdf]
