# Supplementary material for: Effects of canagliflozin on growth and metabolic reprograming in hepatocellular carcinoma cells: Multi-omics analysis of metabolomics and absolute quantification proteomics (iMPAQT)
Source: PLoS One. 2020 Apr 28;15(4):e0232283. doi: 10.1371/journal.pone.0232283 (PMC7188283; doi:10.1371/journal.pone.0232283)
Supplement: S1 Table — (DOCX) [file pone.0232283.s010.docx]

Supplementary Table 1. Effects of CANA on levels of 225 metabolites by metabolomics in Hep3B cells

| Metabolites | Metabolism | Control | |  | SGLT2i | | P |
| --- | --- | --- | --- | --- | --- | --- | --- |
|  |  | Mean | SD |  | Mean | SD |  |
| 3,4-Dihydroxyphenylacetate | Phenylalanine Tyrosine metabolism | 472 | 28 |  | 1203 | 72 | >0.0001 |
| Citramalic acid | Valine, leucine and isoleucine metabolism | 5196 | 197 |  | 8322 | 462 | >0.0001 |
| Phosphate | Phosphonate and phosphinate metabolism | 1273 | 65 |  | 1806 | 65 | >0.0001 |
| L-Glutamine | Amino acid | 12665 | 1131 |  | 5353 | 611 | >0.0001 |
| D-Fructose 6-phosphate | Glycolysis | 3155 | 259 |  | 860 | 335 | >0.0001 |
| Glycine | Amino acid | 526 | 43 |  | 837 | 46 | >0.0001 |
| Mercaptopyruvate | Cystein metabolism | 3831 | 174 |  | 6061 | 423 | >0.0001 |
| N-Acetyl-L-glutamate | Glutamate metabolism | 762 | 72 |  | 1466 | 128 | >0.0001 |
| L-Proline | Amino acid | 16115 | 1062 |  | 25792 | 2366 | >0.0001 |
| Deoxyadenosine | Purine metabolism | 80 | 9 |  | 127 | 10 | >0.0001 |
| Erucic acid (22:1) | Fatty Acid | 75 | 9 |  | 123 | 10 | 0.0001 |
| Pyrophosphate | Phosphonate and phosphinate metabolism | 8986 | 853 |  | 17199 | 2269 | 0.0001 |
| L-Tyrosine | Amino acid | 3327 | 205 |  | 4727 | 372 | 0.0001 |
| L-Valine | Amino acid | 9791 | 437 |  | 13203 | 960 | 0.0001 |
| L-Ornithine | Urea Cycle | 259 | 28 |  | 445 | 51 | 0.0001 |
| L-Tryptophan | Amino acid | 594 | 37 |  | 965 | 112 | 0.0001 |
| L-Serine | Amino acid | 2204 | 67 |  | 1734 | 136 | 0.0001 |
| L-Phenylalanine | Amino acid | 5395 | 556 |  | 8164 | 724 | 0.0001 |
| Hydroxypropionic acid | Pyrimidine metabolism | 23 | 4 |  | 41 | 4 | 0.0001 |
| Lactose | Sugar | 934 | 150 |  | 1731 | 234 | 0.0002 |
| Butyrylcarnitine | Acyl carnitine | 13451 | 2805 |  | 24784 | 2828 | 0.0002 |
| (R)-3-Hydroxybutanoate | Butanoate metabolism | 530 | 14 |  | 854 | 68 | 0.0003 |
| β-D-Fructose 1,6-bisphosphate | Glycolysis | 103740 | 12557 |  | 168289 | 20358 | 0.0003 |
| Thiamine | Cofactors and Vitamins | 4816 | 462 |  | 6808 | 586 | 0.0003 |
| L-Lysine | Amino acid | 3697 | 220 |  | 4617 | 269 | 0.0004 |
| L-Alanine | Amino acid | 9445 | 359 |  | 12085 | 932 | 0.0004 |
| L-Leucine | Amino acid | 28214 | 1629 |  | 36740 | 2809 | 0.0004 |
| Betaine | Glycine, Serin metabolism | 15448 | 910 |  | 20424 | 1747 | 0.0005 |
| 1-Methylhistidine | Histidine metabolism | 835 | 39 |  | 1037 | 72 | 0.0006 |
| N6-(L-1,3-Dicarboxypropyl)-L-lysine | Lysine metabolism | 200 | 14 |  | 413 | 55 | 0.0006 |
| N8-Acetylspermidine | Polyamine | 70 | 8 |  | 44 | 8 | 0.0008 |
| 5-Hydroxyisophthalic Acid | Unclassified | 139 | 20 |  | 213 | 26 | 0.0009 |
| Taurocholate | Bile acid | 201 | 29 |  | 274 | 18 | 0.0014 |
| O-Acetylcarnitine | Acyl carnitine | 3371 | 289 |  | 8276 | 1516 | 0.0016 |
| Indoxyl | Tryptophan metabolism | 81 | 19 |  | 134 | 17 | 0.0017 |
| L-2-Aminoadipate | Lysine metabolism | 1172 | 53 |  | 908 | 117 | 0.0018 |
| 4-Pyridoxate | Cofactors and Vitamins | 194 | 15 |  | 247 | 24 | 0.0029 |
| Maleamate | Nicotinate and Ncotinamide metabolism | 8511 | 418 |  | 10579 | 1017 | 0.0030 |
| Choline phosphate | Phosphatidylcholine | 868 | 254 |  | 3496 | 985 | 0.0030 |
| Cortodoxone | Steroids | 19 | 13 |  | 45 | 6 | 0.0038 |
| N-N-dimethylarginine | Arginin, Proline metabolism | 2444 | 169 |  | 2006 | 179 | 0.0041 |
| L-Arginine | Amino acid | 3727 | 120 |  | 4605 | 376 | 0.0047 |
| 1-Kestose | Unclassified | 0 | 0 |  | 139 | 56 | 0.0051 |
| L-Cystathionine | Cystein metabolism | 5700 | 355 |  | 4636 | 521 | 0.0054 |
| Tauroursodeoxycholic acid | Bile acid | 28 | 5 |  | 40 | 5 | 0.0056 |
| L-Threonine | Amino acid | 12582 | 851 |  | 14296 | 566 | 0.0056 |
| beta-Alanine | β-Alanine metabolism | 247 | 19 |  | 319 | 39 | 0.0066 |
| 3-Hydroxy-L-proline | Arginin, Proline metabolism | 9883 | 474 |  | 12052 | 1248 | 0.0067 |
| L-Histidine | Amino acid | 4530 | 163 |  | 5226 | 399 | 0.0070 |
| Taurodeoxycholic acid | Bile acid | 94 | 9 |  | 114 | 3 | 0.0074 |
| L-Threonine phosphate | Glycine, Serin metabolism | 1008 | 112 |  | 1380 | 205 | 0.0074 |
| Docosahexaenoic acid (22:6;4,7,10,13,16,19) | Fatty Acid | 20063 | 2496 |  | 25292 | 2302 | 0.0088 |
| 3-Isopropylmalate | Amino acid metabolism | 95 | 18 |  | 135 | 18 | 0.0091 |
| Docosapentaenoate (n3 DPA; 22:5n3) | Fatty Acid | 12388 | 1895 |  | 17195 | 2606 | 0.0103 |
| 3-Methyl-2-oxovaleric acid | Valine, leucine and isoleucine metabolism | 16 | 4 |  | 26 | 5 | 0.0105 |
| Pyridoxal | Cofactors and Vitamins | 1368 | 221 |  | 1769 | 164 | 0.0115 |
| Methylimidazoleacetic acid | Histidine metabolism | 55 | 9 |  | 37 | 8 | 0.0133 |
| Lumichrome | Unclassified | 221 | 7 |  | 311 | 49 | 0.0137 |
| linoleate(18:2n6) | Fatty Acid | 1558 | 179 |  | 1957 | 227 | 0.0149 |
| NAD+ | Nicotinate and Ncotinamide metabolism | 274 | 78 |  | 471 | 119 | 0.0150 |
| Taurochenodeoxycholate | Bile acid | 451 | 83 |  | 576 | 38 | 0.0156 |
| Palmitoleic acid | Fatty Acid | 1176476 | 156805 |  | 1594087 | 262369 | 0.0157 |
| D-Erythronic-γ-lactone | Sugar metabolism | 982 | 60 |  | 1200 | 148 | 0.0158 |
| Myristoleate (14:1n5) | Fatty Acid | 128979 | 20426 |  | 194906 | 45622 | 0.0184 |
| Pentadecanoate (15:0) | Fatty Acid | 28049 | 4689 |  | 40764 | 8523 | 0.0192 |
| Stearidonic acid (18:4) | Fatty Acid | 275 | 44 |  | 379 | 68 | 0.0203 |
| L-Homoserine | Cystein metabolism | 82 | 8 |  | 95 | 6 | 0.0215 |
| Glycocholate | Bile acid | 72 | 15 |  | 96 | 11 | 0.0224 |
| Ricinoleic acid (18:1-OH) | Fatty Acid | 19548 | 1700 |  | 16607 | 1611 | 0.0229 |
| L-Erythrulose | Sugar | 540 | 44 |  | 658 | 84 | 0.0234 |
| Linolenic acid(18:3) | Fatty Acid | 16765 | 3222 |  | 24306 | 5164 | 0.0243 |
| L-Kynurenine | Tryptophan metabolism | 295 | 60 |  | 391 | 49 | 0.0245 |
| Arachidonate | Fatty Acid | 27294 | 6001 |  | 36817 | 5139 | 0.0273 |
| Glycochenodeoxycholate | Bile acid | 253 | 51 |  | 325 | 32 | 0.0273 |
| UDP (Uridine 5'-diphosphate) | Pyrimidine metabolism | 221 | 89 |  | 373 | 90 | 0.0279 |
| S-Adenosyl-L-homocysteine | Cystein metabolism | 70 | 5 |  | 79 | 6 | 0.0302 |
| (R)-Pantothenate | Cofactors and Vitamins | 163 | 29 |  | 228 | 47 | 0.0316 |
| Hypotaurine | Taurine and hypotaurine metabolism | 611 | 42 |  | 698 | 64 | 0.0346 |
| D-Octopine | Arginin, Proline metabolism | 20 | 4 |  | 29 | 7 | 0.0354 |
| 6-Hydroxymelatonin | Tryptophan metabolism | 188 | 53 |  | 261 | 37 | 0.0355 |
| 2-Isopropylmalate | Valine, leucine and isoleucine metabolism | 104 | 20 |  | 131 | 3 | 0.0366 |
| Uracil | Pyrimidine metabolism | 1193 | 196 |  | 1545 | 245 | 0.0367 |
| Pyridoxal 5-phosphate | Cofactors and Vitamins | 169 | 20 |  | 222 | 43 | 0.0378 |
| beta-D-Glucose | Sugar | 2115 | 472 |  | 2967 | 660 | 0.0469 |
| N6-Methyllysine | Lysine metabolism | 56 | 3 |  | 66 | 8 | 0.0478 |
| L-Gulono-1,4-lactone | Glucuronic acid metabolism | 647 | 58 |  | 722 | 44 | 0.0515 |
| 5-Aminovalerate | Amino acid metabolism | 457 | 110 |  | 302 | 105 | 0.0525 |
| Creatine | Urea Cycle | 38706 | 3931 |  | 33536 | 3291 | 0.0541 |
| L-Asparagine | Amino acid | 1882 | 107 |  | 2124 | 218 | 0.0571 |
| NADP+ | Nicotinate and Ncotinamide metabolism | 75 | 34 |  | 121 | 32 | 0.0580 |
| Heptadecanoic acid (17:0) | Fatty Acid | 29765 | 4802 |  | 40826 | 10110 | 0.0581 |
| Estriol | Steroids | 16 | 11 |  | 32 | 12 | 0.0583 |
| Glycerol-3-phosphate | Glycolysis | 1036 | 121 |  | 766 | 252 | 0.0626 |
| Phenylphosphate | Benzoate degradation | 177 | 45 |  | 224 | 19 | 0.0644 |
| Myristic acid (14:0) | Fatty Acid | 2482 | 603 |  | 3332 | 706 | 0.0748 |
| UDP-N-acetylglucosamine | Sugar metabolism | 695 | 212 |  | 449 | 180 | 0.0825 |
| 20α-Hydroxyprogesterone | Steroids | 93 | 15 |  | 144 | 50 | 0.0848 |
| 4-Methylaminobutyrate | Nicotinate and Ncotinamide metabolism | 1316 | 361 |  | 1765 | 364 | 0.0856 |
| Eicosenoic acid (20:1) | Fatty Acid | 184962 | 35124 |  | 240115 | 52698 | 0.0874 |
| 6-Aminohexanoate | Fatty acid metabolism | 5775 | 1403 |  | 7643 | 1668 | 0.0915 |
| Cytosine | Pyrimidine metabolism | 44 | 10 |  | 57 | 12 | 0.0951 |
| Deoxyguanosine | Purine metabolism | 48 | 10 |  | 26 | 25 | 0.0957 |
| Tridecanoic acid | Fatty Acid | 87 | 31 |  | 159 | 80 | 0.0965 |
| (S)-2-Acetoin | Unclassified | 81 | 35 |  | 42 | 34 | 0.1097 |
| Urocanic acid | Histidine metabolism | 92 | 19 |  | 68 | 23 | 0.1121 |
| 2-Oxobutanoate | Glycine, Serin metabolism | 49 | 6 |  | 59 | 12 | 0.1144 |
| trans-Zeatin-riboside | Unclassified | 59 | 13 |  | 78 | 20 | 0.1161 |
| Dihomo-linolenate (20:3n6) | Fatty Acid | 16083 | 2969 |  | 19759 | 3601 | 0.1162 |
| Arachidic acid (20:0) | Fatty Acid | 161 | 54 |  | 243 | 90 | 0.1166 |
| N,N-Dimethylaniline | Unclassified | 20 | 5 |  | 28 | 8 | 0.1209 |
| D-Erythrose 4-phosphate | Pentose phosphate pathway | 258 | 41 |  | 176 | 97 | 0.1223 |
| Phosphocreatine | Arginin, Proline metabolism | 146 | 44 |  | 272 | 143 | 0.1227 |
| 5'-Methylthioadenosine | Cystein metabolism | 21857 | 3421 |  | 32673 | 12374 | 0.1232 |
| 5-Methylcytidine | Nucleotide metabolism | 84 | 19 |  | 107 | 24 | 0.1334 |
| Docosatrienoic (22:3) | Fatty Acid | 74 | 10 |  | 90 | 19 | 0.1358 |
| L-Aspartate | Amino acid | 9731 | 614 |  | 10640 | 1163 | 0.1605 |
| Spermine | Polyamine | 498 | 80 |  | 646 | 201 | 0.1621 |
| Prostaglandin B2 | Prostaglandins | 38 | 10 |  | 49 | 12 | 0.1623 |
| Estriol-3-sulfate | Unclassified | 21 | 9 |  | 33 | 14 | 0.1625 |
| Xanthine | Purine metabolism | 4513 | 444 |  | 5386 | 1194 | 0.1638 |
| Adenine | Purine metabolism | 1200 | 75 |  | 1315 | 150 | 0.1660 |
| Pseudouridine | Pyrimidine metabolism | 1701 | 252 |  | 1955 | 276 | 0.1667 |
| L-Gulonic acid | Glucuronic acid metabolism | 77 | 12 |  | 87 | 9 | 0.1684 |
| Cytidine | Pyrimidine metabolism | 1728 | 83 |  | 1857 | 171 | 0.1684 |
| 5,6-Dihydrothymine | Pyrimidine metabolism | 235 | 43 |  | 278 | 50 | 0.1780 |
| Glutathione | Glutathione metabolism | 5623 | 2465 |  | 7539 | 1659 | 0.1873 |
| UMP | Pyrimidine metabolism | 653 | 126 |  | 481 | 237 | 0.1896 |
| L-Methionine | Amino acid | 477 | 80 |  | 573 | 129 | 0.1925 |
| Adenosine | Purine metabolism | 265 | 103 |  | 192 | 27 | 0.1941 |
| N-Acetylneuraminate | Urea Cycle | 140 | 21 |  | 101 | 58 | 0.1945 |
| Threonate | Ascorbate and Aldarate metabolism | 28764 | 1935 |  | 31499 | 3926 | 0.1997 |
| N5-Ethyl-L-glutamine (L-Theanine) | Glutamate metabolism | 89 | 14 |  | 99 | 10 | 0.2087 |
| N-Acetyl-DL-valine | Valine, leucine and isoleucine metabolism | 38 | 11 |  | 47 | 8 | 0.2112 |
| 2-Hydroxy-4-methylpentanoate | Organic acid | 73 | 18 |  | 91 | 25 | 0.2209 |
| Guanosine | Purine metabolism | 1966 | 791 |  | 2604 | 728 | 0.2210 |
| Cystine | Nucleotide metabolism | 13 | 17 |  | 2 | 4 | 0.2234 |
| O-Myristoyl-L-carnitine (14:0) | Acyl carnitine | 3806 | 1002 |  | 7566 | 5846 | 0.2256 |
| Mandelate | Organic acid | 43 | 22 |  | 61 | 20 | 0.2269 |
| 3-Hydroxy-3-methylglutarate | Organic acid | 73 | 9 |  | 86 | 21 | 0.2304 |
| 5-Dodecenoate (12:1n7) | Fatty Acid | 237 | 33 |  | 263 | 32 | 0.2359 |
| Dodecanoic acid (12:0) | Fatty Acid | 642 | 119 |  | 747 | 142 | 0.2407 |
| Isatin | Unclassified | 36 | 9 |  | 44 | 12 | 0.2436 |
| 6-Phospho-D-gluconate | Pentose phosphate pathway | 340 | 42 |  | 407 | 114 | 0.2515 |
| 18:2carnitine | Acyl carnitine | 220 | 90 |  | 375 | 270 | 0.2556 |
| 3-(4-Hydroxyphenyl) lactate | Phenylalanine Tyrosine metabolism | 27 | 6 |  | 35 | 12 | 0.2579 |
| L-Lactic acid | Glycolysis | 3126 | 1496 |  | 4215 | 1353 | 0.2617 |
| Oxidized glutathione | Glutathione metabolism | 1711 | 447 |  | 1962 | 150 | 0.2665 |
| 6-γ-γ-dimethylallyl-amino-purine | Plant metabolites | 61 | 54 |  | 30 | 14 | 0.2753 |
| Nicotinamide | Nicotinate and Ncotinamide metabolism | 14875 | 1215 |  | 15583 | 617 | 0.2791 |
| Deoxycytidine | Pyrimidine metabolism | 54 | 12 |  | 65 | 19 | 0.2917 |
| Phenyllactate | Phenylalanine Tyrosine metabolism | 113 | 19 |  | 128 | 24 | 0.3114 |
| (R)-Malate | Butanoate metabolism | 1242 | 492 |  | 1642 | 675 | 0.3154 |
| Arachidonoyl coenzyme A | Other Group | 470 | 233 |  | 328 | 202 | 0.3349 |
| Glycodeoxycholate | Bile acid | 51 | 12 |  | 58 | 6 | 0.3393 |
| UDP-glucose | Pyrimidine metabolism | 451 | 117 |  | 531 | 132 | 0.3400 |
| GDP | Purine metabolism | 36 | 18 |  | 45 | 12 | 0.3623 |
| beta-Amino iso butyric acid | Pyrimidine metabolism | 712 | 179 |  | 856 | 305 | 0.3898 |
| Nonanate (9:0) | Fatty Acid | 57 | 12 |  | 48 | 17 | 0.3960 |
| Thymine | Pyrimidine metabolism | 668 | 160 |  | 776 | 218 | 0.3964 |
| L-Glutamate | Amino acid | 61867 | 2963 |  | 65114 | 7567 | 0.3976 |
| 1-(5'-Phosphoribosyl)-5-amino-4-imidazolecarboxamide | Purine metabolism | 105 | 57 |  | 130 | 26 | 0.3990 |
| γ-L-glutamyl-L-tyrosine | Phenylalanine Tyrosine metabolism | 1015 | 166 |  | 1088 | 78 | 0.4001 |
| Dodecanedioate | Fatty acid metabolism | 21 | 4 |  | 25 | 11 | 0.4010 |
| L-Cysteine | Amino acid | 150 | 89 |  | 187 | 30 | 0.4066 |
| D-Glucosamine 6-phosphate | Sugar metabolism | 560 | 67 |  | 511 | 107 | 0.4102 |
| Indole acetate | Tryptophan metabolism | 49 | 17 |  | 56 | 7 | 0.4181 |
| γ-Glutamylphenylalanine | Phenylalanine Tyrosine metabolism | 40 | 4 |  | 37 | 8 | 0.4341 |
| Phosphoenolpyruvate | Glycolysis | 151 | 50 |  | 227 | 191 | 0.4351 |
| trans-4-Hydroxy-3-methoxycinnamate | Phenylpropanoid | 18 | 3 |  | 20 | 4 | 0.4397 |
| Prostaglandin E2 | Prostaglandins | 52 | 11 |  | 58 | 12 | 0.4575 |
| N2,N2-Dimethylguanosine | Nucleotide metabolism | 108 | 62 |  | 137 | 58 | 0.4627 |
| trans-Cinnamate | Phenylalanine Tyrosine metabolism | 374 | 195 |  | 466 | 183 | 0.4641 |
| GMP | Purine metabolism | 187 | 147 |  | 122 | 120 | 0.4672 |
| 1,4-Pregnadien-11 beta,17,21-triol-3-20-dione | Unclassified | 2 | 5 |  | 6 | 8 | 0.4714 |
| AMP | Purine metabolism | 651 | 113 |  | 712 | 143 | 0.4778 |
| Pyruvate | Glycolysis | 29 | 6 |  | 34 | 17 | 0.4994 |
| Ribitol | Sugar | 158 | 51 |  | 192 | 94 | 0.5006 |
| Succinic acid | Citrate cycle (TCA cycle) | 290 | 45 |  | 329 | 115 | 0.5044 |
| Anthranilate | Tryptophan metabolism | 1123 | 154 |  | 1215 | 256 | 0.5111 |
| 5-Oxoproline | Glutathione metabolism | 1692 | 165 |  | 1574 | 365 | 0.5290 |
| Pyridoxamine-phosphate | Cofactors and Vitamins | 134 | 23 |  | 144 | 26 | 0.5569 |
| Uridine | Pyrimidine metabolism | 54434 | 4162 |  | 56984 | 8533 | 0.5648 |
| S-Adenosyl-L-Methionine | Urea Cycle | 400 | 27 |  | 422 | 81 | 0.5724 |
| Creatinine | Arginin, Proline metabolism | 3697 | 308 |  | 3820 | 357 | 0.5759 |
| Choline | Glycine, Serin metabolism | 30301 | 5280 |  | 28265 | 5803 | 0.5777 |
| D-Ribulose 5-phosphate | Pentose phosphate pathway | 3505 | 618 |  | 3863 | 1251 | 0.5818 |
| L-Hydroxyproline | Arginin, Proline metabolism | 1127 | 303 |  | 1013 | 329 | 0.5861 |
| 1-Palmitoleoylglycerophosphocholine  (16:1) | Lyso PC | 99 | 85 |  | 128 | 85 | 0.6070 |
| Glycerone phosphate | Glycolysis | 451 | 34 |  | 424 | 115 | 0.6314 |
| N-Formyl-L-methionine | Cystein metabolism | 31 | 6 |  | 34 | 12 | 0.6400 |
| Isofraxidin | Flavonoids | 722 | 201 |  | 668 | 144 | 0.6409 |
| Coenzyme A | Cofactors and Vitamins | 38 | 22 |  | 32 | 11 | 0.6521 |
| (R)-S-Lactoylglutathione | Pyruvate metabolism | 17 | 8 |  | 15 | 9 | 0.6696 |
| D-Sedoheptulose 7-phosphate | Pentose phosphate pathway | 1572 | 455 |  | 1380 | 857 | 0.6707 |
| 1-Methyladenosine | Nucleotide metabolism | 124 | 14 |  | 129 | 20 | 0.6708 |
| Glycolate | Glyoxylate and dicarboxylate metabolism | 23 | 8 |  | 26 | 11 | 0.6709 |
| N6-Acetyl-L-lysine | Lysine metabolism | 46 | 8 |  | 52 | 31 | 0.6752 |
| Hydroxyprogesterone caproate | Sterol metabolism | 41 | 6 |  | 38 | 14 | 0.6768 |
| Carnosine | β-Alanine metabolism | 26 | 3 |  | 25 | 2 | 0.6951 |
| N-alpha-Acetyl-L-arginine | Arginin, Proline metabolism | 211 | 33 |  | 219 | 33 | 0.6969 |
| GDP-L-fucose | Sugar metabolism | 30 | 11 |  | 27 | 13 | 0.7077 |
| N-Acetylthreonine | Glycine, Serin metabolism | 366 | 52 |  | 348 | 92 | 0.7153 |
| Riboflavin | Cofactors and Vitamins | 38 | 15 |  | 35 | 8 | 0.7259 |
| Eicosadienoic acid (20:2) | Fatty Acid | 59119 | 9741 |  | 61654 | 12742 | 0.7329 |
| N-Acetyl-D-glucosamine | Sugar | 436 | 52 |  | 453 | 102 | 0.7432 |
| 3',5'-Cyclic AMP | Purine metabolism | 56 | 56 |  | 67 | 63 | 0.7893 |
| D-Glycerate | Glycine, Serin metabolism | 61 | 8 |  | 63 | 21 | 0.8299 |
| 5'-CMP | Pyrimidine metabolism | 1034 | 582 |  | 1118 | 654 | 0.8344 |
| N-Acetyl-L-phenylalanine | Phenylalanine Tyrosine metabolism | 146 | 32 |  | 151 | 40 | 0.8390 |
| 1-6-Anhydro-beta-d-Glucose | Sugar metabolism | 230 | 178 |  | 212 | 92 | 0.8473 |
| N2-Acetyl-L-lysine | Lysine metabolism | 45 | 5 |  | 44 | 13 | 0.8806 |
| L-Citrulline | Urea Cycle | 40 | 6 |  | 41 | 7 | 0.8909 |
| L-Carnitine | Lysine metabolism | 23292 | 2427 |  | 23492 | 2794 | 0.9068 |
| Hypoxanthine | Purine metabolism | 16527 | 1441 |  | 16604 | 1295 | 0.9314 |
| Citrate | Citrate cycle (TCA cycle) | 155 | 36 |  | 157 | 36 | 0.9322 |
| L-Cysteine-glutathione disulfide | Other Group | 494 | 228 |  | 486 | 36 | 0.9462 |
| N-Acetyl-L-methionine | Cystein metabolism | 46 | 16 |  | 47 | 14 | 0.9521 |
| Cortisone | Steroids | 38 | 3 |  | 38 | 15 | 0.9556 |
| Abscisate | Plant metabolites | 36 | 6 |  | 35 | 7 | 0.9636 |
| Oxypurinol | Lipid metabolism | 1092 | 334 |  | 1102 | 565 | 0.9726 |
| Capric acid (10:0) | Fatty Acid | 2747 | 191 |  | 2738 | 536 | 0.9726 |
| Prostaglandin F2α | Prostaglandins | 735 | 151 |  | 737 | 96 | 0.9807 |
| Ophthalmic Acid | Glutathione metabolism | 26 | 5 |  | 26 | 4 | 1.0000 |
