# Supplementary material for: Effects of canagliflozin on growth and metabolic reprograming in hepatocellular carcinoma cells: Multi-omics analysis of metabolomics and absolute quantification proteomics (iMPAQT)
Source: PLoS One. 2020 Apr 28;15(4):e0232283. doi: 10.1371/journal.pone.0232283 (PMC7188283; doi:10.1371/journal.pone.0232283)
Supplement: S2 Table — (DOCX) [file pone.0232283.s011.docx]

Supplementary Table 2. Effects of CANA on expression level of 342 metabolic enzymes by iMPAQT assay in Hep3B cells

| Enzyme | Pathway | Control | |  | SGLT2i | | P |
| --- | --- | --- | --- | --- | --- | --- | --- |
|  |  | Mean | SD |  | Mean | SD |  |
| UQCRQ | Oxidative phosphorylation | 28.99 | 1.38 |  | 35.53 | 1.39 | 0.0002 |
| NDUFS2 | Oxidative phosphorylation | 34.84 | 0.77 |  | 39.48 | 1.26 | 0.0002 |
| NDUFB7 | Oxidative phosphorylation | 25.08 | 2.96 |  | 34.58 | 3.36 | 0.0028 |
| NDUFA9 | Oxidative phosphorylation | 41.7 | 2.44 |  | 49.16 | 2.62 | 0.0031 |
| COX7A2 | Oxidative phosphorylation | 83.33 | 10.93 |  | 111.67 | 9.13 | 0.0041 |
| NDUFV1 | Oxidative phosphorylation | 19.95 | 0.92 |  | 23.63 | 1.75 | 0.0059 |
| NDUFV2 | Oxidative phosphorylation | 34.97 | 1.03 |  | 39.36 | 2.17 | 0.0065 |
| UQCRC2 | Oxidative phosphorylation | 51.12 | 4.56 |  | 64.31 | 5.91 | 0.0077 |
| NDUFB4 | Oxidative phosphorylation | 25.42 | 1.21 |  | 28.65 | 1.58 | 0.0118 |
| ATP5A1 | Oxidative phosphorylation | 297.28 | 20.63 |  | 251.83 | 22.83 | 0.0183 |
| COX4I1 | Oxidative phosphorylation | 136.81 | 5.26 |  | 150.79 | 8.03 | 0.0195 |
| PRIM2 | Purine, Pyrimidine metabolism | 5.99 | 0.14 |  | 5.09 | 0.26 | 0.0003 |
| RRM1 | Purine, Pyrimidine metabolism | 23.03 | 0.71 |  | 17.31 | 2.2 | 0.0011 |
| ITPA | Purine, Pyrimidine metabolism | 27.46 | 2.17 |  | 22.17 | 1.31 | 0.0031 |
| ADSS | Purine, Pyrimidine metabolism | 26.76 | 0.86 |  | 22.79 | 2.18 | 0.0095 |
| NME1 | Purine, Pyrimidine metabolism | 110.3 | 11.37 |  | 89.14 | 8.39 | 0.0172 |
| NT5C2 | Purine, Pyrimidine metabolism | 21.82 | 1.06 |  | 19.31 | 1.55 | 0.0281 |
| PNP | Purine, Pyrimidine metabolism | 14.7 | 1.89 |  | 12.23 | 0.72 | 0.0408 |
| GPT2 | Arginine and proline metabolism | 10.41 | 1.32 |  | 19.14 | 2.32 | 0.0002 |
| GLUD1 | Arginine and proline metabolism | 167.21 | 7.59 |  | 129.09 | 9.27 | 0.0002 |
| PYCR1 | Arginine and proline metabolism | 8.82 | 0.87 |  | 11.86 | 1.35 | 0.0053 |
| PYCR2 | Arginine and proline metabolism | 30.88 | 3.28 |  | 36.72 | 1.49 | 0.0119 |
| CKB | Arginine and proline metabolism | 65.96 | 8.75 |  | 52.52 | 5.72 | 0.0331 |
| OAT | Arginine and proline metabolism | 16.32 | 1.1 |  | 18.79 | 1.63 | 0.0359 |
| SCD | Fatty acid metabolism | 90.82 | 5.18 |  | 68.71 | 5.07 | 0.0003 |
| ACOT7 | Fatty acid metabolism | 19.96 | 1.07 |  | 15.44 | 1.52 | 0.0012 |
| ACAT2 | Fatty acid metabolism | 55.37 | 3.71 |  | 40.13 | 6.47 | 0.0035 |
| ACAA1 | Fatty acid metabolism | 35.46 | 2.4 |  | 30.44 | 2.53 | 0.0206 |
| PHGDH | Glycine, serine and threonine metabolism | 41.67 | 2.53 |  | 67.88 | 6 | <0.0001 |
| SHMT2 | Glycine, serine and threonine metabolism | 155.76 | 4.86 |  | 194.1 | 13.99 | 0.0008 |
| PSAT1 | Glycine, serine and threonine metabolism | 109.65 | 12.76 |  | 170.17 | 24.36 | 0.0023 |
| CBS | Glycine, serine and threonine metabolism | 59.75 | 7.89 |  | 72.84 | 6.67 | 0.035 |
| PKM2 | Glycolysis / Gluconeogenesis | 224.14 | 13.69 |  | 190.53 | 17.28 | 0.0159 |
| LDHA | Glycolysis / Gluconeogenesis | 197.54 | 11.83 |  | 163.32 | 19.95 | 0.0184 |
| ENO1 | Glycolysis / Gluconeogenesis | 614.47 | 41.47 |  | 509.49 | 62.87 | 0.0236 |
| GAPDH | Glycolysis / Gluconeogenesis | 1642.62 | 161.94 |  | 1369.61 | 130 | 0.0302 |
| PRPS2 | Pentose phosphate pathway | 18.68 | 1.91 |  | 15.36 | 1.2 | 0.0188 |
| ALDOA | Pentose phosphate pathway | 589.18 | 87.37 |  | 460.4 | 41.25 | 0.0285 |
| PGM1 | Pentose phosphate pathway | 8.18 | 0.59 |  | 6.95 | 0.85 | 0.0437 |
| GPI | Pentose phosphate pathway | 141.05 | 15.93 |  | 118.88 | 10.23 | 0.0473 |
| BCAT1 | Valine, leucine and isoleucine metabolism | 8.62 | 0.58 |  | 12.04 | 1.55 | 0.0033 |
| MUT | Valine, leucine and isoleucine metabolism | 8.83 | 0.6 |  | 10.54 | 0.69 | 0.0055 |
| IVD | Valine, leucine and isoleucine metabolism | 42.2 | 1.69 |  | 47.59 | 4.1 | 0.0411 |
| LPGAT1 | Glycerophospholipid metabolism | 47.08 | 4.29 |  | 40.23 | 3.47 | 0.0381 |
| LPCAT1 | Glycerophospholipid metabolism | 10.58 | 0.96 |  | 9.26 | 0.54 | 0.0433 |
| HMOX1 | Porphyrin and chlorophyll metabolism | 20.73 | 1.31 |  | 15.94 | 1.82 | 0.0027 |
| HMOX2 | Porphyrin and chlorophyll metabolism | 19.5 | 0.42 |  | 17.93 | 1.16 | 0.0344 |
| TXNRD1 | Pyrimidine metabolism | 21.63 | 1.25 |  | 33.04 | 2.47 | <0.0001 |
| CTPS | Pyrimidine metabolism | 13.09 | 1.07 |  | 10.72 | 1.46 | 0.031 |
| ME1 | Pyruvate metabolism | 22.97 | 2.89 |  | 28 | 1.7 | 0.0171 |
| GLO1 | Pyruvate metabolism | 37.9 | 7.06 |  | 26.46 | 3.08 | 0.0179 |
| LIPA | Steroid biosynthesis | 7.57 | 0.76 |  | 5.42 | 0.74 | 0.0037 |
| FDFT1 | Steroid biosynthesis | 9.71 | 1.13 |  | 7.34 | 1.05 | 0.0153 |
| ASNS | Alanine, aspartate and glutamate metabolism | 40.79 | 3.82 |  | 65.41 | 5.02 | 0.0001 |
| CMAS | Amino sugar and nucleotide sugar metabolism | 37.53 | 3.21 |  | 30.06 | 2.23 | 0.0051 |
| WARS | Aminoacyl-tRNA biosynthesis | 23.95 | 2.8 |  | 31.26 | 4.54 | 0.0253 |
| MAT2A | Cysteine and methionine metabolism | 51.83 | 4.87 |  | 39.93 | 4.37 | 0.0066 |
| PAFAH1B3 | Ether lipid metabolism | 23.57 | 3.71 |  | 19.03 | 1.23 | 0.0485 |
| GGH | Folate biosynthesis | 19.69 | 1.47 |  | 15.73 | 2.12 | 0.0155 |
| GMPPA | Fructose and mannose metabolism | 13.11 | 1.29 |  | 10.85 | 0.69 | 0.0149 |
| IMPA1 | Inositol phosphate metabolism | 39.71 | 2.13 |  | 35.59 | 2.52 | 0.0373 |
| AASDHPPT | Pantothenate and CoA biosynthesis | 19.1 | 1.9 |  | 14.65 | 1.19 | 0.0041 |
| DCXR | Pentose and glucuronate interconversions | 33.75 | 2.17 |  | 40.95 | 1.17 | 0.0004 |
| AGL | Starch and sucrose metabolism | 3.18 | 0.3 |  | 2.11 | 0.43 | 0.0032 |
| TST | Sulfur metabolism | 30.86 | 3.36 |  | 24.81 | 2.7 | 0.0231 |
| HMGCS1 | Synthesis and degradation of ketone bodies | 40.51 | 4.36 |  | 22.77 | 3.58 | 0.0002 |
| CAT | Tryptophan metabolism | 76.11 | 4.42 |  | 61.09 | 10.95 | 0.0345 |
| CS | Citrate cycle (TCA cycle) | 618.44 | 561.04 |  | 123.69 | 112.21 | 0.0511 |
| GMPPB | Fructose and mannose metabolism | 48.55 | 40.38 |  | 9.71 | 8.08 | 0.0521 |
| SMS | Arginine and proline metabolism | 216.82 | 146.89 |  | 43.36 | 29.38 | 0.0523 |
| AHCYL1 | Cysteine and methionine metabolism | 33.61 | 24.60 |  | 6.72 | 4.92 | 0.0525 |
| PLOD2 | Lysine degradation | 103.35 | 93.70 |  | 20.67 | 18.74 | 0.0547 |
| DCI | Fatty acid degradation | 927.41 | 1061.06 |  | 185.48 | 212.21 | 0.0659 |
| TXNRD2 | Pyrimidine metabolism | 88.43 | 100.23 |  | 17.69 | 20.05 | 0.0671 |
| UMPS | Pyrimidine metabolism | 142.07 | 112.18 |  | 28.41 | 22.44 | 0.0724 |
| GK | Glycerolipid metabolism | 53.48 | 64.84 |  | 10.70 | 12.97 | 0.0726 |
| IARS | Aminoacyl-tRNA biosynthesis | 177.56 | 205.30 |  | 35.51 | 41.06 | 0.0750 |
| IDH3B | Citrate cycle (TCA cycle) | 165.82 | 141.67 |  | 33.16 | 28.33 | 0.0751 |
| ACP1 | Adherens junction | 80.79 | 65.15 |  | 16.16 | 13.03 | 0.0782 |
| NDUFS7 | Oxidative phosphorylation | 126.07 | 145.62 |  | 25.21 | 29.12 | 0.0828 |
| ATP6AP1 | Oxidative phosphorylation | 93.49 | 80.31 |  | 18.70 | 16.06 | 0.0864 |
| TK1 | Pyrimidine metabolism | 78.72 | 63.19 |  | 15.74 | 12.64 | 0.0867 |
| VARS | Aminoacyl-tRNA biosynthesis | 503.35 | 445.97 |  | 100.67 | 89.19 | 0.0873 |
| ISYNA1 | Inositol phosphate metabolism | 77.33 | 67.79 |  | 15.47 | 13.56 | 0.0890 |
| FDPS | Terpenoid backbone biosynthesis | 422.85 | 354.40 |  | 84.57 | 70.88 | 0.0890 |
| AK2 | Purine metabolism | 431.15 | 461.84 |  | 86.23 | 92.37 | 0.0898 |
| SDHB | Citrate cycle (TCA cycle) | 320.42 | 345.61 |  | 64.08 | 69.12 | 0.0955 |
| PKM1 | Glycolysis / Gluconeogenesis | 93.77 | 104.42 |  | 18.75 | 20.88 | 0.0973 |
| GLT25D1 | Lysine degradation | 217.88 | 186.97 |  | 43.58 | 37.39 | 0.0975 |
| GPX7 | Glutathione metabolism | 37.51 | 32.91 |  | 7.50 | 6.58 | 0.1017 |
| ATP5H | Oxidative phosphorylation | 455.76 | 434.73 |  | 91.15 | 86.95 | 0.1034 |
| COASY | Pantothenate and CoA biosynthesis | 84.26 | 91.45 |  | 16.85 | 18.29 | 0.1044 |
| ADPGK | Glycolysis / Gluconeogenesis | 41.03 | 33.50 |  | 8.21 | 6.70 | 0.1071 |
| DHCR24 | Steroid biosynthesis | 164.94 | 144.50 |  | 32.99 | 28.90 | 0.1083 |
| UGP2 | Pentose and glucuronate interconversions | 189.11 | 169.96 |  | 37.82 | 33.99 | 0.1134 |
| PAICS | Purine metabolism | 976.94 | 857.01 |  | 195.39 | 171.40 | 0.1149 |
| PECI | Fatty acid degradation | 161.48 | 176.56 |  | 32.30 | 35.31 | 0.1190 |
| LSS | Steroid biosynthesis | 79.42 | 71.81 |  | 15.88 | 14.36 | 0.1206 |
| WHSC1 | Lysine degradation | 41.50 | 37.16 |  | 8.30 | 7.43 | 0.1234 |
| SORD | Pentose and glucuronate interconversions | 149.27 | 128.74 |  | 29.85 | 25.75 | 0.1279 |
| DERA | Pentose phosphate pathway | 53.84 | 45.73 |  | 10.77 | 9.15 | 0.1279 |
| ACO1 | Citrate cycle (TCA cycle) | 46.25 | 39.26 |  | 9.25 | 7.85 | 0.1297 |
| ATP5I | Oxidative phosphorylation | 722.70 | 653.45 |  | 144.54 | 130.69 | 0.1321 |
| HSD17B10 | Valine, leucine and isoleucine degradation | 407.83 | 370.86 |  | 81.57 | 74.17 | 0.1351 |
| HNMT | Histidine metabolism | 48.53 | 41.30 |  | 9.71 | 8.26 | 0.1399 |
| SOAT1 | Steroid biosynthesis | 242.76 | 226.00 |  | 48.55 | 45.20 | 0.1412 |
| NT5E | Purine metabolism | 30.28 | 34.14 |  | 6.06 | 6.83 | 0.1449 |
| PLOD1 | Lysine degradation | 181.73 | 167.06 |  | 36.35 | 33.41 | 0.1545 |
| ATIC | Purine metabolism | 642.17 | 569.80 |  | 128.43 | 113.96 | 0.1546 |
| POLR2H | Purine metabolism | 240.12 | 221.97 |  | 48.02 | 44.39 | 0.1650 |
| DCK | Purine metabolism | 165.91 | 146.00 |  | 33.18 | 29.20 | 0.1655 |
| HSP90AA1 | Antigen processing and presentation | 6001.53 | 5406.16 |  | 1200.31 | 1081.23 | 0.1721 |
| POLR2E | Purine metabolism | 111.59 | 102.40 |  | 22.32 | 20.48 | 0.1774 |
| ALDH18A1 | Arginine and proline metabolism | 78.52 | 70.60 |  | 15.70 | 14.12 | 0.1801 |
| GPD2 | Glycerophospholipid metabolism | 69.50 | 63.18 |  | 13.90 | 12.64 | 0.1811 |
| ZCCHC10 | Terpenoid backbone biosynthesis | 123.86 | 99.86 |  | 24.77 | 19.97 | 0.1813 |
| ATP6V1A | Oxidative phosphorylation | 296.39 | 270.71 |  | 59.28 | 54.14 | 0.1862 |
| NUDT5 | Purine metabolism | 196.13 | 180.45 |  | 39.23 | 36.09 | 0.1873 |
| POLR1E | Purine metabolism | 74.39 | 91.98 |  | 14.88 | 18.40 | 0.1902 |
| COMT | Steroid hormone biosynthesis | 258.26 | 277.33 |  | 51.65 | 55.47 | 0.1932 |
| NANS | Amino sugar and nucleotide sugar metabolism | 105.78 | 116.40 |  | 21.16 | 23.28 | 0.1947 |
| HADH | Fatty acid elongation | 131.19 | 142.51 |  | 26.24 | 28.50 | 0.1991 |
| FAH | Tyrosine metabolism | 97.17 | 108.24 |  | 19.43 | 21.65 | 0.2142 |
| GRHPR | Glycine, serine and threonine metabolism | 67.83 | 56.94 |  | 13.57 | 11.39 | 0.2219 |
| PSPH | Glycine, serine and threonine metabolism | 282.86 | 308.38 |  | 56.57 | 61.68 | 0.2303 |
| PGD | Pentose phosphate pathway | 467.76 | 430.19 |  | 93.55 | 86.04 | 0.2364 |
| POLR2G | Purine metabolism | 73.20 | 61.27 |  | 14.64 | 12.25 | 0.2374 |
| NIT2 | Alanine, aspartate and glutamate metabolism | 330.10 | 361.46 |  | 66.02 | 72.29 | 0.2482 |
| IDH2 | Citrate cycle (TCA cycle) | 680.85 | 651.24 |  | 136.17 | 130.25 | 0.2482 |
| TSTA3 | Fructose and mannose metabolism | 134.20 | 120.69 |  | 26.84 | 24.14 | 0.2489 |
| AK1 | Purine metabolism | 60.94 | 53.79 |  | 12.19 | 10.76 | 0.2547 |
| NDUFA4 | Oxidative phosphorylation | 277.07 | 309.18 |  | 55.41 | 61.84 | 0.2587 |
| PRDX6 | Metabolic pathways | 1086.18 | 1002.69 |  | 217.24 | 200.54 | 0.2598 |
| TUBB2A | Gap junction | 7363.48 | 6712.15 |  | 1472.70 | 1342.43 | 0.2657 |
| UQCRFS1 | Oxidative phosphorylation | 286.07 | 315.33 |  | 57.21 | 63.07 | 0.2662 |
| PDHA1 | Glycolysis / Gluconeogenesis | 295.72 | 338.35 |  | 59.14 | 67.67 | 0.2677 |
| CMPK1 | Pyrimidine metabolism | 746.78 | 674.36 |  | 149.36 | 134.87 | 0.2700 |
| LYPLA2 | Glycerophospholipid metabolism | 349.37 | 388.13 |  | 69.87 | 77.63 | 0.2741 |
| PFKL | Glycolysis / Gluconeogenesis | 84.41 | 91.12 |  | 16.88 | 18.22 | 0.2778 |
| ACTBL2 | Focal adhesion | 4586.49 | 4350.15 |  | 917.30 | 870.03 | 0.2780 |
| AGPS | Ether lipid metabolism | 281.02 | 324.25 |  | 56.20 | 64.85 | 0.2823 |
| GGPS1 | Terpenoid backbone biosynthesis | 31.89 | 26.73 |  | 6.38 | 5.35 | 0.2827 |
| ATP6V1B2 | Oxidative phosphorylation | 169.08 | 156.07 |  | 33.82 | 31.21 | 0.2903 |
| SUCLA2 | Citrate cycle (TCA cycle) | 84.62 | 78.22 |  | 16.92 | 15.64 | 0.2920 |
| PYCRL | Arginine and proline metabolism | 19.00 | 17.10 |  | 3.80 | 3.42 | 0.2957 |
| ACO2 | Citrate cycle (TCA cycle) | 184.76 | 201.46 |  | 36.95 | 40.29 | 0.3007 |
| ENOPH1 | Cysteine and methionine metabolism | 108.61 | 95.24 |  | 21.72 | 19.05 | 0.3041 |
| HADHB | Fatty acid elongation | 371.59 | 358.21 |  | 74.32 | 71.64 | 0.3042 |
| CYB5R3 | Amino sugar and nucleotide sugar metabolism | 816.09 | 770.59 |  | 163.22 | 154.12 | 0.3080 |
| HK2 | Glycolysis / Gluconeogenesis | 152.41 | 167.46 |  | 30.48 | 33.49 | 0.3170 |
| PMM2 | Fructose and mannose metabolism | 72.49 | 65.91 |  | 14.50 | 13.18 | 0.3197 |
| HSD17B12 | Fatty acid elongation | 654.35 | 608.86 |  | 130.87 | 121.77 | 0.3233 |
| GOT1 | Arginine biosynthesis | 254.49 | 270.25 |  | 50.90 | 54.05 | 0.3240 |
| ATP6V1D | Oxidative phosphorylation | 53.90 | 46.00 |  | 10.78 | 9.20 | 0.3273 |
| ATP5D | Oxidative phosphorylation | 834.34 | 785.40 |  | 166.87 | 157.08 | 0.3279 |
| LAP3 | Arginine and proline metabolism | 224.34 | 239.09 |  | 44.87 | 47.82 | 0.3332 |
| GNPDA1 | Amino sugar and nucleotide sugar metabolism | 130.32 | 123.06 |  | 26.06 | 24.61 | 0.3387 |
| HYI | Glyoxylate and dicarboxylate metabolism | 15.23 | 16.21 |  | 3.05 | 3.24 | 0.3414 |
| ALDH3A2 | Glycolysis / Gluconeogenesis | 158.38 | 148.79 |  | 31.68 | 29.76 | 0.3458 |
| UXS1 | Starch and sucrose metabolism | 88.66 | 96.86 |  | 17.73 | 19.37 | 0.3462 |
| ALDH9A1 | Glycolysis / Gluconeogenesis | 106.06 | 96.29 |  | 21.21 | 19.26 | 0.3515 |
| MPST | Sulfur metabolism | 204.57 | 190.72 |  | 40.91 | 38.14 | 0.3571 |
| NSDHL | Steroid biosynthesis | 181.50 | 169.63 |  | 36.30 | 33.93 | 0.3596 |
| PLOD3 | Lysine degradation | 80.37 | 87.56 |  | 16.07 | 17.51 | 0.3599 |
| ACADM | Fatty acid degradation | 209.68 | 227.38 |  | 41.94 | 45.48 | 0.3630 |
| TALDO1 | Pentose phosphate pathway | 385.73 | 402.91 |  | 77.15 | 80.58 | 0.3658 |
| NUDT9 | Purine metabolism | 44.57 | 40.02 |  | 8.91 | 8.00 | 0.3710 |
| PYGB | Starch and sucrose metabolism | 72.58 | 67.42 |  | 14.52 | 13.48 | 0.3713 |
| IARS2 | Aminoacyl-tRNA biosynthesis | 235.11 | 219.70 |  | 47.02 | 43.94 | 0.3733 |
| BLVRA | Porphyrin and chlorophyll metabolism | 51.38 | 46.75 |  | 10.28 | 9.35 | 0.3803 |
| PGK1 | Glycolysis / Gluconeogenesis | 769.77 | 722.36 |  | 153.95 | 144.47 | 0.3907 |
| TECR | Fatty acid elongation | 204.50 | 226.55 |  | 40.90 | 45.31 | 0.3972 |
| ATP6V1H | Oxidative phosphorylation | 47.28 | 42.96 |  | 9.46 | 8.59 | 0.3997 |
| ATP6V0D1 | Oxidative phosphorylation | 186.94 | 196.52 |  | 37.39 | 39.30 | 0.4048 |
| RDH11 | Retinol metabolism | 920.63 | 848.52 |  | 184.13 | 169.70 | 0.4056 |
| PCYT2 | Glycerophospholipid metabolism | 77.04 | 70.06 |  | 15.41 | 14.01 | 0.4151 |
| LYPLA1 | Glycerophospholipid metabolism | 124.76 | 118.08 |  | 24.95 | 23.62 | 0.4177 |
| MTHFD1 | One carbon pool by folate | 221.10 | 206.07 |  | 44.22 | 41.21 | 0.4183 |
| BPNT1 | Sulfur metabolism | 89.25 | 83.44 |  | 17.85 | 16.69 | 0.4233 |
| POLD3 | Purine metabolism | 35.94 | 33.96 |  | 7.19 | 6.79 | 0.4265 |
| RRM2B | Purine metabolism | 13.91 | 15.01 |  | 2.78 | 3.00 | 0.4298 |
| NDUFA5 | Oxidative phosphorylation | 198.12 | 208.19 |  | 39.62 | 41.64 | 0.4359 |
| ATP5B | Oxidative phosphorylation | 1314.65 | 1357.42 |  | 262.93 | 271.48 | 0.4366 |
| POLE3 | Purine metabolism | 115.19 | 106.48 |  | 23.04 | 21.30 | 0.4416 |
| ATP5E | Oxidative phosphorylation | 905.68 | 840.93 |  | 181.14 | 168.19 | 0.4529 |
| B4GALT1 | Galactose metabolism | 76.56 | 73.46 |  | 15.31 | 14.69 | 0.4611 |
| POLR2F | Purine metabolism | 116.49 | 118.88 |  | 23.30 | 23.78 | 0.4705 |
| ACAT1 | Fatty acid degradation | 383.92 | 365.53 |  | 76.78 | 73.11 | 0.4734 |
| ATP5L | Oxidative phosphorylation | 580.89 | 558.36 |  | 116.18 | 111.67 | 0.4863 |
| ATP5C1 | Oxidative phosphorylation | 616.87 | 589.82 |  | 123.37 | 117.96 | 0.4926 |
| FTH1 | Mineral absorption | 249.26 | 228.69 |  | 49.85 | 45.74 | 0.4944 |
| UAP1L1 | Amino sugar and nucleotide sugar metabolism | 44.04 | 40.85 |  | 8.81 | 8.17 | 0.4995 |
| AHCY | Cysteine and methionine metabolism | 359.45 | 345.52 |  | 71.89 | 69.10 | 0.5035 |
| QDPR | Folate biosynthesis | 191.10 | 181.71 |  | 38.22 | 36.34 | 0.5036 |
| AGK | Glycerolipid metabolism | 97.22 | 91.78 |  | 19.44 | 18.36 | 0.5046 |
| ATP6V1C1 | Oxidative phosphorylation | 80.50 | 77.37 |  | 16.10 | 15.47 | 0.5091 |
| NDUFA6 | Oxidative phosphorylation | 275.68 | 286.88 |  | 55.14 | 57.38 | 0.5203 |
| ECHS1 | Fatty acid elongation | 398.44 | 389.16 |  | 79.69 | 77.83 | 0.5348 |
| PPAT | Purine metabolism | 88.49 | 93.17 |  | 17.70 | 18.63 | 0.5524 |
| MINPP1 | Glycolysis / Gluconeogenesis | 128.94 | 138.66 |  | 25.79 | 27.73 | 0.5552 |
| PHPT1 | Unknown | 70.99 | 66.30 |  | 14.20 | 13.26 | 0.5572 |
| ATP5O | Oxidative phosphorylation | 619.71 | 596.96 |  | 123.94 | 119.39 | 0.5629 |
| PAPSS2 | Purine metabolism | 76.37 | 73.08 |  | 15.27 | 14.62 | 0.5661 |
| DHFR | One carbon pool by folate | 51.80 | 59.35 |  | 10.36 | 11.87 | 0.5804 |
| IDH3A | Citrate cycle (TCA cycle) | 148.43 | 143.22 |  | 29.69 | 28.64 | 0.5886 |
| PAFAH1B2 | Ether lipid metabolism | 152.21 | 138.42 |  | 30.44 | 27.68 | 0.5943 |
| MDH1 | Citrate cycle (TCA cycle) | 510.08 | 492.44 |  | 102.02 | 98.49 | 0.6081 |
| TUBA1A | Gap junction | 5440.43 | 5279.44 |  | 1088.09 | 1055.89 | 0.6130 |
| ALDH2 | Glycolysis / Gluconeogenesis | 258.28 | 268.50 |  | 51.66 | 53.70 | 0.6176 |
| POLR2L | Purine metabolism | 138.81 | 133.48 |  | 27.76 | 26.70 | 0.6190 |
| SGPL1 | Sphingolipid metabolism | 56.01 | 57.03 |  | 11.20 | 11.41 | 0.6203 |
| NMNAT1 | Nicotinate and nicotinamide metabolism | 15.90 | 15.39 |  | 3.18 | 3.08 | 0.6252 |
| PRIM1 | Purine metabolism | 27.80 | 29.01 |  | 5.56 | 5.80 | 0.6261 |
| APIP | Cysteine and methionine metabolism | 33.02 | 31.65 |  | 6.60 | 6.33 | 0.6276 |
| EPHX2 | Arachidonic acid metabolism | 24.61 | 25.43 |  | 4.92 | 5.09 | 0.6361 |
| PRPS1 | Pentose phosphate pathway | 120.66 | 115.41 |  | 24.13 | 23.08 | 0.6369 |
| GYS1 | Starch and sucrose metabolism | 41.49 | 39.67 |  | 8.30 | 7.93 | 0.6537 |
| ATP6V1F | Oxidative phosphorylation | 34.20 | 36.52 |  | 6.84 | 7.30 | 0.6544 |
| SRM | Arginine and proline metabolism | 221.15 | 227.10 |  | 44.23 | 45.42 | 0.6551 |
| DLD | Glycolysis / Gluconeogenesis | 298.06 | 287.52 |  | 59.61 | 57.50 | 0.6585 |
| GBA | Other glycan degradation | 114.05 | 117.29 |  | 22.81 | 23.46 | 0.6648 |
| SUCLG2 | Citrate cycle (TCA cycle) | 277.24 | 283.92 |  | 55.45 | 56.78 | 0.6729 |
| HPRT1 | Purine metabolism | 92.30 | 94.07 |  | 18.46 | 18.81 | 0.6883 |
| SMPD4 | Sphingolipid metabolism | 40.03 | 38.71 |  | 8.01 | 7.74 | 0.6926 |
| ACP2 | Lysosome | 59.02 | 60.52 |  | 11.80 | 12.10 | 0.6944 |
| PYGL | Starch and sucrose metabolism | 43.91 | 45.57 |  | 8.78 | 9.11 | 0.7055 |
| G6PD | Pentose phosphate pathway | 114.36 | 110.98 |  | 22.87 | 22.20 | 0.7117 |
| ATP6V1G1 | Oxidative phosphorylation | 53.20 | 54.61 |  | 10.64 | 10.92 | 0.7163 |
| ADSL | Purine metabolism | 123.60 | 119.74 |  | 24.72 | 23.95 | 0.7204 |
| NUDT2 | Purine metabolism | 14.79 | 14.36 |  | 2.96 | 2.87 | 0.7264 |
| PANK4 | Pantothenate and CoA biosynthesis | 32.79 | 33.63 |  | 6.56 | 6.73 | 0.7297 |
| LARS2 | Aminoacyl-tRNA biosynthesis | 62.69 | 64.91 |  | 12.54 | 12.98 | 0.7325 |
| DUT | Pyrimidine metabolism | 151.95 | 149.05 |  | 30.39 | 29.81 | 0.7377 |
| IDH3G | Citrate cycle (TCA cycle) | 51.22 | 49.41 |  | 10.24 | 9.88 | 0.7436 |
| CRYL1 | Pentose and glucuronate interconversions | 22.85 | 23.78 |  | 4.57 | 4.76 | 0.7456 |
| CTPS2 | Pyrimidine metabolism | 58.19 | 56.95 |  | 11.64 | 11.39 | 0.7504 |
| PPA1 | Oxidative phosphorylation | 407.07 | 396.34 |  | 81.41 | 79.27 | 0.7536 |
| DTYMK | Pyrimidine metabolism | 148.85 | 143.58 |  | 29.77 | 28.72 | 0.7641 |
| ACACA | Fatty acid biosynthesis | 38.23 | 38.98 |  | 7.65 | 7.80 | 0.7841 |
| PGM3 | Amino sugar and nucleotide sugar metabolism | 55.48 | 56.83 |  | 11.10 | 11.37 | 0.8071 |
| GOT2 | Arginine biosynthesis | 514.28 | 520.25 |  | 102.86 | 104.05 | 0.8072 |
| QPRT | Nicotinate and nicotinamide metabolism | 265.36 | 261.88 |  | 53.07 | 52.38 | 0.8098 |
| HADHA | Fatty acid elongation | 451.82 | 444.80 |  | 90.36 | 88.96 | 0.8142 |
| BLVRB | Riboflavin metabolism | 133.04 | 136.35 |  | 26.61 | 27.27 | 0.8229 |
| HMBS | Porphyrin and chlorophyll metabolism | 53.12 | 51.94 |  | 10.62 | 10.39 | 0.8364 |
| AK3 | Purine metabolism | 333.74 | 328.36 |  | 66.75 | 65.67 | 0.8426 |
| BDH2 | Synthesis and degradation of ketone bodies | 31.48 | 30.44 |  | 6.30 | 6.09 | 0.8477 |
| GMDS | Fructose and mannose metabolism | 153.66 | 156.34 |  | 30.73 | 31.27 | 0.8682 |
| NT5C3 | Purine metabolism | 58.60 | 58.12 |  | 11.72 | 11.62 | 0.8818 |
| HSD17B4 | Primary bile acid biosynthesis | 259.68 | 261.84 |  | 51.94 | 52.37 | 0.8830 |
| MAT2B | Cysteine and methionine metabolism | 27.99 | 28.54 |  | 5.60 | 5.71 | 0.8853 |
| NFS1 | Thiamine metabolism | 67.87 | 67.21 |  | 13.57 | 13.44 | 0.8927 |
| HMGCL | Synthesis and degradation of ketone bodies | 72.11 | 71.35 |  | 14.42 | 14.27 | 0.9034 |
| MIF | Tyrosine metabolism | 2520.76 | 2494.91 |  | 504.15 | 498.98 | 0.9144 |
| NDUFB10 | Oxidative phosphorylation | 267.99 | 269.51 |  | 53.60 | 53.90 | 0.9197 |
| NAMPT | Nicotinate and nicotinamide metabolism | 66.23 | 66.81 |  | 13.25 | 13.36 | 0.9280 |
| ATP6V1E1 | Oxidative phosphorylation | 96.53 | 95.52 |  | 19.31 | 19.10 | 0.9324 |
| MBOAT7 | Glycerophospholipid metabolism | 88.36 | 87.75 |  | 17.67 | 17.55 | 0.9340 |
| HEXB | Other glycan degradation | 218.25 | 217.25 |  | 43.65 | 43.45 | 0.9418 |
| PGLS | Pentose phosphate pathway | 110.67 | 110.12 |  | 22.13 | 22.02 | 0.9438 |
| TKT | Pentose phosphate pathway | 637.58 | 634.59 |  | 127.52 | 126.92 | 0.9472 |
| INPP1 | Inositol phosphate metabolism | 36.82 | 36.64 |  | 7.36 | 7.33 | 0.9495 |
| PPA2 | Oxidative phosphorylation | 257.82 | 258.79 |  | 51.56 | 51.76 | 0.9505 |
| ADH5 | Glycolysis / Gluconeogenesis | 75.28 | 75.80 |  | 15.06 | 15.16 | 0.9529 |
| MECR | Fatty acid elongation | 33.74 | 33.91 |  | 6.75 | 6.78 | 0.9566 |
| ME2 | Pyruvate metabolism | 102.18 | 102.71 |  | 20.44 | 20.54 | 0.9675 |
| SCP2 | Primary bile acid biosynthesis | 60.91 | 60.59 |  | 12.18 | 12.12 | 0.9678 |
| DLST | Citrate cycle (TCA cycle) | 233.98 | 233.49 |  | 46.80 | 46.70 | 0.9679 |
| MDH2 | Citrate cycle (TCA cycle) | 1713.68 | 1718.39 |  | 342.74 | 343.68 | 0.9714 |
| GALK1 | Galactose metabolism | 89.87 | 90.24 |  | 17.97 | 18.05 | 0.9717 |
| ACAA2 | Fatty acid elongation | 461.63 | 462.03 |  | 92.33 | 92.41 | 0.9854 |
