# Supplementary material for: Effects of canagliflozin on growth and metabolic reprograming in hepatocellular carcinoma cells: Multi-omics analysis of metabolomics and absolute quantification proteomics (iMPAQT)
Source: PLoS One. 2020 Apr 28;15(4):e0232283. doi: 10.1371/journal.pone.0232283 (PMC7188283; doi:10.1371/journal.pone.0232283)
Supplement: Attachment — Submitted filename: Responses to REVIEWER 2.pdf [file pone.0232283.s013.pdf]

Responses to REVIEWER 2,

Thank you for your comments regarding our manuscript (Manuscript PONE-D-19-33203). We appreciate your comments, which have helped us to improve our manuscript. In line with the comments, please find below our point-by-point responses.

Reviewer #2:

1. In the discussion, “it remains unclear if the effects of CANA on cell proliferation, metabolomics, and proteomics are cancer specific and are SGLT2 dependent.” Authors could examine the effects of knockdown/knockout of SGLT2.

Answer: Following your suggestion, we performed knock down of SGLT2 using small interfering RNA (siRNA) (Silencer® Select s534032 Thermo Fisher), because knock down is reported to be suitable for investigating phenotypic change than knock out [1, 2].

We transfected siRNA for SGLT2 into Hep3B cells according to the manufactures’ instruction. However, there was no significant depletion in protein expression of SGLT2 in the siRNA group compared to the control group (Appendix Figure 1). Although we performed the siRNA experiment twice at different concentration of siRNA (5 nM and 10 nM), no significant difference was seen in protein expression of SGLT2 in the siRNA group compared to the control group (Appendix Figure 1). The reason for the unsuccessful depletion of SGLT2 protein expression remains unclear, a possible explanation is following: Although expression of mRNA for SGLT2 was decreased by siRNA treatment, expression of mRNA for SGLT2 was detected even in Hep3B cells treated with 10 nM of siRNA (Appendix Figure 2). These data suggest that a decrease in mRNA for SGLT2 was not sufficient for depletion of protein expression of SGLT2. Thus, it is difficult to examine effects of SGLT2 on metabolisms and cell proliferation by siRNA for SGLT2 in Hep3B cells at this moment. To clarify this issue, further study will be focused on an alternating approach such as short hairpin RNA, which causes more continuous suppression of target protein than

siRNA [3]. This issue was described as a limitation of this study (Page 38, line 17-19).

Method for Appendix Figure 1 and 2

Knockdown of SGLT2 in Hep3B using small interfering RNA (siRNA) Hep3B cells were seeded into six-well plates at a density of 1×105 cells/well. The cells reached 80% confluence 48 hours after incubation and were transiently transfected with siRNA using Lipofectamine® 2000 (Invitrogen; Thermo Fisher Scientific, Inc.). The sequences for siRNA (Invitrogen; Thermo Fisher Scientific, Inc.) were as follows: Sense, 5′-CCGGAGCUGUAUUCAUCCATT-3′ and anti-sense, 5′-UGGAUGAAUACAGCUCCGGAG-3. Cell transfection was performed, according to the manufactures' instruction. Briefly, each sequence of 10 μM siRNA (1.5 μL) and 9 µL Lipofectamine® 2000 was diluted in serum-free medium (300 µL) at room temperature for 5 min, mixed together. The mixture was subsequently administered to the Hep3B cells, then the medium was replaced with complete medium. Hep3B cells were seeded into six-well plates at a density of 5×104 cells/well and protein expression of SGLT2 was evaluated at 24, 48, 72, 96, and 120 hours after incubation.

Appendix figure 1

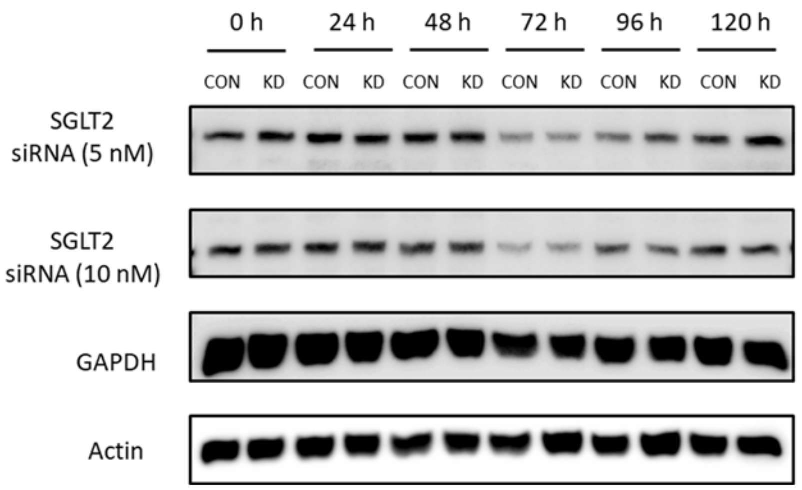

Appendix figure 2

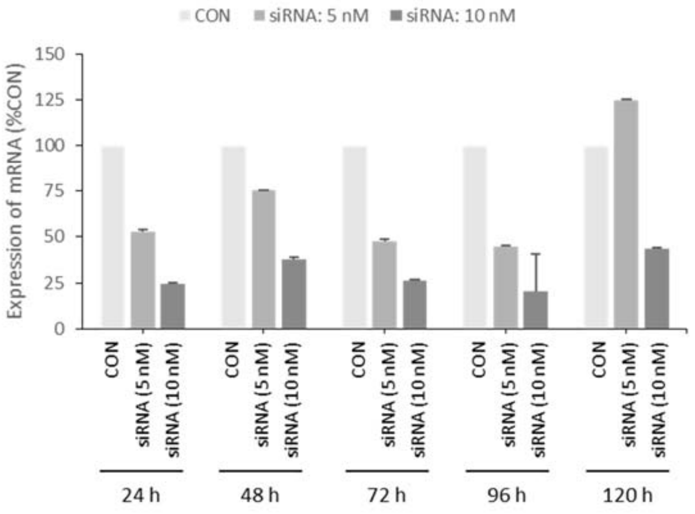

2. Please mention if the effects are CANA specific or common features of SGLT2 inhibitors.

Answer: We appreciate for your comment. As you suggested, we examined the effect of dapagliflozin (10μM), a SGLT2 inhibiter, on cell proliferation in Hep3B. There was no significant difference in cell number between the dapagliflozin and control groups (supplementary figure 3). Moreover, Obara et al. reported that tofogliflozin, a SGLT2 inhibiter, did not suppress cell number in Huh7 and JHH cells [4]. Hung MH et al. reported that CANA specifically inhibited cell proliferation due to β-catenin-related pathway, which was not seen in the dapagliflozin and empagliflozin groups in Huh7 and Hep3B [5]. In this study, CANA-specific mechanism on inhibition of cell proliferation remains unclear; however, these previous reports along with our additional data suggest that suppression of cell proliferation in hepatoma cells may be CANA specific effect. This discussion was added in the revised manuscript (Page 37, line 16-Page38, line 2).

References

1. Ma Z, Zhu P, Shi H, Guo L, Zhang Q, Chen Y, et al. PTC-bearing mRNA elicits a genetic compensation response via Upf3a and COMPASS components. Nature. 2019;568(7751):259-63. doi: 10.1038/s41586-019-1057-y. PubMed PMID: 30944473.
2. El-Brolosy MA, Kontarakis Z, Rossi A, Kuenne C, Gunther S, Fukuda N, et al. Genetic compensation triggered by mutant mRNA degradation. Nature. 2019;568(7751):193-7. doi: 10.1038/s41586-019-1064-z. PubMed PMID: 30944477; PubMed Central PMCID: PMCPMC6707827.
3. Yang X, Wu X, Yang Y, Gu T, Hong L, Zheng E, et al. Improvement of developmental competence of cloned male pig embryos by short hairpin ribonucleic acid (shRNA) vector-based but not small interfering RNA (siRNA)-mediated RNA interference (RNAi) of Xist expression. J Reprod Dev. 2019;65(6):533-9. doi: 10.1262/jrd.2019-070. PubMed PMID: 31631092; PubMed Central PMCID: PMCPMC6923154.
4. Obara K, Shirakami Y, Maruta A, Ideta T, Miyazaki T, Kochi T, et al. Preventive effects of the sodium glucose cotransporter 2 inhibitor tofogliflozin on diethylnitrosamine-induced liver tumorigenesis in obese and diabetic mice. Oncotarget. 2017;8(35):58353-63. doi: 10.18632/oncotarget.16874. PubMed PMID: 28938561;

PubMed Central PMCID: PMCPMC5601657.
5. Hung MH, Chen YL, Chen LJ, Chu PY, Hsieh FS, Tsai MH, et al. Canagliflozin inhibits growth of hepatocellular carcinoma via blocking glucose-influx-induced beta-catenin activation. Cell Death Dis. 2019;10(6):420. doi: 10.1038/s41419-019-1646-6. PubMed PMID: 31142735; PubMed Central PMCID: PMCPMC6541593.
