# Supplementary material for: Effects of canagliflozin on growth and metabolic reprograming in hepatocellular carcinoma cells: Multi-omics analysis of metabolomics and absolute quantification proteomics (iMPAQT)
Source: PLoS One. 2020 Apr 28;15(4):e0232283. doi: 10.1371/journal.pone.0232283 (PMC7188283; doi:10.1371/journal.pone.0232283)
Supplement: Attachment — Submitted filename: Responses to REVIEWER 3.docx [file pone.0232283.s012.docx]

Responses to REVIEWER 3,

Thank you for your comments regarding our manuscript (Manuscript PONE-D-19-33203). We appreciate your comments, which have helped us to improve our manuscript. In line with the comments, please find below our point-by-point responses.

1) Figure 2C, D; Live cell rate is decreased in CANA 10uM group. However, dead cell numbers in CANA 10uM group are not different from those in CON group. It seems that there is discrepancy between these data. Is dead cell rate in CANA 10uM group same with CON group?

**Answer:** We apologize for wrong description in vertical line in Figure 2C. In the figure, we examined number of live cells in CANA 10 μM by comparing to the CON group and the result was expressed by %CON. We corrected this mistake in the revised manuscript (Figure 2C and D). Again, we apologize for causing confusion with the wrong description.

2) In the study using iMPAQT, the dose of CANA should be described.

**Answer:** We apologize for insufficient description in the study using iMPAQT. The dose of CANA (10 μM) was added in the revised manuscript (Page 14, line 12-13).

3) Metabolic analysis and iMPAQT analysis is performed only in Hep3B cells. The result may be specific in this cell line. The validation using the other cell line should be included.

**Answer:** We totally agree with your comment. Expression of SGLT2 was seen in both Hep3B and Huh 7 cells, and CANA inhibited cell proliferation of both HCC cell lines. We examined changes in phosphorylation of AMPKα1 and ACC and found that CANA phosphorylated AMPKα1 and ACC not only in Hep3B cell, but also in Huh 7 cells (S5 Fig). However, as you indicated, iMPAQT analysis was performed only in Hep3B cells. Therefore, the result may be specific in this cell line. The validation study using the various HCC cell lines should be performed. Unfortunately, we could not conduct the validation study at this moment. Therefore, this issue was described in the Discussion section of the revised manuscript (Page 32 line 7–9; Page 37 line 4-7).

4) The data is not shown the alteration in mitochondrial oxidative phosphorylation metabolism, fatty acid metabolism, and purine and pyrimidine metabolism really affects cell proliferation.

**Answer:** We totally agree with your comment. We did not present direct evidence for the link between altered pathways and reduced cell proliferation in this study. In this study, CANA mainly altered following pathways 1) oxidative phosphorylation/fatty acid metabolism pathway through down-regulation of ATP synthase F1 subunit alpha and 2) purine and pyrimidine metabolism though down-regulation of NME1 and PRIM2. In order to proof the direct evidence, up-regulation of ATP synthase F1 subunit alpha, NME1, and PRIM2 thought to be required. However, there is no available drugs, which activate these three molecules simultaneously. Thus, it seems impossible to proof the direct evidence currently. As far as we searched, there is no study, which proof the direct evidence for the link between altered pathways and reduced cell proliferation by up-/down-regulation of multiple pathways [1]. Therefore, we described this issue as a limitation of this study (Page 37, line 22-Page 38, line 5).

**Reference**

1.	Yu L, Wu J, Zhai Q, Tian F, Zhao J, Zhang H, et al. Metabolomic analysis reveals the mechanism of aluminum cytotoxicity in HT-29 cells. PeerJ. 2019;7:e7524. doi: 10.7717/peerj.7524. PubMed PMID: 31523502; PubMed Central PMCID: PMCPMC6716502.
